# Validation strategy of a bioinformatics whole genome sequencing workflow for Shiga toxin-producing *Escherichia coli* using a reference collection extensively characterized with conventional methods

**DOI:** 10.1099/mgen.0.000531

**Published:** 2021-03-03

**Authors:** Bert Bogaerts, Stéphanie Nouws, Bavo Verhaegen, Sarah Denayer, Julien Van Braekel, Raf Winand, Qiang Fu, Florence Crombé, Denis Piérard, Kathleen Marchal, Nancy H. C. Roosens, Sigrid C. J. De Keersmaecker, Kevin Vanneste

**Affiliations:** ^1^​ Transversal activities in Applied Genomics, Sciensano, Brussels, Belgium; ^2^​ Department of Information Technology, IDLab, Ghent University, IMEC, Ghent, Belgium; ^3^​ Department of Plant Biotechnology and Bioinformatics, Ghent University, Ghent, Belgium; ^4^​ National Reference Laboratory for Shiga toxin-producing *Escherichia coli* (NRL STEC), Foodborne Pathogens, Sciensano, Brussels, Belgium; ^5^​ National Reference Center for Shiga toxin-producing *Escherichia coli* (NRC STEC), Brussels, Belgium; ^6^​ Department of Genetics, University of Pretoria, Pretoria, South-Africa

**Keywords:** *Escherichia coli*, whole genome sequencing, STEC, foodborne pathogens, validation, public health

## Abstract

Whole genome sequencing (WGS) enables complete characterization of bacterial pathogenic isolates at single nucleotide resolution, making it the ultimate tool for routine surveillance and outbreak investigation. The lack of standardization, and the variation regarding bioinformatics workflows and parameters, however, complicates interoperability among (inter)national laboratories. We present a validation strategy applied to a bioinformatics workflow for Illumina data that performs complete characterization of Shiga toxin-producing *Escherichia coli* (STEC) isolates including antimicrobial resistance prediction, virulence gene detection, serotype prediction, plasmid replicon detection and sequence typing. The workflow supports three commonly used bioinformatics approaches for the detection of genes and alleles: alignment with blast+, kmer-based read mapping with KMA, and direct read mapping with SRST2. A collection of 131 STEC isolates collected from food and human sources, extensively characterized with conventional molecular methods, was used as a validation dataset. Using a validation strategy specifically adopted to WGS, we demonstrated high performance with repeatability, reproducibility, accuracy, precision, sensitivity and specificity above 95 % for the majority of all assays. The WGS workflow is publicly available as a ‘push-button’ pipeline at https://galaxy.sciensano.be. Our validation strategy and accompanying reference dataset consisting of both conventional and WGS data can be used for characterizing the performance of various bioinformatics workflows and assays, facilitating interoperability between laboratories with different WGS and bioinformatics set-ups.

## Data Summary

The datasets supporting the conclusions of this study have been deposited in the NCBI SRA under accession number PRJNA633966 (in-house sequenced data), Zenodo (10.5281/zenodo.4006065) (results of all bioinformatics analyses), and are included within this paper and its Supplementary Files (results of the validation). The authors confirm all supporting data and protocols have been provided within the article or through supplementary data files.

**Impact Statement**

Whole genome sequencing (WGS) is rapidly being integrated for routine surveillance for a wide variety of pathogens in public health settings. However, its successful integration is hindered by a lack of standardized guidelines and quality criteria for bioinformatics workflows, which complicates collaboration between laboratories. We present a bioinformatics workflow for the complete characterization of Shiga toxin-producing *Escherichia coli* isolates, developed with the aim of routine usage by non-expert bioinformaticians from laboratories operating under a strict quality system. The performance of the workflow was extensively validated on a large set of samples, characterized with molecular methods, demonstrating overall high performance. Both our validation strategy and generated reference dataset, including metadata confirmed with conventional methods, are of particular interest to aid other laboratories with the implementation and validation of their WGS workflows. Moreover, a ‘push-button’ implementation of our bioinformatics workflow has been made available on the public Galaxy instance of our institute for non-profit usage, coupled with a YouTube tutorial video detailing the pipeline usage and interpretation of results.

## Introduction

Whole genome sequencing (WGS) has revolutionized foodborne outbreak investigation and surveillance of a wide variety of microbial pathogens [1]. The characterization of isolates using conventional microbiological methods requires several different labour-intensive molecular and other assays, and can take several days to complete. In contrast, WGS can provide a complete overview of an isolate with all required information for pathogen typing and characterization, including detection of genes encoding antimicrobial resistance (AMR) and virulence factors, serotype prediction, plasmid detection and sequence typing, in a relatively short period (3–5 days) with single-nucleotide resolution and at a relatively low cost per sample [2]. Moreover, WGS has also enabled novel phylogenetic inference methods such as core genome multi-locus sequence typing (cgMLST) and whole-genome SNP (wgSNP) analysis that provide much more discriminatory power to delineate strains compared to conventional methods such as PFGE or multiple-locus variable-number tandem repeat analysis (MLVA) [3–5], providing added value for quick and accurate outbreak resolution. WGS-based methods for relatedness investigation can be scaled up from case-by-case applications to routine surveillance, as illustrated by EnteroBase for cgMLST [6], and SnapperDB for wgSNP [7] analysis.

Because of these advantages, the use of WGS for pathogen typing in both outbreak situations and routine surveillance is becoming more widespread, with many national reference centres (NRCs, human) and laboratories (NRLs, food and feed) integrating it into their routine activities [1, 8, 9]. This is, however, not always straightforward because of, among others, lack of sufficient bioinformatics expertise and the requirement for validation of WGS-based methods by enforcement and other (clinical) laboratories that operate according to strict quality systems. The first hurdle can be tackled by the increasing availability of web-based tools that allow non-expert bioinformaticians to analyse their data without the need for command-line experience or specialized hardware. The Center for Genomic Epidemiology (CGE) provides a widely used set of such tools including, among others, AMR characterization [10, 11], virulence gene detection [12], plasmid replicon detection [13] and serotype determination [14]. The PathoSystems Resource Integration Center (PATRIC) website provides a broad array of analyses and is applicable to all bacterial species, with a focus on pathogens [15]. A multitude of web-based alternatives exists for AMR characterization [16], plasmid detection [17] and sequence typing [6, 18]. Specialized portals for WGS-based analysis of specific species also exists, such as EnteroBase [6] and ARIES [19] for *Escherichia coli *, providing more comprehensive solutions for analysing WGS data. The second hurdle, i.e. the need for validation of bioinformatics assays to demonstrate that they are ‘fit-for-purpose’ and adhere to certain predefined quality characteristics, as also specifically required to obtain International Organization for Standardization (ISO) accreditation, should be addressed to facilitate the exchange of raw WGS data but also to infer results such as isolate characteristics and relatedness. The bioinformatics methodology can differ, and multiple algorithmic approaches exist to compare WGS data against databases containing information on AMR, virulence, cgMLST, etc. Three widely used methodologies to search against such databases are: (i) *de novo* assembly followed by alignment with blast+ [20]; (ii) kmer-based read mapping with tools such as KMA [21]; and (iii) direct read mapping with tools such as SRST2 [22]. However, performance differences between these three approaches have not been extensively evaluated.

As outbreak investigations usually involve several different laboratories, often across different countries, harmonization of employed methods is essential to link clinical cases to suspected food sources. The need for validation of WGS-based workflows has therefore recently started to receive more attention, in particular how to approach this relatively novel technology in light of the more traditional concept of validation of conventional molecular biology-based methods. Consequently, recent studies have showcased validation strategies for end-to-end WGS workflows [23], adaptation of the same workflow in different labs [24], the general WGS process subdivided into its individual components (library preparation, sequencing, analysis, etc.) [25], and also specifically the bioinformatics component [26]. This is also part of the scope of the ISO working group ISO TC34-SC9-WG25, which is preparing general requirements and guidelines for WGS to type and genomically characterize foodborne bacteria [27] [ISO 23418 : 2018(E)]. However, a widely accepted consensus is currently still missing. In particular, the reference standard to employ for validation of WGS-based workflows, i.e. a dataset for which the ‘ground truth’ is known and can be used to evaluate the performance of WGS, remains problematic. High-quality reference WGS datasets for which information from conventional methods and/or epidemiological links are available remain scarce [14, 28]. This information is typically lacking in publicly available WGS data for which metadata (used here to refer to the intrinsic characteristics of the isolate such as the AMR or virulence profile rather than host information) often is inconsistent or missing, hampering its use for systematic evaluation. This is partly due to the fact that constructing such databases requires employing labour- and cost-intensive molecular biology-based methods such as PCR amplification and/or Sanger sequencing, which is considered a gold standard for confirming the absence/presence of genomic markers of interest (e.g. AMR gene, cgMLST allele) [29]. Currently available reference datasets are often limited to a single aspect such as the AMR profile [30, 31], the presence of virulence factors [12], serotype [14] and known epidemiological links of outbreaks [32, 33]. Efforts by organizations such as the Global Microbial Identifier (GMI) and ISO therefore aim not only to provide guidelines to standardize WGS validation, but also databases with high-quality metadata linked to genomic information [34].

Recently, the European Food Safety Authority (EFSA) highlighted the necessity of a harmonized and quality-controlled WGS-based system for investigation of cross-country outbreaks and risk assessment of foodborne pathogens, employing Shiga toxin-producing *Escherichia coli* (STEC) as a case study for switching to WGS [35]. STEC is a rapidly evolving human enteric pathogen responsible for foodborne infections that can lead to gastroenteritis, diarrhoea and haemolytic uremic syndrome (HUS), and may even be fatal [36]. The added value of WGS was highlighted in particular by the German 2011 outbreak of the virulent O104:H4 STEC strain resulting in 3816 cases, including 845 HUS cases and 54 deaths [37]. Conventional molecular biology-based assays failed to resolve the outbreak whereas WGS managed to provide a complete overview of events that had resulted in the emergence of this particularly pathogenic outbreak strain. Since then, the benefit of WGS for STEC characterization for foodborne outbreaks and routine surveillance has been illustrated extensively [8, 12, 38, 39].

Here, we present the validation of a bioinformatics workflow to fully characterize STEC and other *E. coli* isolates, exhaustively validated by extending a previously described validation framework for the bioinformatics component of WGS workflows [26]. A set of 137 isolates for which information based on conventional methods was available for the AMR profile, presence of virulence genes and serotype was sequenced using the Illumina MiSeq to create a high-quality reference dataset of 131 samples against which the performance of the bioinformatics workflow was validated for all assays. Moreover, three different bioinformatics approaches were evaluated based on (i) *de novo* assembly with SPAdes followed by alignment with blast+; (ii) kmer-based read mapping with KMA; and (iii) direct read mapping with SRST2.

## Methods

### Bioinformatics workflow

#### Data (pre-)processing and quality control

**Fig. 1.**
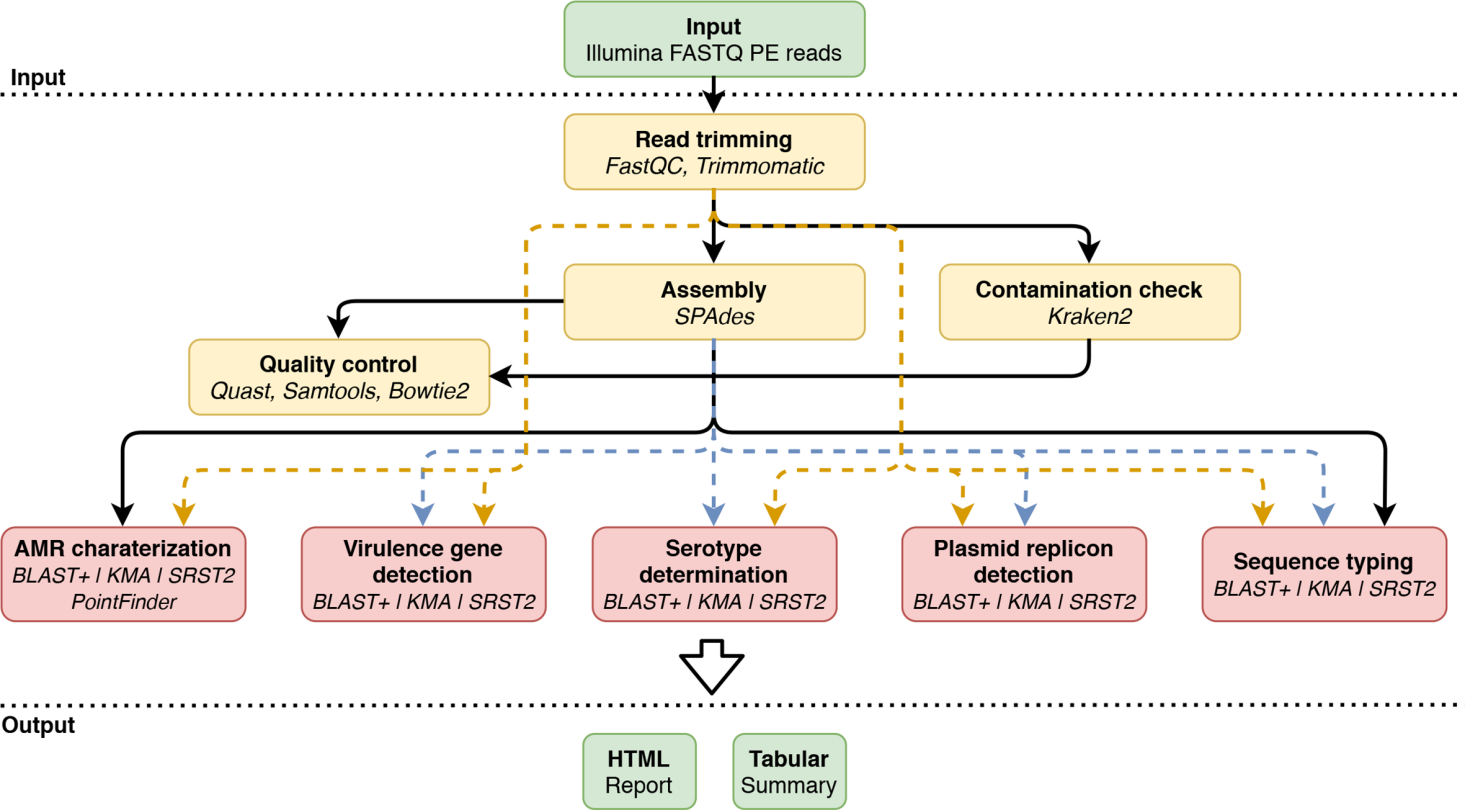
Overview of the bioinformatics workflow. Each box represents a component corresponding to a series of tasks that provide a certain well-defined functionality (indicated in bold). Major bioinformatics software packages employed in each module are also mentioned (indicated in italics). Data processing steps are indicated in yellow, and bioinformatics assays are indicated in red. Data flows specific to blast+ are indicated with blue dashed lines, and data flows for KMA and SRST2 with orange dashed lines. PE, paired-end.

Fig. 1 provides an overview of the bioinformatics workflow, which is compatible with WGS data generated using Illumina sequencers. Data pre-processing and quality control are executed as previously described by Bogaerts *et al*. [26] with updates to the most recent tool versions of underlying tools during development (only changed options and versions are mentioned below). Briefly, first pre-trimming quality reports are generated using FastQC 0.11.5 (https://www.bioinformatics.babraham.ac.uk/projects/fastqc/), before reads undergo quality trimming using Trimmomatic 0.38 [40], and post-trimming quality reports with FastQC are created. Genome assembly is done using SPAdes 3.13.0 [41]. Two specific extra filtering steps are performed in line with recommendations of the European Center for Disease Prevention and Control [42]. Firstly, the ‘--cov_cutoff’ parameter is set to 10 to filter out contigs with a (kmer) coverage lower than 10. Secondly, contigs smaller than 1000 bases are removed using seqtk seq 1.2 (https://github.com/lh3/seqtk) using the ‘-L’ option. Assembly statistics are calculated on the filtered assembly with QUAST 4.4 [43]. The processed reads are mapped against the assembly using Bowtie2 2.3.0 [44] with the ‘--end-to-end’, ‘--sensitive’ and ‘--phred33’ options enabled, and used to estimate the coverage with SAMtools depth 1.9 [45]. Several quality metrics are then computed, for which warning and failure thresholds were defined by selecting more and less stringent values for metrics exhibiting less and more variation between samples, respectively, for which an overview is presented in Table 1. Additionally, Kraken2 2.0.7 [46] is used to check against contamination with default parameters and a database containing all NCBI RefSeq Genome entries (database accessed 18 February 2019) annotated as ‘complete genome’ with accession prefixes NC, NW, AC, NG, NT, NS and NZ of the following taxonomic groups: archaea, bacteria, fungi, human, protozoa and viruses.

**Table 1.** Quality control metrics of the bioinformatics workflow

Metric	Definition	Warning threshold	Failure threshold
Contamination	Percentage of reads classified as highest occurring in species other than *E. coli *	1 %	5 %
Median coverage against assembly	Median coverage based on mapping of the trimmed reads against the assembled contigs	20	10
% cgMLST genes identified	Percentage of cgMLST genes identified. Only perfect hits (i.e. full length and 100 % identity) are considered [85]	95	90
Average read quality (Q-score)	Q-score of the trimmed reads averaged over all reads and positions	30	25
GC-content deviation	Deviation of the average GC content of the trimmed reads from the expected value for *E. coli* (50.5% [86])	2 %	4 %
N-content	Average N-fraction per read position of the trimmed reads, expressed as a percentage	0.5 %	1 %
Per base sequence content	Difference between AT and GC frequencies averaged at every read position. Since primer artefacts can cause fluctuations at the start of reads due to the non-random nature of enzymatic tagmentation when the Nextera XT protocol is used for library preparation, the first 20 bases are not included in this test. As fluctuations can also exist at the end of reads caused by the low abundance of very long reads because of read trimming, the 0.5 % longest reads are similarly excluded	3 %	6 %
Minimum read length	Minimum read length after trimming (denoted as a percentage of untrimmed read length) that a minimum of half of all trimmed reads must obtain (e.g. half of all trimmed reads should either be minimally 120 or 200 bases long when raw input reads lengths are 300 bases long)	66.67 %	40.00 %

#### Genotypic AMR detection

The ResFinder [10] database is used for AMR gene detection. The database is clustered beforehand at 80 % identity using CD-HIT 4.6.8 [47] to limit reported genes to one per cluster. The database is automatically pulled in-house and updated weekly to ensure up-to-date results (the date of the last update is included in the output report). The workflow supports alignment-based detection using blast+ 2.6.0 against the assembly [20], kmer-based detection using KMA 1.2.25 [21] and read mapping-based detection using SRST2 0.2.0 [22]. For blast+, assembled contigs are aligned to the database using blastn with the ‘-task’ option set to ‘blastn’, and only hits with ≥90 % identity and ≥60 % target coverage are retained. The best hit for each cluster is determined using the allele scoring method described by Larsen *et al*. [48]. Visualizations of pair-wise alignments are extracted from the blast output generated with the pairwise output format (‘-outfmt 1’). For KMA, trimmed paired-end reads are provided as input with otherwise default settings. Only hits with ≥90 % identity and ≥60 % target coverage are retained, and the best hit for each cluster is selected based on the score calculated by KMA. For SRST2, trimmed paired-end reads are provided as input, and the best hits are selected by SRST2 with the ‘--min_coverage’ parameter set to 60 and ‘--max_divergence’ set to 10. The latter was used as an alternative to the percentage sequence identity used by the other detection methods, which is not calculated by SRST2. A local installation of PointFinder [11] (https://bitbucket.org/genomicepidemiology/pointfinder, accessed 27 February 2019) is used to screen for AMR-associated point mutations using the assembly as input and setting the ‘--method’ parameter to ‘blastn’. The underlying PointFinder database is not automatically updated due to potential incompatibilities between the updated database format and tool version. Detected mutations, associated resistance and PubMed identifiers (when available) are also provided in the output report.

#### Virulence gene detection

Virulence genes are detected using the *stx* and *E. coli* databases from VirulenceFinder [12] and using the same workflow as described for genotypic AMR detection, but with the minimum target coverage set to ≥90 %. The underlying databases are pulled in-house and updated weekly with the last update date mentioned in the output report.

#### Serotype determination

Serotype determination is performed by first identifying H- and O-type determining genes using the SerotypeFinder database [14] through the same methodology as described for genotypic AMR detection but with the minimum sequence identity set to ≥85 % for blast+ and KMA, and divergence set to ≥85 % for SRST2. Databases for the H- and O-type determining genes are automatically pulled in-house and updated weekly with the last database update date mentioned in the output report. The H- and O-types are then determined based on the decision rules shown in Fig. S1 (available in the online version of this article). The detected serotype is listed in the output report below the tables with the detected genes for the O- and H-type.

#### Plasmid replicon detection

Plasmid replicons are detected using the PlasmidFinder database for *Enterobacteriaceae* [49], using the same workflow as described for genotypic AMR detection but with the following changes: minimum percentage identity for blast+ and KMA detection set at ≥95 % instead of ≥90 % in accordance with default recommendations for plasmid replicon detection. For SRST2, a filter is applied to remove hits with ≥5 % divergence. The database is automatically pulled in-house and updated weekly with the last database update date mentioned in the output report.

#### Sequence typing

The cgMLST scheme from EnteroBase [6] is used for (genotypic) sequence typing using the same workflow as described for genotypic AMR detection, typing each locus separately. All sequences and profiles are automatically pulled in-house and updated weekly with the last database update date mentioned in the output report. For blast+, detection is analogous to the gene detection workflow, but selection of the best hit is done per locus instead of database cluster. If multiple exact matches exist, the longest one is reported. For KMA, trimmed paired-end reads are used as input and the best hit for each locus is extracted from the tabular output files. If sequence type definitions are available and the detected allele combination matches a known sequence type, this is reported in the output. Typing with SRST2 using cgMLST schemes is not supported because of runtime requirements, but classic MLST typing is supported and is performed using the ‘--mlst-db’ option with default settings for classical MLST schemes (see section below).

#### Implementation and availability

The workflow is implemented in Python 3.7.5 and runs on a (virtualized) Ubuntu 18.04 (64-bit) server. The workflow output is provided as an interactive HTML report with the most relevant information and links to the full output of the different bioinformatics assays, enabling further processing or in-depth investigation. A tabular summary file is also provided, containing an accumulation of the most relevant statistics and results in a tab-separated format that can be useful for programmatic processing. The workflow is integrated as a stand-alone tool in an in-house Galaxy Workflow Management System instance [50], and requires only uploading the data, setting the pipeline detection method (blast+, KMA or SRST2), and selecting the desired bioinformatics assays. A ‘push-button’ implementation of this workflow is also available as a free resource for academic and non-profit usage (registration required) at the public Galaxy instance of our institute at https://galaxy.sciensano.be. Usage of the workflow through Galaxy is explained in a training video that is available on YouTube (https://youtu.be/FLCM-BbzIBY). The sample ‘STEC_DEMO’ in this video corresponds to sample EH1380 (SRR11816069) from the validation dataset. A screenshot of the interface is provided in Fig. S2. All output reports of this workflow on the validation dataset have been made available in Zenodo (see data statement). Besides the validated assays discussed in this paper, our workflow also allows other bioinformatics assays, which were not validated for routine purposes but that can nonetheless be used for informative purposes, such as resistance gene detection with alternative AMR databases (NDARO [30], ARG-ANNOT [51] and CARD [16]), sequence typing with the regular MLST schemes from Institut Pasteur [52] and the University of Warwick [6] and the INNUENDO cgMLST scheme [53], and variant calling and filtering using the methodology of the CSI phylogeny pipeline [54]. Moreover, the pipeline also supports using Ion Torrent data as input files.

### Validation dataset and characterization with conventional methods

#### Selection of isolates and WGS

**Fig. 2.**
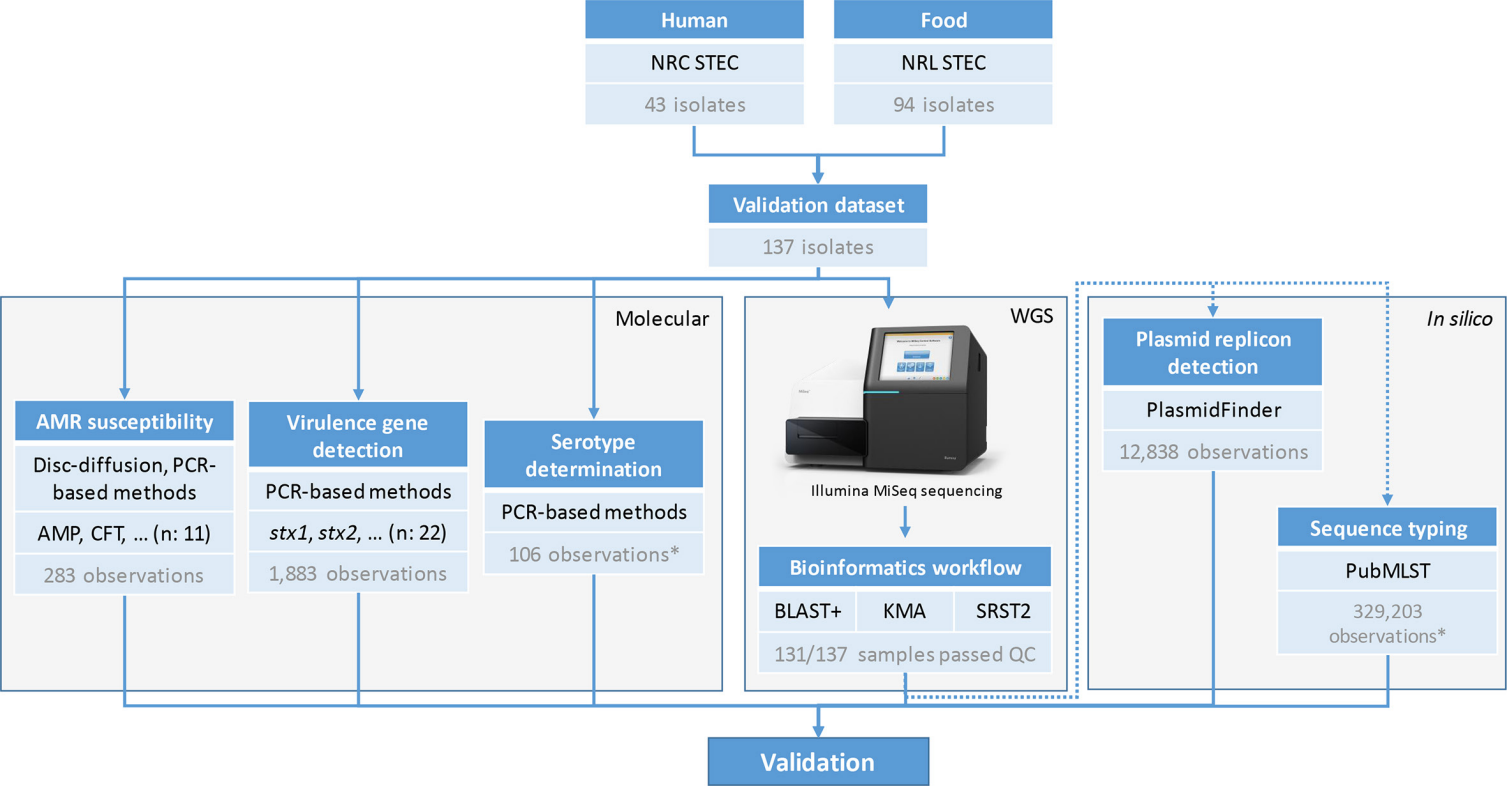
Overview of the characterization of the validation samples. Boxes with blue headers represent different steps in the validation. The number of samples, isolates or observations is indicated at the bottom of each box. The top part of the figure represents the collection of the validation samples from the Belgian NRC and NRL for STEC. The grey boxes group the different steps of the validation: characterization with molecular methods (‘Molecular’), whole genome sequencing (‘WGS’) and *in silico* characterization for assays without reference information from molecular methods (‘*In silico’*). All detected AMR genes with WGS were confirmed to be present with PCR. National Reference Centre (NRC), National Reference Laboratory (NRL), whole genome sequencing (WGS), ampicillin (AMP), cefotaxime (CTF). *Does not include observations from 10 negative control samples from species other than *E. coli *.

**Fig. 3.**
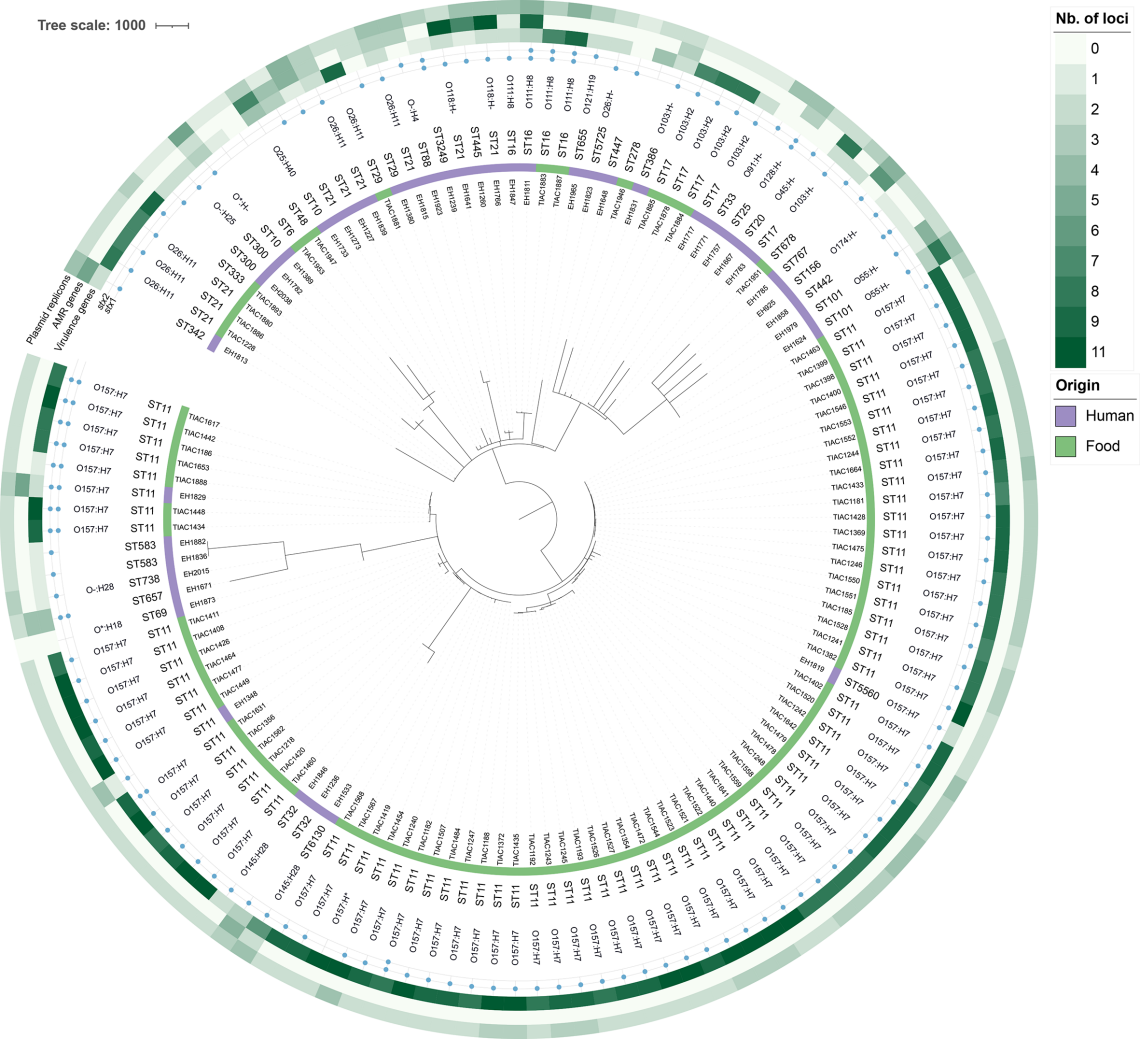
Minimum spanning tree containing an overview of the diversity contained within the validation dataset. The scale bar is expressed as the number of cgMLST allele differences between isolates. The annotations are (from inner to outer rings): sample name, sample origin (human or food according to the colour legend), sequence type determined with the MLST scheme of the University of Warwick using blast+-based detection, O-type and H-type as determined with PCR-based methods (absence indicates that the serotyping determining genes were not tested with PCR), presence of *stx1* and *stx2* as determined with PCR-based methods (a blue circle denotes presence), the number of virulence genes from the set of 20 virulence genes other than *stx1* and *stx2* that were detected with PCR-based methods, the number of AMR genes that were detected with blast+ and confirmed with PCR, and the number of detected plasmid replicons by the reference standard (PlasmidFinder). The number of AMR genes, virulence genes and plasmid replicons are indicated according to the colour legend. Antimicrobial resistance (AMR). Full detailed information on the metadata for the characteristics of the validation dataset is available in the Supplementary Material. *O-types for samples EH1873 and EH1389 were abbreviated to ‘O*‘ from ‘O17/43/44/77/106’ and ‘O90/127’, respectively; H-type for sample TIAC1419 was H21/H7.

The validation dataset consists out of 137 STEC isolates collected from human faeces (*n*: 43) and various food matrices (*n*: 94) sampled between June 1998 and April 2014. Genomic DNA of all isolates was prepared between 2012 and 2014 in the scope of another project using the DNeasy Blood and Tissue kit (Qiagen) according to the manufacturer’s protocol. Each DNA extract was preserved at −20 °C before Nextera XT DNA library preparation (Illumina) according to the manufacturer’s instructions, and subsequently underwent MiSeq sequencing over seven sequencing runs using the MiSeq V3 chemistry (Illumina) for the production of 2×250 bp paired-end reads, aiming for a theoretical coverage of 60× per sample. All sequencing data have been submitted to SRA [55] under BioProject PRJNA633966. The dataset was complemented with negative control samples from species other than *E. coli* retrieved from SRA. An overview of all isolates and corresponding accession numbers is provided in Table S1. A schematic overview of characterization with conventional methods is also provided in Fig. 2. A phylogenomics comparison of all samples of the validation dataset was obtained using the results of the sequence typing assay for cgMLST, after which a minimum spanning tree was constructed using GrapeTree 2.2 [56] with the ‘method’ option set to ‘MSTreeV2’. The resulting phylogenetic tree is provided in Fig. 3, for which sample metadata and statistics on the available reference information were added as annotations and visualized using IToL [57]. Classic MLST sequence type information was extracted from the sequence typing results generated with blast+-based detection.

#### AMR susceptibility

All isolates originating from human faeces, except for one, were phenotypically tested (*n*: 42) for susceptibility to ampicillin, cefotaxime, chloramphenicol, ciprofloxacin, gentamycin, kanamycin, nalidixic acid, streptomycin, sulphonamides, tetracycline and trimethoprim (I2A Diagnostics) using the Kirby-Bauer Disc Diffusion method, according to European Committee on Antimicrobial Susceptibility Testing (EUCAST) recommendations [or on Clinical and Laboratory Standards Institute (CLSI) recommendations when no breakpoints were available from the former]. Observed phenotypic resistance profiles to the 11 antibiotics were combined per antibiotic group (see validation strategy), as indicated in Table S2. Because only phenotypic data were available, all predicted resistance by the bioinformatics workflow was further verified with conventional methods through PCR amplification of the detected AMR genes, and PCR amplification followed by Sanger sequencing of the regions containing point mutations associated with AMR. Applied PCR primer sequences, primer concentrations and PCR conditions are provided in Table S9. The methodology for the Sanger sequencing is described in the Supplementary Methods. Note that predicted sensitivity could not be further investigated due to the imperfect mapping between genotypic features and the resistance phenotype (see Discussion).

#### Detection of virulence genes

The presence or absence of virulence factor encoding genes in human isolates (*n*: 43) was determined according to the routine methods performed at the Belgian NRC for STEC (NRC-STEC) as described previously [58]. In brief, detection of *stx1* and/or *stx2* in human isolates was performed with conventional PCR for strains isolated before 2008 [59–61]. Since these primers did not allow detection of *stx2f*, a multiplex PCR was developed and used from 2008 onwards for the combined detection of *stx1* (a, c, d) and *stx2* (a-g) as described before [62–64]. Moreover, the presence/absence of *eae* and *ehxA* was determined with PCR, as described before [63]. All food isolates (*n*: 94) were genotypically characterized in the scope of another project using a PCR-based method. The presence/absence of *stx1*, *stx2* and 20 other virulence genes [*aaiC, aggR, bfpA, eae, ehxA, ent/espl2, espP, ipaH, katP, lt (ltcA), nleA, nleB, nleE, nleF, nleH1-2, saa, sth, stp, subA* and *terB*] was determined. The different primer sets, primer concentrations and PCR conditions used are described in Table S3. The reference information for genes discrepant with the output of the workflow was updated based on retesting with PCR using the same primers. An overview of all evaluated and detected virulence genes is provided per sample in Table S4.

#### Serotype determination

Most food (*n*: 89) and part of the human isolates (*n*: 27) were genotypically serotyped using PCR-based methods in the scope of another project. The assay discriminated 16 O-groups (O25, O26, O45, O55, O90/127, O91, O103, O104, O111, O113, O118, O121, O128, O145, O157 and O174) and nine H-types (H2, H4, H7, H8, H11, H19, H21, H25 and H28). The different primer sets, primer concentrations and their PCR conditions are described in Table S5. For some samples, only the O- (*n*: 12) or H-type (*n*: 3) was determined. In case of discrepant results with the bioinformatics workflow, the presence/absence of the respective O- and/or H-type determining genes was confirmed with conventional PCR, for which primer sequences and concentrations and PCR conditions are provided in Table S6. The serotype information was updated based on these results as shown in Table S7, which contains an overview of the determined serotypes for the tested samples.

### Validation of the bioinformatics workflow

#### Validation strategy

We built upon a previously described validation framework with performance metrics adapted towards our purpose of exhaustively validating the bioinformatics workflow: repeatability, reproducibility, accuracy, precision, sensitivity and specificity [26]. A full overview of all performance metrics and their corresponding definitions and formulas is presented in Table 2. Repeatability and reproducibility of the workflow were evaluated by running the bioinformatics workflow twice on the same dataset on the same and a separate computational environment, respectively. The two computational environments were Python 3.7.5 and Python 3.7.4 on two different Ubuntu 18.04.3 LTS (64-bit) servers. Accuracy, precision, sensitivity and specificity all require classification of workflow results as either true positives (TPs), false positives (FPs), true negatives (TNs) or false negatives (FNs), determined from comparison against a reference that represents the ‘ground truth’ (see Table S8). Two approaches were adopted. Firstly, if information from conventional wet-lab methods was available, this was denoted as ‘database reference’. This corresponds to the box ‘Molecular’ in Fig. 2. Secondly, if this information was not available or impossible to obtain, i.e. for plasmid replicon detection and cgMLST, a ‘tool reference’ was used where the workflow output was compared to results of staple bioinformatics tools widely used within the scientific community. This corresponds to the box ‘*In silico’* in Fig. 2. Only samples that did not have any failures for quality control checks (Table 1) were considered for validation of the bioinformatics workflow (Table S1). The three bioinformatics approaches (blast+, KMA, SRST2) were evaluated for all assays unless stated otherwise. Details for individual bioinformatics assays are provided in the next sections. A schematic overview of the validation strategy is provided in Fig. 2.

**Table 2.** Evaluated performance metrics and their corresponding definitions and formulas

Metric	Definition	Formula	Assay-specific definitions	Bioinformatics assay					
				Genotypic AMR characterization	Virulence gene detection		Serotype determination	Plasmid replicon detection	Sequence typing
					*stx*	other			
Repeatability	Agreement of the assay based on intra-assay replicates	Repeatability=100 %×(# intra-assay replicates in agreement)/(total # intra-assay replicates)	Intra-assay replicate	Running the bioinformatics workflow twice on the same dataset in the same computational environment					
Reproducibility	Agreement of the assay based on inter-assay replicates	Reproducibility=100 %×(# inter-assay replicates in agreement)/(total # inter-assay replicates)	Inter-assay replicate	Running the bioinformatics workflow twice on the same dataset in a separate computational environment					
Accuracy	The likelihood that results of the assay are correct	Accuracy=100 %×(TP+TN)/(TN +FN+TP+FP)	True positive result	Resistance predicted for antibiotic group for which there is phenotypic resistance	Gene detected by workflow and PCR		Same serotype detected as by PCR-based methods	Plasmid replicon detected by workflow and PlasmidFinder tool	Detection of the same allele as the PubMLST.org platform
Precision	The likelihood that detected results of the assay are truly present	Precision=100 %×TP/(TP+FP)	False negative result	Sensitivity predicted for antibiotic group for which there is phenotypic resistance	Gene detected by PCR but not the workflow		Different serotype detected as by PCR-based methods	Plasmid replicon detected by PlasmidFinder tool but not the workflow	Detection of a different allele as the PubMLST.org platform
Sensitivity	The likelihood that a result will be correctly picked up by the assay when present	Sensitivity=100 %×TP/(TP+FN)	True negative result	Sensitivity predicted for antibiotic group for which there is phenotypic sensitivity	Gene not detected by workflow nor by PCR		No serotype detected in negative control sample	Plasmid replicon not detected by workflow nor by PlasmidFinder tool	No detection of an allele in negative control sample
Specificity	The likelihood that a result will not be falsely picked up by the assay when not present	Specificity=100 %×TN/(TN+FP)	False positive result	Resistance predicted for antibiotic group for which there is phenotypic sensitivity	Gene detected by workflow but not by PCR		Serotype detected in a negative control sample	Plasmid replicon detected by workflow but not by PlasmidFinder tool	Detection of an allele in negative control sample

TP, True positive; FP, false positive; FN, false negative; TN, true negative.

#### AMR prediction

AMR prediction came out of genotypic AMR detection and was evaluated by comparing results of phenotypic testing of a database reference with results of our workflow. The validation was therefore performed at the level of phenotypic AMR prediction, even though the workflow only reports AMR at the genotypic level (as discussed above). Because AMR associations are reported by the workflow at the antibiotics group level (beta-lactamases, fluoroquinolones, etc.), the validation was also performed on a per-group basis through similarly combining the phenotypic data per antibiotic group (Table S2). A strain was considered resistant to an antibiotics group if it exhibited (intermediate) phenotypic resistance to at least one of the tested antibiotics of that group. Samples that were not tested phenotypically for AMR were excluded from the validation set. A strain was predicted to be resistant to an antibiotic group if at least one gene or point mutation associated with resistance to the respective antibiotics group was detected. Mutations detected by PointFinder without a PubMed identifier were not considered. The following definitions of classification were used to calculate performance metrics: TP and FN as cases with phenotypic resistance to an antibiotic group where the workflow predicted resistance and sensitivity, respectively, and TN and FP as cases with phenotypic sensitivity to an antibiotic group where the workflow predicted sensitivity and resistance, respectively.

#### Virulence gene detection

Virulence gene detection was evaluated by comparing PCR results for the database reference with results of our workflow. The positive test set for this assay corresponded to all virulence genes detected by PCR, and the negative set to the tested virulence genes for which PCR gave negative results. The following definitions were then used: TP as genes detected by both our workflow and PCR; FN as genes missed by our workflow but reported by PCR; FP as genes detected by our workflow but not reported by PCR; and TN as genes detected by neither our workflow nor PCR. Validation was performed separately for *stx* genes and 20 other virulence genes. For *stx* gene detection, the VirulenceFinder *stx* database was employed. For detection of other virulence genes, since the VirulenceFinder *E. coli* database did not contain all of them, a custom database was used with sequences extracted from the VirulenceFinder *E. coli* database complemented with sequences retrieved from NCBI. The tested genes were: *aaiC, aggR, bfpA, eae, ehxA, ent/espL2, espP, ipaH, katP, lt, nleA, nleB, nleE, nleF, nleH1-2, saa, sth, stp, subA* and *terB*. A full overview and accession numbers are provided in Table S10.

#### Serotype determination

Serotype determination was evaluated by comparing serotypes reported by the workflow with results of PCR-based assays. Samples for which only the O- (*n*: 12) or H-type (*n*: 3) was tested with PCR-based methods were considered matching if the known O- or H-type was predicted correctly by the workflow. Samples with O-types that cannot be distinguished using WGS [14, 65]: EH1873 (O17/O44/O77), EH1389 (O90/O127), EH1641 and EH1766 (O118/O151), and EH1757 (O128ab/O128ac) were omitted from the validation. These O-groups have identical O-antigen coding genes, making them impossible to designate based on the detection of these genes [65]. The positive test set consisted of STEC samples for which the serotype was (partially) determined with PCR-based methods, with TP and FN defined as serotypes where the output of our workflow corresponded, or did not correspond, to the PCR results, respectively. The negative test set consisted of ten negative control samples from species other than *E. coli* (Table S1), for which no serotype should be detected with the workflow, with TN and FP defined as correctly unidentified and falsely identified *E. coli* serotypes, respectively.

#### Plasmid replicon detection

Because no conventional data existed for the presence of plasmid replicons in the validation dataset, validation was performed using a tool reference. The output of plasmid replicon detection by the workflow was compared with the online CGE PlasmidFinder tool 2.1 [13] (https://cge.cbs.dtu.dk/services/PlasmidFinder/) to which assemblies were provided as input selecting the ‘*Enterobacteriaceae’* database. Other settings were left at default values: a minimum percentage identity of 95 % and a minimum target coverage of 60 %. The positive test set for the validation corresponded to all plasmid replicons detected by the online PlasmidFinder tool, with TP defined as plasmid replicons detected by the workflow and the online PlasmidFinder and FN as plasmid replicons detected by the online PlasmidFinder but not the workflow. The negative test corresponded to all plasmid replicons in the database that were not detected (on a per-sample basis), with TN defined as plasmid replicons in the database not detected by the workflow and online PlasmidFinder and FP as plasmid replicons detected by the workflow but not the online PlasmidFinder.

#### Sequence typing

Because no conventional data existed for cgMLST in the validation dataset, validation was performed using a tool reference for which the sequence query tool from PubMLST.org was used because it allows querying against the EnteroBase *E. coli* cgMLST scheme. Assemblies were used as input for PubMLST.org. Performance evaluation for this assay was limited to blast+ and KMA, because SRST2 takes several days to finish for a single sample and is therefore infeasible for routine typing. The positive and negative set corresponded to all loci in the cgMLST scheme typed in the STEC and negative control samples, respectively. The following definitions were used, considering only perfect hits (i.e. full length and perfect identity): TP and FN as alleles of loci where the output of our workflow corresponded, or did not correspond, to the tool reference. TN and FP were evaluated by analysing negative control samples with the sequence typing workflow, with TN and FP defined as correctly unidentified and falsely identified alleles, respectively.

### Benchmarking execution time for the different detection types of the bioinformatics workflow

Execution times for the different detection types (blast+, KMA, SRST2) for the different assays of the bioinformatics workflow were evaluated by running them on ten randomly selected samples (EH1389, EH1823, TIAC1185, TIAC1193, TIAC1245, TIAC1248, TIAC1400, TIAC1478, TIAC1523 and TIAC1947). All analyses were run sequentially using eight threads on a virtualized Ubuntu 18.04.4 LTS server (443 GB RAM, 4 x Intel Xeon E7-4850 CPUs) with solid-state drives where no other analyses were running. All assays were executed in triplicate on each sample to account for execution time variability. For each assay, the entire execution time was measured, including creation of output reports and reformatting of output files. Due to the extremely long execution time of cgMLST with SRST2, those benchmarks were limited to the first 50 loci of the scheme and afterwards linearly extrapolated to estimate the execution time for the full scheme (2513 loci).

## Results

### Evaluation of dataset quality

Out of 137 sequenced samples, five were discarded because their resulting genome assembly size was too small, resulting in various failed QC checks. One additional sample was discarded, because it failed the ‘percent cgMLST genes identified’ check, which was due to an approximately equal mix of two *E. coli* strains with a different serotype. One sample (EH2038) passed all quality checks, but manual investigation revealed a low-level contamination of an *E. coli* with a different serotype (see Fig. S4). Because this sample was not flagged by our QC checks, it was nevertheless included in the performance evaluation. Lastly, in some samples a latent presence of *Bos taurus* was detected, probably originating from the original creation of the genomic DNA that was not targeted and hence not noticed in the original typing by means of conventional methods. Since no impact on the performance evaluation was detected and the remaining samples had a sufficiently high median coverage of 44×, this dataset of 131 samples was used for the validation. See the Supplementary Material for a more extended description of validation dataset quality. An overview of diversity contained within the validation dataset based on cgMLST is presented in Fig. 3, and demonstrates overall large diversity of the dataset including both more closely and more distantly related samples.

### Evaluation of validation dataset

#### AMR prediction

Performance metrics for AMR prediction through genotypic AMR characterization are provided in Table 3. Across 42 human samples that had been phenotypically typed for AMR, a total of 283 observations were available, including 83 resistant and 200 susceptible phenotypes. Out of 83 resistant observations, 79 were correctly identified by all three bioinformatics approaches. The *dfrA1* gene associated with resistance to trimethoprim was missed in sample EH1811 by KMA (due to sequence identity filtering), but was correctly detected by blast+ and SRST2. The remaining three observations were missed (i.e. no genotypic detection of resistance whereas the phenotype indicated resistance) by all detection methods and were linked to the antibiotic group beta-lactamases. For these observations, the phenotypic testing had intermediate results for resistance to ampicillin (samples EH1858 and EH1923) and were therefore classified as resistant to the corresponding group (beta-lactamases). Out of 200 susceptible phenotypic observations, 196 were correctly predicted by all three bioinformatics approaches. The remaining four were FPs detected with all three methods (i.e. genotypic detection of resistance whereas the phenotype indicated susceptibility) and were limited to aminoglycosides (*n*: 2), fluoroquinolones (*n*: 1, caused by a point mutation) and sulphonamides (*n*: 1). All genotypic features detected by the workflow resulting in AMR predictions were evaluated with PCR for genes (Table S15) and Sanger sequencing for point mutations (Table S16), which confirmed that all of them were present in the corresponding samples. The large majority of resistance predictions were based on the presence of genes, and only five point mutations with PubMed identifier were detected across all samples that were all associated with fluoroquinolone resistance, resulting in four TPs and one FP when compared with the phenotype. This resulted in an accuracy, precision, sensitivity and specificity of 97.53, 95.24, 96.39 and 98.00 %, respectively, for both blast+ and SRST2, and 97.17, 95.18, 95.18 and 98.00 % for KMA, respectively. Results for all intra- and inter-assay replicates were always 100 % concordant, resulting in a repeatability and reproducibility of 100 %. This was also the case for all other bioinformatics assays, which are therefore not further discussed in the following sections.

**Table 3.** Performance metrics for the different bioinformatics assays for the blast+-, KMA- and SRST2-based detection methods

Bioinformatics assay		Reference standard	Detection method	TP	FN	TN	FP	Accuracy (%)	Precision (%)	Sensitivity (%)	Specificity (%)	Repeatability (%)	Reproducibility (%)
AMR prediction		Database	blast+	80	3	196	4	97.53	95.24	96.39	98.00	100.00	100.00
			KMA	79	4	196	4	97.17	95.18	95.18	98.00	100.00	100.00
			SRST2	80	3	196	4	97.53	95.24	96.39	98.00	100.00	100.00
Virulence gene detection	*stx* genes	Database	blast+	138	3	121	0	98.85	100.00	97.87	100.00	100.00	100.00
			KMA	141	0	120	1*	99.62	99.30	100.00	99.17	100.00	100.00
			SRST2	141	0	120	1*	99.62	99.30	100.00	99.17	100.00	100.00
	Other genes	Database	blast+	883	20	718	0	98.77	100.00	97.79	100.00	100.00	100.00
			KMA	903	0	717	1	99.94	99.89	100.00	99.86	100.00	100.00
			SRST2	902	1	718	0	99.94	100.00	99.89	100.00	100.00	100.00
Serotype determination		Database	blast+	103	3	10	0	97.41	100.00	97.17	100.00	100.00	100.00
			KMA	104	2	10	0	98.28	100.00	98.11	100.00	100.00	100.00
			SRST2	104	2	10	0	98.28	100.00	98.11	100.00	100.00	100.00
Plasmid replicon detection		Tool	blast+	333 (333)†	0 (15)†	12 505 (12 490)†	0 (0)†	100.00 (99.88)†	100.00 (100.00)†	100.00 (95.69)†	100.00 (100.00)†	100.00	100.00
			KMA	292 (299)†	41 (49)†	12 496 (12 490)†	9 (0)†	99.61 (99.62)†	97.01 (100.00)†	87.69 (85.92)†	99.93 (100.00)†	100.00	100.00
			SRST2	319 (334)†	14 (14)†	12 490 (12 490)†	15 (0)†	99.77 (99.89)†	95.51 (100.00)†	95.80 (95.98†)	99.88 (100.00)†	100.00	100.00
Sequence typing (cgMLST)		Tool	blast+	328 329	874	24 595	535	99.60	99.84	99.73	97.87	100.00	100.00
			KMA	327 387	1816	24 595	535	99.34	99.84	99.45	97.87	100.00	100.00
			SRST2‡	–	–	–	–	–	–	–	–	–	–

*Probably caused by low-level within-species contamination of sample EH2038.

†Updated results when FP results that could be traced back to algorithmic issues with blast+ are considered as TP.

‡cgMLST using SRST2 could not be evaluated due to the long running times.

TP, true positive; FP, false positive; FN, false negative;TN, true negative.

#### Virulence gene detection

Performance metrics were evaluated separately for the *stx* genes (*stx1* and *stx2*) and other virulence factor encoding genes, and are provided in Table 3. Information for *stx1* and *stx2* from conventional methods was available for all 131 samples (Table S4)*,* resulting in a total of 262 observations. For seven samples, no *stx* genes were detected, indicating that these are technically not STEC isolates (but were still retained for the validation). Out of 141 *stx*-positive observations, only three could not be identified with blast+, but all were correctly detected with KMA and SRST2. For blast+, in two cases the gene was present on a contig that had been filtered because it had a kmer-coverage <10, and the other mismatch was caused by contig fragmentation in the *stx* gene sequence causing the gene to be filtered out during gene detection. For the 121 *stx-*negative observations, a single FP was reported by KMA and SRST2 in sample EH2038 that showed signs of contamination with another *E. coli *. Given the low depth of the detected gene, it is likely that the *stx2* locus present in the contaminant was detected, which was missed by blast+ because low-depth contigs were filtered out of the assembly. This resulted in an accuracy, precision, sensitivity and specificity of 98.85, 100, 97.87 and 100 %, respectively, for blast+, and 99.62, 99.30, 100 and 99.17 % for KMA and SRST2.

Performance for the detection of the other virulence genes was similar. For 903 confirmed positive observations, there were 20, zero and one FN observations with blast+, KMA and SRST2, respectively. FN results by blast+ were caused by contig fragmentation (*n*: 18), contig length filtering (*n*: 1), and a gene that was not incorporated into the (unfiltered) assembly (*n*: 1). For SRST2, the *nleE* gene was missed in sample MB4093, which was correctly detected by KMA at very low depths (kmer coverage <5). For the 718 negative observations, only a single FP was reported by KMA, which was due to the *nleH1-2* gene in sample TIAC1893 being detected at a sequence identity of 90.93 %, close to the 90 % cutoff. This resulted in an accuracy, precision, sensitivity and specificity of 98.77, 100, 97.79 and 100 %, respectively, for blast+; 99.94, 99.89, 100 and 99.86 %, respectively, for KMA; and 99.94, 100, 99.89 and 100 %, respectively, for SRST2.

#### Serotype determination

Performance metrics for serotype determination are provided in Table 3. Serotype information was available for 111 STEC samples (Table S7), and in three and 12 cases only the H-type and O-type was determined, respectively. After removing five samples with an undistinguishable serotype (see Material and methods), 106 samples remained that corresponded to the positive test set. The workflow detected the correct serotype for 103, 104 and 104 cases with blast+, KMA and SRST2, respectively. The remaining cases were considered FN (i.e. a mismatch between the workflow and conventional method), and were due to different reasons. For samples EH1533 and EH1846 (both typed as O145:H28 with conventional methods), all methods detected the correct O-type, but blast+ detected both H28 and H46 for the H-types, KMA did not detect an H-type, and SRST2 only detected the correct H-type for sample EH1846. Additionally, blast+ failed to detect the H-type in sample TIAC1881 (typed as O26:H11 with conventional methods), which was correctly identified by SRST2 and KMA. The serotype for sample EH1733 (O25:H40) was correctly identified with blast+ and KMA, but SRST2 detected H27 instead of H40. The negative test set consisted of 10 samples from other species for which no serotype should be detected with the workflow, which was always the case for all detection methods This resulted in an accuracy, precision, sensitivity and specificity of 97.41, 100, 97.17 and 100 %, respectively, for blast+, and 98.28, 100, 98.11 and 100 % for KMA and SRST2.

#### Plasmid replicon detection

Results for the online PlasmidFinder tool for all samples are shown in Table S17, and performance metrics for the plasmid replicon detection assay are provided in Table 3. In total, the online PlasmidFinder tool reported 333 replicons across 131 samples that were used as the positive test set. For blast+, results corresponded perfectly to the output of the tool reference, resulting in 100 % for all performance metrics. For KMA, 41 FNs were detected, limited to plasmid replicons IncFIA (*n*: 28), IncQ1 (*n*: 6), IncFIB (*n*: 3), IncFII (n: 2) and IncI1/IncB/O/K/Z (*n*: 2). All mismatches for IncFIA and IncQ1, and a single mismatch for IncFII and IncI1/IncB/O/K/Z, were caused by an algorithmic difference in the calculation of percentage identity. For blast+, this calculation is limited to the aligned part of the sequences, whereas for KMA the whole sequence is considered, leading to a lower identity value and subsequent filtering of the hit. The remaining FNs with KMA were also caused by differences in the calculation of the percentage identity values, which were affected differently by indels in full-length hits. For SRST2, 14 FNs were detected, limited to plasmid replicons IncFIA (*n*: 9), IncI1/IncB/O/K/Z (*n*: 3) and IncFII (*n*: 2). In all of these cases, the replicon variant selected by SRST2 was different from the replicon variant selected by the blast+-based workflow from the PlasmidFinder tool and contained more mismatches, resulting in a divergence value above the 5 % threshold (see example in Table S18). The negative test set consisted of 12 505 observations, i.e. the plasmid replicons present in the PlasmidFinder database that had not been detected using the online PlasmidFinder tool. The detection with blast+ for the negative test set matched perfectly with the online PlasmidFinder tool. This resulted in perfect values of 100 % for all performance metrics for blast+. For KMA, nine FPs were observed. Eight of these were hits at low depth situated on contigs filtered out during the assembly because their kmer coverage was below 10, and were therefore not detected by the blast+-based detection of both the online PlasmidFinder tool and our workflow. The last case was caused by contig fragmentation in the IncFIB plasmid replicon in sample EH1819, which caused the hit to fail the length filtering applied by the online PlasmidFinder tool and our blast+-based workflow. This resulted in an accuracy, precision, sensitivity and specificity of 99.61, 97.01, 87.69 and 99.93%, respectively, for KMA. SRST2 detected 15 FP hits that were spread across different plasmid replicons, including the same nine FP results from KMA-based detection. Fourteen FPs were caused by hits not detected due to low depth (*n*: 13) and contig fragmentation (*n*: 1), as explained for KMA. The remaining FP (sample TIAC1442, Col440I) was due to two different regions similar to Col440 being found on high-coverage contigs above the 90 % identity threshold of SRST2 corresponding to a blast+ identity of 88 %, because SRST2 does not consider indels for divergence calculation. Such FPs were consequently also not detected by the blast+-based detection of both the online PlasmidFinder tool and our workflow. This resulted in an accuracy, precision, sensitivity and specificity of 99.77, 95.51, 95.80 and 99.88 %, respectively, for SRST2.

However, because this reference standard itself is based on blast+, these performance metrics could be perceived as biased because several FPs detected with KMA/SRST2 were found to be well-supported upon inspection, and could therefore be considered as TPs. Performance was therefore re-evaluated by assigning cases where SRST2 and/or KMA detected a plasmid replicon that was missed by blast+ due to algorithmic limitations (i.e. contig fragmentations and depth filtering) to the positive instead of the negative test set. Updated values for performance metrics are listed in Table 3 in parentheses. This resulted in an accuracy, precision, sensitivity and specificity of 99.88, 100, 95.69 and 100 %, respectively, for blast+, 99.62, 100, 85.92 and 100 % for KMA, and 99.89, 100, 95.98 and 100 % for SRST2.

#### Sequence typing

Results for the online PubMLST tool for all samples have been deposited in Zenodo (10.5281/zenodo.4006065), and performance metrics for the sequence typing assay are provided in Table 3. Read mapping-based detection (SRST2) was not evaluated for this assay (see Materials and methods). For the positive test set, on a total of 329 203 observations (i.e. 2513 cgMLST loci for 131 samples), 874 (0.27 %) and 1816 (0.55 %) observations did not correspond to the output of the online PubMLST.org tool (i.e. FNs) for blast+ and KMA, respectively. The majority of mismatches for blast+ (535/874) were due to the workflow reporting a multi-hit so that no allele is provided whereas the online PubMLST returned multiple hits separately in the output (which can be either the same allele on multiple genomic locations or different alleles). Sample EH1858 was an outlier containing 138 FNs (the median of all *E. coli* samples was six), almost exclusively caused by multi-hits of the same allele on different contigs. Further investigation showed that this sample contained several highly similar regions in separate contigs, resulting in multiple copies of the same allele for the duplicated loci in the assembly, therefore most likely representing an assembly artefact. In 223 other cases, the PubMLST sequence query tool reported multiple alleles, with one of them matching the single allele detected by the workflow. All other FNs were caused by alleles present in the EnteroBase scheme missing from the PubMLST scheme, even though both databases were assessed at the same time and alleles that were added to EnteroBase more recently than the missing ones from PubMLST were available in both. This was verified by running our sequence typing workflow with the scheme obtained through the PubMLST API, for which all mismatches in the results were caused by multi-hits (results not shown). Since all FNs were caused by either a different manner of reporting multi-allelic hits and absent loci in the PubMLST scheme, the number of true FN observations is in fact zero, but 874 FN observations were nevertheless retained to provide a conservative performance estimate. For KMA, the largest category of mismatches (*n*: 854/1816) were single alleles detected by PubMLST that were missed by KMA, for which in 118 cases the correct allele was detected but not as a perfect hit (i.e. imperfect percentage identity and/or percentage coverage). An additional 751 mismatches constituted loci where PubMLST detected more than one allele, whereas KMA can only detect a single allele for each locus. The remaining 211 mismatches were caused by a different allele detected as a perfect hit (*n*: 137), and perfect hits for loci not detected by PubMLST (*n*: 74). The negative test set was composed of 10 samples comprising 25 130 observations from the negative control samples, for which the workflow should not detect any allele (which was also verified by analysing these samples through the PubMLST.org tool). In total, 535 perfect hits were detected in the negative control samples. All perfect hits were limited to two *Salmonella enterica* samples: se_SRR11799638 (*n*: 261) and se_SRR11799644 (*n*: 274), and were identical for both detection methods (blast+ and KMA), but were also detected by the online PubMLST reference tool. This resulted in an accuracy, precision, sensitivity and specificity of 99.60, 99.84, 99.73 and 97.87 %, respectively, for blast, and 99.34, 99.84, 99.45 and 97.87 % for KMA.

### Execution times of the bioinformatics workflow for the three different detection methods

An overview of the benchmarking results is provided in Table 4. *De novo* assembly and sequence typing take up the large majority of the total execution time of the workflow, with all other steps only contributing slightly to the total execution time. Read trimming, *de novo* assembly and the contamination check took 31.76, 847.19 and 77.21 s on average per sample, respectively. Execution time for the gene detection assays showed only very limited variation across different databases and was shortest for KMA (on average 5.08 s), followed by blast+ (on average 5.32 s) and SRST2 (on average 29.58 s). The same trends were observed for serotype determination that is based on the same gene detection workflow, with average execution times of 10.72, 10.09 and 55.63 s per sample for blast+, KMA and SRST2, respectively. Detection of point mutations with PointFinder took 15.44 s per sample on average. The execution times for the sequence typing workflow were extrapolated based on the typing of the first 50 loci, resulting in estimates of 1128.84 (18.81 min), 3798.15 (1.06 h) and 1 006 639.45 (11.65 days) seconds per sample, on average, for blast+, KMA and SRST2, respectively.

**Table 4.** Average execution times for the different bioinformatics assays and detection methods

Workflow step	Database	Detection method	Average duration (s)
Read trimming	–	–	31.76
Assembly	–	–	847.19
Contamination check	–	–	77.21
Gene detection	ResFinder	blast	5.35
		KMA	5.03
		SRST2	27.19
	Virulence genes	blast	5.30
		KMA	5.14
		SRST	36.88
	PlasmidFinder	blast	5.30
		KMA	5.06
		SRST2	24.68
PointFinder	PointFinder	–	15.44
Serotype determination	SeroTypeFinder	blast	10.72
		KMA	10.09
		SRST2	55.63
Sequence typing	cgMLST*	blast	1128.84
		KMA	3798.15
		SRST2	1 006 639.45

Averages were calculated over ten samples analysed in triplicate for each assay, and listed values correspond to the duration of a single analysis for a single sample.

*Sequence typing results are based on extrapolation of execution time for the first 50 loci of the cgMLST scheme (out of 2513).

## Discussion

In this study, we present an updated validation framework to extensively validate a bioinformatics workflow (Fig. 1) for the characterization of STEC isolates using WGS data. STEC was chosen as a case study because it is a common cause of outbreaks, and easily exchanges virulence and AMR genes [66]. The validation strategy was applied to several bioinformatics assays of interest for NRCs/NRLs requiring routine pathogen typing and characterization: AMR prediction, virulence gene detection, serotype determination, plasmid replicon detection and sequence typing. In particular, we performed this validation using a validation dataset of 131 isolates extensively characterized by conventional molecular biology-based wet-lab methods. Moreover, we evaluated the suitability of three detection methods commonly used for WGS isolate analysis within this validation framework, i.e. alignment via blast+, kmer read mapping via KMA, and direct read mapping via SRST2.

The validation was performed by extending a previously described validation framework for the bioinformatics component of WGS workflows [26], to which we made several adaptations (Table 2). Firstly, performance evaluation was limited to samples that passed QC checks (except for the contamination check, see Supplementary Methods). This was motivated by the fact that despite performance and quality metrics being heavily intertwined, they constitute two different aspects. Quality metrics evaluate whether input data are of sufficient quality for further analysis. Performance metrics evaluate whether a bioinformatics workflow is algorithmically capable of analysing data correctly to deliver reliable results. In practice, bioinformatics workflows are built to be relatively robust against fluctuations in input data quality, but will typically lose performance quickly once dataset quality drops below a certain threshold [67, 68]. Here, quality metric thresholds were defined by common values (Table 1) used by the community and adapted during development based on the observed results on internally generated datasets. Secondly, reproducibility refers here to analysing the same sample twice on separate computational environments, in contrast to Bogaerts *et al*. [26] where reproducibility was defined as running the bioinformatics workflow twice on the same sample but using datasets generated in different sequencing runs. This choice was motivated by the latter strategy validating inter-run variability of the sequencing process rather than the bioinformatics component, for which inter-run variability of separate computational environments is a conceptually better approach [69] (ISO 23418). Repeatability and reproducibility were always 100 % for all detection methods and all assays, highlighting that the bioinformatics component is especially resilient against repeated analyses being performed on the same or separate computational environments. Thirdly, negative controls for the sequence typing and serotype determination assays were changed. In the previously described validation framework, these were evaluated by running the assay on the validation samples with a scheme from an unmatched species. Here, we opted for a set of samples from unmatched species also often considered by enforcement laboratories, including other foodborne pathogens such as *Listeria monocytogenes* and *S. enterica *, which we analysed with the same workflow as *E. coli* samples for which any generated result (i.e. detection of an allele or serotype) should be perceived as an FP. This approach fits conceptually better with real-world scenarios where an FP result for serotype and sequence typing can originate from erroneous switching and mislabelling of the input strain in the wet-lab (although our workflow already incorporates a contamination check by using Kraken2).

The validation focused specifically on the application of the strategy to a workflow for characterizing STEC isolates. However, parts of the workflow are also applicable to other species. Bioinformatics assays using species-agnostic databases such as ResFinder for AMR prediction (all bacteria) and/or PlasmidFinder for plasmid replicon detection (* Enterobacteriaceae *) can also be applied to other species. Bioinformatics assays using species-specific databases such as the *E. coli* VirulenceFinder database for virulence gene detection and the *E. coli* Enterobase database for sequence typing would be applicable by swapping the underlying databases for the relevant species under consideration. For serotyping, both the underlying database but also the decision tree depicted in Fig. S1 would need to be adapted. However, in all cases, workflow performance is expected to exhibit comparable performance as documented here for STEC, although in a routine setting the workflow would still need to be revalidated using a validation dataset comprising isolates of the target species to verify performance, which is an especially important requirement for obtaining ISO accreditation. An important consideration for any such validation dataset is that it is extensive enough to be representative for isolates typically expected in a routine setting by including a wide range of diversity. Fig. 3 provides a simplified overview of the validation dataset, showcasing large diversity. It also provides more insight into the epidemiological relationship between samples, taking into account that samples were selected to capture as much of the naturally occurring variation as possible (the collection therefore does not provide an unbiased view on surveillance in Belgium). Since mismatches (i.e. FNs, FPs) and location within the phylogeny (Fig. S5) were randomly distributed across the phylogeny rather than systematic [i.e. not associated with specific (sub)clades, which could indicate performance can deviate from general trends for certain (sub)clades], workflow performance estimates can be considered representative for *E. coli* distantly related to the included validation samples. However, in light of the enormous diversity observed within the *E. coli* species, such an analysis remains provisional rather than conclusive.

The performance of three commonly used detection approaches for the different bioinformatics assays was evaluated: alignment-based detection with blast+, kmer-based detection with KMA and read mapping-based detection with SRST2. These three approaches are widely used for different WGS-based bioinformatics workflows with mapping-based approaches considered to be more sensitive, especially at lower sequencing depths [22, 70–72], and kmer-mapping approaches considered particularly rapid for analysing large datasets and databases with only a minor performance cost compared to read mapping [21, 73]. Recently, Cooper *et al*. investigated the performance of AMR gene detection using various methods (including blast+, SRST2 and KMA) [73]. However, to the best of our knowledge, no systematic evaluation of their performance across different bioinformatics assays has been documented before. Based on earlier research and literature review, an acceptance criterion of >95 % for all performance metrics was set to accept the results of a bioinformatics assay. This threshold was reached for all assays, except for plasmid replicon detection with KMA due to algorithmic artefacts. Over all assays combined, we did not find substantial performance differences between the different detection methods (Table 3), consistent with the results obtained by Cooper *et al*. for AMR gene detection. Plasmid replicon detection was an exception where blast+ outperformed both SRST2 and KMA, although it should be highlighted that the latter two methods were not designed for intergenic regions and that the reference standard also used a blast+-based approach, and this therefore does not constitute a completely appropriate comparison. However, execution time did vary considerably between the different detection methods (Table 4). Although the time estimates provided in Table 4 are highly dependent on the used computational architecture and input data size, they do provide a rough estimate allowing comparison between different detection methods. SRST2 takes approximately five or six times longer to complete for most assays compared to blast+, but its execution time increases substantially as database size increases. Consequently, although the additional time investment for SRST2 compared to blast+ remains at most limited to half a minute for most assays and therefore is negligible, its execution time for cgMLST rises steeply and takes too long to complete in a reasonable timeframe, especially in time-sensitive situations such as outbreak investigations. Consequently, incorporation of read mapping-based detection via SRST2 for cgMLST in the workflow would require algorithmic improvements to accelerate the underlying tool processing time, although this would also require re-validation of the workflow before any tool updates that change algorithmic behaviour can be included. KMA execution times for most assays are very similar to those of blast+, and despite also increasing with database size, remain feasible for cgMLST. Moreover, the execution time for KMA can be further optimized by pre-loading databases into shared memory (which was not done for this benchmarking), and this benchmarking does not take into account that the required assembly step for blast+-based detection also takes considerable time and is not required for SRST2- and KMA-based detection (but was nevertheless performed by default in our workflow as it is part of the QC process). There exist two considerations for our evaluation of execution time. Firstly, only one tool was evaluated for every type of detection method, albeit a staple one endorsed by the scientific community, but different software packages exist for each type of detection method, which consequently can also affect performance and execution time. Secondly, performance was not evaluated as a function of data quality, for instance more sensitive detection with mapping-based approaches as reported at low coverages [22, 70, 71, 73]. Notwithstanding this, for high-quality datasets, overall performance for all three detection methods is very similar and execution time differences are limited, except when database sizes become too large in which case blast+ gains a distinct speed advantage over KMA and especially SRST2. Regardless, excluding cgMLST with SRST2, all execution times for any detection method remain very limited in comparison with the overall WGS workflow where sample preparation, library construction and sequencing take several days.

AMR prediction overall had the lowest performance compared to the other assays, but accuracy, precision, sensitivity and specificity were always ˃95 %. The number of observations was relatively limited, as AMR treatment is generally not advised for STEC infections, and was therefore only evaluated for isolates of human origin. There are several potential explanations for the slightly lower sensitivity compared to other bioinformatics assays, in particular the imperfect genotype-to-phenotype relationship with the performance being much higher if only a genotypic endpoint is considered. Validation of any component of the WGS workflow requires comparison against a reference standard that serves as the ground truth [25]. The preferred reference standard for validating bioinformatics assays is high-quality genotypic information obtained from conventional methods. However, in practice, this information might not be available and be infeasible or even simply impossible to obtain. For this assay, we used a phenotypic reference standard instead, which has some intrinsic limitations. A genomic AMR feature (gene or SNP) that is present but not expressed will be classified as an FP, even though it is genotypically correctly identified. We addressed this issue by confirming the presence of all AMR genes and point mutations detected by the workflow with PCR and Sanger sequencing. In all cases, the presence of the respective genomic features was confirmed, demonstrating that all FPs constituted TPs when considering a genotypic endpoint, thereby effectively increasing the accuracy and even obtaining perfect precision and sensitivity. Systematic confirmation was impossible for FN results where no resistance was predicted but phenotypic resistance was nevertheless observed, because multiple AMR genes exist for every type of antimicrobial used, so that a large number of PCRs would be required to evaluate all of the underlying potential genes or other mechanisms that could have resulted in resistance. Despite these limitations, the bioinformatics workflow was still able to predict antimicrobial resistance with relatively very high performance. Regardless, validating the relationship between genomic features and phenotypic characteristics is out-of-scope for the validation of the bioinformatics assay presented here. Genotype-to-phenotype relationships can vary substantially between pathogens and case studies [30, 74] and even between different antibiotics within the same pathogen [75]. The importance of screening for point mutations was also illustrated in this study. Four point mutations associated with fluoroquinolone resistance were detected, in congruence with the observed phenotype for which no corresponding genes were detected. Validation was at the antibiotic group level but for some groups only a subset of associated antibiotics was tested. For instance, for aminoglycosides, gentamycin, streptomycin and kanamycin were phenotypically tested, but other antibiotics such as tobramycin or amikacin were not tested, potentially increasing FP observations by wrongly classifying results as sensitive to the antibiotics group. This would mainly cause issues for antibiotic groups with more members (e.g. aminoglycosides, beta-lactamases), which was not observed in our results, potentially due to the already relatively small number of FP results (*n*: 4).

Virulence gene detection performance was generally very high, although the performance of blast+-based detection was slightly lower than for mapping based detection with either KMA or SRST2. The results of genotypic PCR-based methods were used as a reference standard, namely for *stx* and other virulence genes, therefore representing a high-quality standard. In contrast to standard PCR-based methods, WGS detects specific gene variants that can have different biological properties [76]. The *stx* gene detection with KMA and SRST2 matched perfectly with PCR-based methods when considering that the one FP was probably caused by low-level within-species contamination of sample EH2038. Mismatches of blast+-based detection could largely be traced back to depth filtering and contig fragmentations. Accurate detection of *stx* is crucial for routine surveillance, as it is the defining feature to distinguish commensal *E. coli* or other *E. coli* pathotypes from STEC. Similar results were observed for the detection of the other virulence genes. No substantial differences were found between individual genes, suggesting high performance for gene detection in general, not limited to this specific set of genes.

The performance of serotype determination was slightly lower than for virulence gene detection, potentially explained by the following factors. Firstly, as it is a composite assay, performance is expected to be lower because a single wrong detection for one of the serotype-determining genes can result in a wrong prediction, even when all other genes were correctly identified. Secondly, O-type-determining genes are typically located in low GC-content regions, for which the yield of the Nextera XT kit, used for the preparation of the libraries in this study, typically drops [72, 77]. Other studies have reported similar results, with lower performance for WGS-based serotyping [73, 78]. Nevertheless, the performance for all metrics for all detection methods was always >95 %, indicating that WGS is a suitable alternative to conventional methods, especially because all antigen coding genes can be screened simultaneously, in contrast to PCR-based methods that are typically limited to the most common serotypes.

Performance of the plasmid replicon detection assay showed the most variation for the different detection methods. Reference information from conventional methods was not available, and therefore the online PlasmidFinder webserver [13] was used as a tool reference instead, for which the performance has been described extensively in the scientific literature. Evaluating performance by means of comparison with high-quality genomic information is preferred compared to the current approach where the output of prediction (i.e. our workflow) is compared against another genomic predictor (i.e. the online PlasmidFinder tool), but was necessitated by the absence and infeasibility to generate such reference information (see Bogaerts *et al*. [26] for more elaborate discussion). However, as this reference standard itself is based on blast+, performance metrics were biased. This was illustrated by some results of SRST2 and KMA being labelled as FP that proved to be TP after investigation because they could be traced back to contig fragmentation or regions of low sequencing depths. Concurrently, SRST2 and KMA missed some plasmid replicons detected by blast+ present in the tool reference, a large fraction of which could be traced back to the IncFIA plasmid replicon (see Supplementary Information). Consequently, the overall performance is impacted with, in particular, the sensitivity dropping to 87.69 and 95.80 % for KMA and SRST2, respectively, although both are underestimated. blast+ performance was perfect, but is biased and overestimated due to the aforementioned reasons. A more realistic estimation can be obtained by considering cases where blast+ detection failed due to algorithmic artefacts as TP results, as indicated with ‘†’ in Table 3. Sensitivity then dropped further to 85.92 % for KMA, but increased to 95.98 % for SRST2. The added value of expressing performance in terms of additional performance metrics compared to simply congruence with a reference is illustrated here, as simply comparing accuracies would provide a distorted image of relatively high performance for all methods whereas evaluation of sensitivity and specificity indicated that some methods suffer from a substantially decreased sensitivity (i.e. ability to correctly detect a plasmid replicon) but otherwise exhibit high specificity (i.e. ability to not incorrectly detect a plasmid replicon).

For sequence typing, the PubMLST sequence query tool [79] was used as a standard, as thousands of tests would need to have been performed to obtain genotypic information for all loci and samples. This limits performance evaluation to a tool reference, as highlighted and discussed previously for plasmid replicon detection. The large majority of FNs were caused by differently handling multi-hits (i.e. multiple alleles or copies of the same allele) between our workflow and the PubMLST reference standard. Duplicated loci are typically filtered out when constructing cgMLST schemes [80], but locus duplication is relatively common for a rapidly evolving species such as *E. coli* [81], rendering exclusion of all potentially affected loci from the scheme impossible. For blast+, all FN mismatches were caused by such multi-hits, or differences between the PubMLST tool and EnteroBase cgMLST database. For KMA, some FN mismatches were found that could not be explained as such. In these cases, the detected allele might be the correct one that was not detected by the blast+-based tool reference (e.g. contig depth filtering or assembly fracture), which is impossible to discern through the limitations of using a tool reference. Although performance for both blast+ and KMA was always >97 % for all performance metrics, such algorithmic influences could nevertheless still affect interpretation of routine results because often thresholds are put on the number of different cgMLST loci to define whether isolates are related or not [82]. Thresholds should therefore ideally also consider the employed detection method and database, as opposed to solely using an arbitrary number of allelic differences. Approximately 10 % of the loci of the cgMLST scheme were detected as perfect hits in the *S. enterica *-negative control samples by both blast+ and KMA. As the same alleles were detected by both detection methods and the PubMLST tool as well, an algorithmic artefact is unlikely to be the cause, and more probably these loci are shared across the (core) genome of these two closely related species [83]. A limitation of our implementation is that novel alleles still need to be submitted by the end user to the underlying curated database (in this case EnteroBase). However, through the automated weekly updates, external database additions are rapidly integrated and can consequently be discovered by our workflow. For this assay, and also in general, the validation was limited to the characterization of single isolates and consequently phylogenetic relationships derived through cgMLST-based phylogenetic inference of multiple isolates (e.g. Fig. 3) were not validated. While this is of particular relevance for foodborne outbreak investigation, this represents an additional layer of complexity and would require an entire study on its own using datasets with known epidemiological relationships [32, 38].

Many other tools and pipelines for characterization of bacterial pathogens based on WGS data exist, both for general bacterial WGS analysis and specific to *E. coli* [78], but have typically not been validated, rendering it difficult to compare performance. Notwithstanding this, a limited set of validation efforts exist, allowing performance comparison for certain assays either specifically for *E. coli* or more generally for bacterial pathogens [2, 25, 26, 30]. For AMR prediction, performance was comparable (accuracy 97.17–97.53 %) with concordances reported by Lindsey *et al*. (97 %) for *E. coli* and Feldgarden *et al*. for several species (98.4 %) based on phenotypic data [2, 30]. Kozyreva *et al*. evaluated AMR prediction for several bacterial pathogens genotypically through comparison with PCR-based methods and reported an accuracy of 100 % [25]. For virulence gene detection, Lindsey *et al*. reported a single mismatch between real-time PCR and a WGS bioinformatics workflow on a total of 103 observations for detection of the *stx1*, *stx2*, *eae* and *ehxA* genes for *E. coli* [2]. We found similar performance (accuracy 98.85–99.94 % depending on the database and detection method). Serotyping was also evaluated by Lindsey *et al*. for *E. coli* through comparison with conventional serotyping, with 94.2 % of composite serotypes predicted correctly [2]. We found a slightly higher accuracy of 97.41 % for blast+, and 98.28 % for SRST2 and KMA, evaluated at the level of the composite serogroup. In contrast to Lindsey *et al*., our reference information was obtained with genotypic testing using PCR-based methods instead of phenotypic testing, potentially explaining the higher performance. For sequence typing, Kozyreva *et al*. reported perfect accuracy between MLST alleles identified with their bioinformatics workflow and *in silico* MLST for several bacterial pathogens [25]. Here, cgMLST was evaluated instead of MLST using a tool reference, and similar high performance (accuracy of 99.60 and 99.34 % for blast+ and KMA, respectively) was observed because all misidentified alleles could be traced back to algorithmic differences between our workflow and the tool reference. Combined, these results demonstrate congruence of our validated STEC pipeline with other validation efforts in the field, showcasing that WGS constitutes an excellent alternative to conventional molecular assays.

Our validation dataset has been made publicly available, including both the raw WGS data and all metadata for conventional molecular biology-based methods and *in silico* analyses, and can serve as a resource for laboratories wanting to validate or benchmark their bioinformatics workflows for characterization of STEC isolates (see Supplementary Information and data availability statement). Although other such datasets exist that include more samples, this is typically limited to only one assay [84]. Datasets with metadata available for a wide diversity of assays, including conventional data for AMR, virulence and serotype, such as presented here, remain scarce [34]. The bioinformatics workflow is provided through an interface in Galaxy, making it easily accessible for non-expert bioinformaticians who do not necessarily have the technical knowledge or specialized infrastructure to perform the analyses, which is also available for non-profit usage and to showcase our implementation at https://galaxy.sciensano.be. The generally high performance of the workflow illustrates that it is well suited for pathogen surveillance in both a public health and clinical setting, but can also be of value for research projects that include STEC isolate WGS data. Such resources, coupled with similar validation efforts for other species and bioinformatics methodologies, demonstrate the benefit and feasibility of switching to WGS-based routine pathogen typing and surveillance and will ultimately be crucial for its successful implementation within applied public health settings.

## Supplementary Data

Supplementary material 1
