# Supplementary material for: Validation strategy of a bioinformatics whole genome sequencing workflow for Shiga toxin-producing Escherichia coli using a reference collection extensively characterized with conventional methods
Source: Microb Genom. 2021 Mar 3;7(3):mgen000531. doi: 10.1099/mgen.0.000531 (PMC8190621; doi:10.1099/mgen.0.000531)
Supplement: Supplementary material 1 [file mgen-7-0531-s001.pdf]

MICROBIOLOGY SOCIETY

Supplementary Materials: Validation strategy of a bioinformatics whole genome sequencing workflow for Shiga Toxin-producing *Escherichia coli* using a reference collection extensively characterized with conventional methods

Supplementary Tables

Table S1

Table S1. Overview of validation dataset samples. This first and second columns list the sample name and species, respectively. The third column indicates whether the sample passed quality control (QC) criteria. The fourth column lists the origin of the *E. coli* samples. The fifth column lists the SRA accession number for each sample. (*) Sample EH2038 passed all QC checks and was included in the validation but showed strong indications of a low-level within species contamination. Abbreviations: Not Available (NA).

Sample	Species	Passes QC	Origin	Accession
EH1227	*E. coli*	TRUE	Human feces	SRR11816066
EH1236	*E. coli*	TRUE	Human feces	SRR11816065
EH1239	*E. coli*	TRUE	Human feces	SRR11816089
EH1260	*E. coli*	TRUE	Human feces	SRR11816063
EH1273	*E. coli*	TRUE	Human feces	SRR11816064
EH1348	*E. coli*	TRUE	Human feces	SRR11816067
EH1380	*E. coli*	TRUE	Human feces	SRR11816069
EH1389	*E. coli*	TRUE	Human feces	SRR11816068
EH1533	*E. coli*	TRUE	Human feces	SRR11816083
EH1624	*E. coli*	TRUE	Human feces	SRR11816087
EH1641	*E. coli*	TRUE	Human feces	SRR11816085
EH1648	*E. coli*	TRUE	Human feces	SRR11815973
EH1667	*E. coli*	TRUE	Human feces	SRR11816091
EH1671	*E. coli*	TRUE	Human feces	SRR11816086
EH1717	*E. coli*	TRUE	Human feces	SRR11816082
EH1733	*E. coli*	TRUE	Human feces	SRR11815971
EH1757	*E. coli*	TRUE	Human feces	SRR11815970
EH1766	*E. coli*	TRUE	Human feces	SRR11816084
EH1771	*E. coli*	TRUE	Human feces	SRR11815969
EH1782	*E. coli*	TRUE	Human feces	SRR11815968
EH1783	*E. coli*	TRUE	Human feces	SRR11816071
EH1785	*E. coli*	TRUE	Human feces	SRR11815967
EH1811	*E. coli*	TRUE	Human feces	SRR11816080

EH1813	*E. coli*	TRUE	Human feces	SRR11815966
EH1815	*E. coli*	TRUE	Human feces	SRR11816079
EH1819	*E. coli*	TRUE	Human feces	SRR11816078
EH1823	*E. coli*	TRUE	Human feces	SRR11816077
EH1829	*E. coli*	TRUE	Human feces	SRR11816076
EH1831	*E. coli*	TRUE	Human feces	SRR11816075
EH1836	*E. coli*	TRUE	Human feces	SRR11815965
EH1839	*E. coli*	TRUE	Human feces	SRR11816074
EH1846	*E. coli*	TRUE	Human feces	SRR11816073
EH1847	*E. coli*	TRUE	Human feces	SRR11816072
EH1858	*E. coli*	TRUE	Human feces	SRR11815964
EH1861	*E. coli*	FALSE	Human feces	SRR11815990
EH1873	*E. coli*	TRUE	Human feces	SRR11815972
EH1882	*E. coli*	TRUE	Human feces	SRR11816100
EH1923	*E. coli*	TRUE	Human feces	SRR11816099
EH1965	*E. coli*	TRUE	Human feces	SRR11816098
EH1979	*E. coli*	TRUE	Human feces	SRR11816088
EH2015	*E. coli*	TRUE	Human feces	SRR11816097
EH2038	*E. coli*	TRUE*	Human feces	SRR11816096
EH925	*E. coli*	TRUE	Human feces	SRR11815974
TIAC1181	*E. coli*	TRUE	Beef carcass	SRR11815993
TIAC1182	*E. coli*	TRUE	Beef carcass	SRR11815992
TIAC1185	*E. coli*	TRUE	Beef carcass	SRR11816056
TIAC1186	*E. coli*	TRUE	Beef carcass	SRR11816045
TIAC1188	*E. coli*	TRUE	Beef carcass	SRR11816034
TIAC1192	*E. coli*	TRUE	Minced beef	SRR11816052
TIAC1193	*E. coli*	TRUE	Minced beef	SRR11816051
TIAC1218	*E. coli*	TRUE	Ground pork	SRR11816023
TIAC1220	*E. coli*	FALSE	Beef carcass	SRR11816012
TIAC1221	*E. coli*	FALSE	Beef carcass	SRR11816001
TIAC1223	*E. coli*	FALSE	Beef carcass	SRR11815986
TIAC1226	*E. coli*	FALSE	Beef carcass	SRR11815975
TIAC1227	*E. coli*	FALSE	Beef carcass	SRR11815991
TIAC1228	*E. coli*	TRUE	Beef carcass	SRR11816092
TIAC1240	*E. coli*	TRUE	Beef carcass	SRR11816081
TIAC1241	*E. coli*	TRUE	Beef carcass	SRR11816070
TIAC1242	*E. coli*	TRUE	Beef carcass	SRR11816062
TIAC1243	*E. coli*	TRUE	Beef carcass	SRR11816061
TIAC1244	*E. coli*	TRUE	Beef carcass	SRR11816060
TIAC1245	*E. coli*	TRUE	Beef carcass	SRR11816059
TIAC1246	*E. coli*	TRUE	Beef carcass	SRR11816058
TIAC1247	*E. coli*	TRUE	Minced beef	SRR11816050
TIAC1248	*E. coli*	TRUE	Beef carcass	SRR11816057
TIAC1354	*E. coli*	TRUE	Beef carcass	SRR11816054

TIAC1356	*E. coli*	TRUE	Beef carcass	SRR11816049
TIAC1369	*E. coli*	TRUE	Beef carcass	SRR11816048
TIAC1372	*E. coli*	TRUE	Beef carcass	SRR11816047
TIAC1382	*E. coli*	TRUE	Beef carcass	SRR11816046
TIAC1398	*E. coli*	TRUE	Beef carcass	SRR11816044
TIAC1399	*E. coli*	TRUE	Beef carcass	SRR11816043
TIAC1400	*E. coli*	TRUE	Beef carcass	SRR11816042
TIAC1402	*E. coli*	TRUE	Beef carcass	SRR11816031
TIAC1408	*E. coli*	TRUE	Raw milk cheese	SRR11816053
TIAC1411	*E. coli*	TRUE	Beef carcass	SRR11816030
TIAC1419	*E. coli*	TRUE	Meat	SRR11816000
TIAC1420	*E. coli*	TRUE	Meat	SRR11815999
TIAC1426	*E. coli*	TRUE	Steak tartare	SRR11816041
TIAC1428	*E. coli*	TRUE	Beef carcass	SRR11816029
TIAC1433	*E. coli*	TRUE	Beef carcass	SRR11816014
TIAC1434	*E. coli*	TRUE	Beef carcass	SRR11816013
TIAC1435	*E. coli*	TRUE	Beef carcass	SRR11816028
TIAC1440	*E. coli*	TRUE	Beef carcass	SRR11816027
TIAC1442	*E. coli*	TRUE	Meat	SRR11815998
TIAC1448	*E. coli*	TRUE	Beef carcass	SRR11816040
TIAC1449	*E. coli*	TRUE	Beef carcass	SRR11816026
TIAC1454	*E. coli*	TRUE	Beef carcass	SRR11816025
TIAC1460	*E. coli*	TRUE	Minced beef	SRR11816039
TIAC1463	*E. coli*	TRUE	Beef carcass	SRR11816024
TIAC1464	*E. coli*	TRUE	Beef carcass	SRR11816022
TIAC1472	*E. coli*	TRUE	Beef carcass	SRR11816021
TIAC1475	*E. coli*	TRUE	Beef carcass	SRR11816020
TIAC1477	*E. coli*	TRUE	Beef carcass	SRR11816019
TIAC1478	*E. coli*	TRUE	Beef carcass	SRR11816018
TIAC1479	*E. coli*	TRUE	Beef carcass	SRR11816017
TIAC1484	*E. coli*	TRUE	Beef carcass	SRR11816011
TIAC1507	*E. coli*	TRUE	Beef carcass	SRR11815997
TIAC1520	*E. coli*	TRUE	Meat	SRR11815996
TIAC1521	*E. coli*	TRUE	Meat	SRR11815995
TIAC1522	*E. coli*	TRUE	Meat	SRR11815994
TIAC1523	*E. coli*	TRUE	Meat	SRR11815989
TIAC1526	*E. coli*	TRUE	Meat	SRR11815988
TIAC1527	*E. coli*	TRUE	Meat	SRR11815987
TIAC1528	*E. coli*	TRUE	Minced beef	SRR11816033
TIAC1544	*E. coli*	TRUE	Minced beef	SRR11816032
TIAC1546	*E. coli*	TRUE	Beef carcass	SRR11815985
TIAC1550	*E. coli*	TRUE	Meat	SRR11815984
TIAC1551	*E. coli*	TRUE	Meat	SRR11815983
TIAC1552	*E. coli*	TRUE	Meat	SRR11815982

TIAC1553	*E. coli*	TRUE	Meat	SRR11815981
TIAC1558	*E. coli*	TRUE	Beef carcass	SRR11816016
TIAC1559	*E. coli*	TRUE	Beef carcass	SRR11816015
TIAC1562	*E. coli*	TRUE	Beef carcass	SRR11815980
TIAC1567	*E. coli*	TRUE	Meat	SRR11815979
TIAC1568	*E. coli*	TRUE	Meat	SRR11815978
TIAC1617	*E. coli*	TRUE	Minced beef	SRR11816038
TIAC1631	*E. coli*	TRUE	Minced beef	SRR11816035
TIAC1641	*E. coli*	TRUE	Minced beef	SRR11816036
TIAC1642	*E. coli*	TRUE	Minced beef	SRR11816037
TIAC1653	*E. coli*	TRUE	Beef carcass	SRR11815977
TIAC1664	*E. coli*	TRUE	Meat	SRR11815976
TIAC1878	*E. coli*	TRUE	Beef carcass	SRR11816010
TIAC1880	*E. coli*	TRUE	Beef carcass	SRR11816009
TIAC1881	*E. coli*	TRUE	Beef carcass	SRR11816008
TIAC1883	*E. coli*	TRUE	Beef carcass	SRR11816007
TIAC1884	*E. coli*	TRUE	Beef carcass	SRR11816006
TIAC1885	*E. coli*	TRUE	Beef carcass	SRR11816005
TIAC1886	*E. coli*	TRUE	Milk	SRR11816004
TIAC1887	*E. coli*	TRUE	Beef carcass	SRR11816003
TIAC1888	*E. coli*	TRUE	Beef carcass	SRR11816002
TIAC1893	*E. coli*	TRUE	Milk	SRR11816055
TIAC1946	*E. coli*	TRUE	Milk product (ice cream)	SRR11816094
TIAC1947	*E. coli*	TRUE	Milk product (ice cream)	SRR11816095
TIAC1951	*E. coli*	TRUE	Beef carcass	SRR11816090
TIAC1953	*E. coli*	TRUE	Minced beef	SRR11816093
cj_SRR11799713	*C. jejuni*	TRUE	NA	SRR11799713
cj_SRR11799714	*C. jejuni*	TRUE	NA	SRR11799714
lm_SRR11790964	*L. monocytogenes*	TRUE	NA	SRR11790964
lm_SRR11798770	*L. monocytogenes*	TRUE	NA	SRR11798770
nm_Z1001	*N. meningitidis*	TRUE	NA	SRR6953924
nm_Z1035	*N. meningitidis*	TRUE	NA	SRR6953925
se_SRR11799638	*S. enterica*	TRUE	NA	SRR11799638
se_SRR11799644	*S. enterica*	TRUE	NA	SRR11799644
ye_SRR10949351	*Y. enterocolitica*	TRUE	NA	SRR10949351
ye_SRR11088743	*Y. enterocolitica*	TRUE	NA	SRR11088743

Table S2

Table S2. Overview of antimicrobial resistance determined by phenotypic testing. The first column lists the name of the sample, the remaining columns list the phenotypically determined resistance against the corresponding antibiotics. Antibiotics are listed below the corresponding antibiotic group listed in the top row. Abbreviations: intermediate (I), sensitive (S), resistant (R).

Group	aminoglycosides			beta-lactamases		fluoroquinolones		phenicols	sulphonamides	tetracyclines	trimethoprims
Antibiotic	gentamycin	kanamycin	streptomycin	ampicillin	cefotaxim	ciprofloxacin	nalidixic acid	chloramphenicol	sulphonamide	tetracycline	trimethoprim
EH1236	S	S	S	S	S	S	S	R	S	S	S
EH1239	S	S	S	S	S	S	S	S	S	S	S
EH1260	I	R	R	S	S	S	R	R	R	R	S
EH1273	S	S	I	S	S	S	S	S	S	S	S
EH1348	S	S	R	S	S	S	S	S	S	S	S
EH1380	S	R	S	S	S	S	S	S	S	R	S
EH1389	S	S	S	S	S	S	S	S	S	R	S
EH1533	S	R	S	S	S	S	S	S	S	R	S
EH1624	S	S	R	R	S	S	S	S	R	R	S
EH1641	R	R	R	R	S	S	S	S	R	R	R
EH1648	S	S	S	S	S	S	S	S	S	S	S
EH1667	S	S	S	S	S	S	S	S	S	S	S
EH1671	S	S	R	R	S	S	S	S	R	S	S
EH1717	S	S	S	S	S	S	S	S	S	S	S
EH1733	S	R	R	R	S	S	R	R	R	R	R
EH1757	S	S	R	S	S	S	S	S	R	S	S
EH1766	S	R	R	R	S	S	R	R	R	R	R
EH1771	S	S	S	S	S	S	S	S	S	S	S
EH1782	S	S	S	S	S	S	S	S	S	S	S
EH1783	S	S	R	S	S	S	S	S	R	R	R
EH1785	S	S	R	R	S	S	S	R	R	R	R
EH1811	S	S	R	R	S	S	R	S	R	R	R
EH1813	S	S	R	R	S	S	S	S	R	S	S
EH1815	S	S	R	S	S	S	S	S	R	S	S
EH1819	S	S	S	S	S	S	S	S	S	S	S
EH1823	S	S	S	S	S	S	S	S	S	S	S

EH1829	S	S	R	R	S	S	S	R	R	R	S
EH1831	S	S	R	S	S	S	S	S	R	S	S
EH1836	S	S	S	S	S	S	S	S	S	S	S
EH1839	S	S	R	S	S	S	S	S	R	S	S
EH1846	S	S	S	S	S	S	S	S	S	S	S
EH1847	S	S	S	S	S	S	S	S	S	R	S
EH1858	S	S	S	I	S	S	S	S	S	S	S
EH1861	S	S	S	S	S	S	S	S	S	S	S
EH1873	S	S	S	S	S	S	S	S	S	S	S
EH1882	S	S	S	S	S	S	S	S	S	S	S
EH1923	S	S	I	I	S	S	S	R	R	S	S
EH1965	S	S	S	S	S	S	S	S	S	U	S
EH1979	R	S	R	R	R	R	S	S	S	U	S
EH2015	S	S	S	S	S	S	S	S	S	S	U
EH2038	S	S	S	S	S	S	S	S	S	S	U

Table S3

Table S3. PCR-based detection of virulence genes. Primers and conditions used for testing presence/absence of virulence genes using PCR. The suffixes 'FP' and 'RP' in the primer name refer to the forward and reverse primer, respectively.

Primer name	Target gene	Sequence 5'-3'	Amplicon size (bp)	Annealing temperature (°C)	Primer concentration (nM)	Reference
stx1&2_FP	*stx1* and *stx2**	TTTGTYACTGTSACRGCWGAAGCYTTACG	128-131	50.0	320	[1]
stx1&2_RP		CCCCAGTTCARWGTRAGRTCMACRTC			600	[1]
stx2_FP	*stx2f*	GTTTCCATGACRACGGACAGCAG	122	50.0	80	[1]
stx2_RP		CTGAACTCCATTAAMKCCAGATATG			400	Barbau-Piednoir, E. et al., unpublished
stx1_FP	*stx1*	GTCACAGTAACAAACCGTAACA	95	50.0	250	[2]
stx1_RP		TCGTTGACTACTTCTTATCTGGA			250	[2]
sth_FP	*sth*	TTCACCTTTCGCTCAGGATG	170	50.0	80	[3]
sth_RP		AGCACCCGGTACAAGCAG			400	[3]
lt_FP	*lt*	TTCCCACCGGATCACCAA	62	50.0	200	[3]
lt_RP		CAACCTTGTGGTGCATGATGA			1000	[3]
bfpA_FP	*bfpA*	AATGGTGCTTGCGCTTGCTGC	117	50.0	400	[4]
bfpA_RP		GCAGACGTTGCGCTCATTAC			400	Barbau-Piednoir, E. et al., unpublished
ipaH_FP	*ipaH*	CCTTTTCCGCGTTCCTTGA	64	50.0	200	[3]
ipaH_RP		CGGAATCCGGAGGTATTGC			1000	[3]
aaiC_FP	*aaiC*	TGGTGACTACTTTGATGGACATTGT	313	50.0	80	[5]
aaiC_RP		GACACTCTCTTCTGSGGTAAACGA			400	[5]
aggR_FP	*aggR*	GCCTAAAGGATGCCCTGATG	70	50.0	200	[6]
aggR_RP		GACCAATTCGGACAACTGCAA			1000	[6]
nleF_FP	*nleF*	TGAGGTGAGAAATGAAAATACTGATG	75	50.0	200	[7]
nleF_RP		ATCCCTRTCMTCTATCGTCAT			1000	Adapted from[7]
espP_FP1	*espP*	GATTACAGCACGCATTCATGGTAT	73	62.5	600	[8]
espP_RP1		TCCAGGCATCCTCAGTGACA			600	[8]
espP_FP2*	*espP*	TAATACGACTCACTATAGGG AAACAGCAGGCACTTGAACG	1869	74.0	250	Adapted[9]
espP_RP2*		GGAAACAGCTATGACCATGG GAGTCGTCAGTCAGTAGAT			250	Adapted from[9]
eae_FP	*eae*	CATTGATCAGGATTTTTCTGGTGATA	102	60.0	80	[8]

eae_RP		CTCATGCGGAAATAGCCGTTA			400	[8]
ehxA_FP	*ehxA*	CGTTAAGGAACAGGAGGTGTCAGTA	142	60.0	80	[8]
ehxA_RP		ATCATGTTTTCCGCCAATGAG			400	[8]
katP_FP	*katP*	GAAGTCATATATCGCCGGTTGAA	73	60.0	80	[8]
katP_RP		GTCATTTCAGGAACGGTGAGATC			400	[8]
saa_FP	*saa*	TGCCGCTGGTATAATTTTTCG	85	60.0	80	[8]
saa_RP		AACGCCTGTTCCATGTTGTG			400	[8]
subA_FP	*subA*	AGTGGCTTCCGCATCGG	88	60.0	80	[10]
subA_RP		ATCATTACCCACTGCCGC			400	[10]
ent/espL2_FP	*ent/espL2*	CCTRGATTATTTTCTGCATTTMA	76	60.0	200	Adapted from[7]
ent/espL2_RP		ACTATTGCCAAGTACGYCACAA			1000	Adapted from[7]
terB_FP	*terB*	GCCAGGTTGGCCGTTTC	82	60.0	80	[6]
terB_RP		CCGTCACTCGATACGGCAAT			400	[6]
nleA_FP	*nleA*	AGATAACYCTAATACTAAATATGCC	137	60.0	200	[7]
nleA_RP		GCCCAACCATTGCRCCGATATGAGG			1000	[7]
nleB_FP	*nleB*	CATRTTGAAGGCTGGAASTTTGT	72	60.0	200	Adapted from[7]
nleB_RP		CGYTACAGGRCGATATGTT			1000	Adapted from[7]
nleE_FP	*nleE*	AGAAGCGTTTGAACCTATTTCCA	83	60.0	80	[7]
nleE_RP		TTGGGCGTTTTCCGGATAT			400	[7]
nleH1-2_FP	*nleH*1-2	ACAAGAGARAGTCATAGTGGWTG	69	60.0	200	[7]
nleH1-2_RP		ATCTCYCCCTTAGGCCATCCCA			1000	Adapted from[7]

* ‘f’ variant not included

Table S4

Table S4. Overview of virulence genes detected with PCR-based methods in validation samples. The first column lists the sample name, the remaining columns list the origin of the sample, and the presence or absence of the corresponding virulence genes. '1' denotes that the gene is present according to PCR-based methods, '0' denotes that the gene is not present according to PCR-based methods. '-1' denotes that a gene was not tested with PCR-based methods. Underlined values in bold were confirmed or updated based on conventional PCR results. *Gene was determined to be present with PCR-based method, but further investigation (due to missed detection with WGS) highlighted that this detection was aspecific, i.e. related to a highly similar gene, which was confirmed through Sanger sequencing. The use of another specific primer set (see Supplementary table S3) resolved this aspecific detection. Primer sequences are listed in Supplementary Table S3.

Sample	Origin	*stx1*	*stx2*	*aaiC*	*aggR*	*bfpA*	*eae*	*ehxA*	*ent espL2*	*espP*	*ipaH*	*katP*	*lt*	*nleA*	*nleB*	*nleE*	*nleF*	*nleH1-2*	*saa*	*sth*	*stp*	*subA*	*terB*
EH1227	Human feces	1	0	-1	-1	-1	1	-1	-1	-1	-1	-1	-1	-1	-1	-1	-1	-1	-1	-1	-1	-1	-1
EH1236	Human feces	0	1	-1	-1	-1	1	-1	-1	-1	-1	-1	-1	-1	1	1	-1	-1	0	-1	-1	-1	-1
EH1239	Human feces	1	1	-1	-1	-1	0	-1	-1	**0**	-1	0	-1	-1	-1	-1	-1	-1	1	-1	-1	-1	-1
EH1260	Human feces	1	0	-1	-1	-1	1	-1	-1	-1	-1	-1	-1	-1	-1	-1	-1	-1	-1	-1	-1	-1	-1
EH1273	Human feces	1	0	-1	-1	-1	1	-1	-1	-1	-1	-1	-1	-1	1	1	-1	-1	0	-1	-1	-1	-1
EH1348	Human feces	0	1	-1	-1	-1	1	-1	-1	-1	-1	-1	-1	-1	-1	-1	-1	-1	-1	-1	-1	-1	-1
EH1380	Human feces	1	0	-1	-1	-1	-1	-1	-1	-1	-1	-1	-1	-1	-1	-1	-1	-1	-1	-1	-1	-1	-1
EH1389	Human feces	1	0	-1	-1	-1	0	-1	0	-1	-1	-1	-1	0	0	0	-1	1	1	-1	-1	-1	1
EH1533	Human feces	0	1	-1	-1	-1	1	1	1	0	-1	0	-1	0	1	1	-1	1	0	-1	-1	0	1
EH1624	Human feces	1	0	0	0	0	0	0	0	1	0	0	0	0	0	0	0	0	1	0	-1	0	**1**
EH1641	Human feces	1	0	-1	-1	-1	1	-1	-1	-1	-1	-1	-1	-1	-1	-1	-1	-1	-1	-1	-1	-1	1
EH1648	Human feces	1	0	-1	-1	-1	0	-1	-1	-1	-1	-1	-1	-1	-1	-1	-1	-1	-1	-1	-1	-1	0
EH1667	Human feces	0	1	-1	-1	-1	-1	-1	-1	-1	-1	-1	-1	-1	-1	-1	-1	-1	-1	-1	-1	-1	-1
EH1671	Human feces	1	1	-1	-1	-1	0	1	0	1	-1	0	-1	0	0	0	-1	0	1	-1	-1	1	0
EH1717	Human feces	1	0	-1	-1	-1	1	1	-1	-1	-1	-1	-1	-1	-1	-1	-1	-1	-1	-1	-1	-1	-1
EH1733	Human feces	0	0	-1	-1	-1	1	0	0	0	-1	0	-1	1	0	0	-1	1	0	-1	-1	0	1

Company Limited by Guarantee l Registered in England No. 1039582 l Registered Office Charles Darwin House, 12 Roger Street, WC1N 2JU, UK
Registered as a Charity: 264017 (England & Wales); SC039250 (Scotland)

EH1757	Human feces	1	1	-1	-1	-1	0	-1	-1	-1	-1	-1	-1	-1	-1	-1	-1	-1	-1	-1	-1	-1	0
EH1766	Human feces	1	0	-1	-1	-1	1	-1	-1	-1	-1	-1	-1	-1	-1	-1	-1	-1	-1	-1	-1	-1	1
EH1771	Human feces	0	1	-1	-1	-1	0	-1	-1	-1	-1	-1	-1	-1	-1	-1	-1	-1	-1	-1	-1	-1	0
EH1782	Human feces	1	0	-1	-1	-1	1	-1	-1	-1	-1	-1	-1	-1	-1	-1	-1	-1	-1	-1	-1	-1	1
EH1783	Human feces	1	0	-1	-1	-1	1	1	-1	-1	-1	-1	-1	-1	-1	-1	-1	-1	-1	-1	-1	-1	-1
EH1785	Human feces	0	1	-1	-1	-1	0	-1	-1	**0**	-1	-1	-1	-1	-1	-1	-1	-1	-1	-1	-1	-1	0
EH1811	Human feces	1	1	-1	-1	-1	1	1	-1	**0***	-1	-1	-1	-1	-1	-1	1	-1	-1	-1	-1	-1	-1
EH1813	Human feces	1	0	-1	-1	-1	1	-1	-1	-1	-1	-1	-1	-1	-1	-1	-1	-1	-1	-1	-1	-1	1
EH1815	Human feces	1	0	-1	-1	-1	1	1	-1	-1	-1	-1	-1	-1	-1	-1	-1	-1	-1	-1	-1	-1	-1
EH1819	Human feces	0	1	-1	-1	-1	1	1	-1	-1	-1	-1	-1	-1	-1	-1	-1	-1	-1	-1	-1	-1	-1
EH1823	Human feces	1	0	-1	-1	-1	1	1	-1	-1	-1	-1	-1	-1	-1	-1	-1	-1	-1	-1	-1	-1	-1
EH1829	Human feces	1	1	-1	-1	-1	1	1	-1	-1	-1	-1	-1	-1	-1	-1	-1	-1	-1	-1	-1	-1	-1
EH1831	Human feces	1	0	-1	-1	-1	1	1	-1	-1	-1	-1	-1	-1	-1	-1	-1	-1	-1	-1	-1	-1	-1
EH1836	Human feces	0	1	-1	-1	-1	1	-1	-1	-1	-1	-1	-1	-1	-1	-1	-1	-1	-1	-1	-1	-1	**0**
EH1839	Human feces	1	0	-1	-1	-1	1	1	-1	-1	-1	-1	-1	-1	-1	-1	-1	-1	-1	-1	-1	-1	-1
EH1846	Human feces	0	1	-1	-1	-1	1	1	-1	-1	-1	-1	-1	-1	-1	-1	-1	-1	-1	-1	-1	-1	-1
EH1847	Human feces	1	0	-1	-1	-1	1	1	-1	-1	-1	-1	-1	-1	-1	-1	-1	-1	-1	-1	-1	-1	-1
EH1858	Human feces	1	1	-1	-1	-1	0	-1	-1	-1	-1	-1	-1	-1	-1	-1	-1	-1	-1	-1	-1	-1	1
EH1861	Human feces	0	1	-1	-1	-1	1	-1	-1	-1	-1	-1	-1	-1	-1	-1	-1	-1	-1	-1	-1	-1	1
EH1873	Human feces	0	0	-1	-1	-1	0	-1	-1	-1	-1	-1	-1	-1	-1	-1	-1	-1	-1	-1	-1	-1	0
EH1882	Human feces	0	1	-1	-1	-1	1	-1	-1	-1	-1	-1	-1	-1	-1	-1	-1	-1	-1	-1	-1	-1	0
EH1923	Human feces	0	1	-1	-1	-1	0	-1	-1	-1	-1	-1	-1	-1	-1	-1	-1	-1	-1	-1	-1	-1	0
EH1965	Human feces	0	1	-1	-1	-1	1	-1	-1	-1	-1	-1	-1	-1	-1	-1	-1	-1	-1	-1	-1	-1	1
EH1979	Human feces	1	0	-1	-1	-1	0	-1	-1	-1	-1	-1	-1	-1	-1	-1	-1	-1	-1	-1	-1	-1	1
EH2015	Human feces	0	1	-1	-1	-1	0	-1	-1	-1	-1	-1	-1	-1	-1	-1	-1	-1	-1	-1	-1	-1	1
EH2038	Human feces	1	0	-1	-1	-1	1	-1	-1	-1	-1	-1	-1	-1	-1	-1	-1	-1	-1	-1	-1	-1	1
EH925	Human feces	0	1	-1	-1	-1	0	-1	-1	-1	-1	-1	-1	-1	-1	-1	-1	-1	1	-1	-1	-1	-1
TIAC1181	Food	0	1	0	0	0	1	1	1	-1	0	1	-1	1	-1	1	0	1	0	0	-1	0	1
TIAC1182	Food	1	1	0	0	0	1	1	1	-1	0	1	-1	1	-1	1	1	1	0	0	-1	0	1
TIAC1185	Food	0	1	0	0	0	1	1	1	-1	0	1	-1	1	-1	1	0	1	0	0	-1	0	1
TIAC1186	Food	1	1	0	0	0	1	1	1	-1	0	1	-1	1	-1	1	1	1	0	0	-1	0	1
TIAC1188	Food	0	1	0	0	0	1	1	1	-1	0	1	-1	1	-1	1	1	1	0	0	-1	0	1
TIAC1192	Food	0	1	0	0	0	1	1	1	-1	0	1	-1	1	-1	1	1	1	0	0	-1	0	1

TIAC1193	Food	0	1	0	0	0	1	1	1	-1	0	1	-1	1	-1	1	1	1	0	0	-1	0	1
TIAC1218	Food	0	1	0	0	0	1	1	1	-1	0	1	-1	**1**	**1**	1	1	1	0	0	-1	0	1
TIAC1220	Food	1	0	0	0	0	1	1	1	-1	0	1	-1	0	0	1	0	1	0	0	-1	0	1
TIAC1221	Food	1	0	0	0	0	1	1	1	-1	0	1	-1	1	1	1	1	1	0	0	-1	0	1
TIAC1223	Food	1	0	0	0	0	1	1	1	-1	0	1	-1	0	0	1	0	1	0	0	-1	0	1
TIAC1226	Food	1	0	0	0	0	1	1	1	-1	0	0	-1	0	0	0	1	0	0	0	-1	0	1
TIAC1227	Food	1	0	0	0	0	1	1	0	-1	0	1	-1	0	0	1	1	1	0	0	-1	0	1
TIAC1228	Food	1	0	0	0	0	1	1	1	-1	0	0	-1	1	1	1	1	1	0	0	-1	0	1
TIAC1240	Food	0	1	0	0	0	1	1	1	-1	0	1	-1	1	1	1	1	1	0	0	-1	0	1
TIAC1241	Food	0	1	0	0	0	1	**1**	1	-1	0	1	-1	**1**	**1**	1	0	1	0	0	-1	0	1
TIAC1242	Food	0	1	0	0	0	1	**1**	1	-1	0	1	-1	**1**	**1**	**1**	0	**1**	0	0	-1	0	1
TIAC1243	Food	0	1	0	0	0	1	**1**	1	-1	0	1	-1	**1**	**1**	**1**	1	**1**	0	0	-1	0	1
TIAC1244	Food	0	1	0	0	0	1	**1**	1	-1	0	1	-1	**1**	**1**	**1**	0	1	0	0	-1	0	1
TIAC1245	Food	0	1	0	0	0	1	1	1	-1	0	1	-1	**1**	**1**	**1**	1	1	0	0	-1	0	1
TIAC1246	Food	0	1	0	0	0	1	1	1	-1	0	1	-1	**1**	**1**	**1**	0	**1**	0	0	-1	0	1
TIAC1247	Food	0	1	0	0	0	1	1	1	-1	0	1	-1	**1**	**1**	**1**	1	**1**	0	0	-1	0	1
TIAC1248	Food	0	1	0	0	0	1	1	1	-1	0	1	-1	**1**	**1**	1	0	1	0	0	-1	0	1
TIAC1354	Food	0	1	0	0	0	1	**1**	1	1	0	**1**	-1	**1**	**1**	**1**	1	**1**	0	0	-1	0	**1**
TIAC1356	Food	0	1	0	0	0	1	**1**	1	1	0	**1**	-1	**1**	**1**	**1**	1	**1**	0	0	-1	0	**1**
TIAC1369	Food	0	1	0	0	0	1	1	1	1	0	1	-1	1	1	1	0	1	0	0	-1	0	1
TIAC1372	Food	0	1	0	0	0	1	**1**	1	1	0	**1**	-1	**1**	**1**	**1**	1	**1**	0	0	-1	0	**1**
TIAC1382	Food	0	1	0	0	0	1	**1**	1	1	0	1	-1	**1**	**1**	**1**	1	1	0	0	-1	0	**1**
TIAC1398	Food	0	1	0	0	**0**	1	1	1	1	0	1	-1	1	1	1	0	1	0	0	-1	0	1
TIAC1399	Food	0	1	0	0	0	1	1	1	1	0	1	-1	1	1	1	0	1	0	0	-1	0	1
TIAC1400	Food	0	1	0	0	0	1	1	1	1	0	1	-1	1	1	1	0	1	0	0	-1	0	1
TIAC1402	Food	0	1	0	0	0	1	1	1	1	0	1	-1	1	1	1	0	1	0	0	-1	0	1
TIAC1408	Food	0	1	0	0	0	1	1	1	1	0	1	-1	1	1	1	1	1	0	0	-1	0	1
TIAC1411	Food	0	1	0	0	0	1	1	1	1	0	1	-1	0	1	1	0	1	0	0	-1	0	1
TIAC1419	Food	0	0	0	0	0	1	1	1	1	0	1	-1	1	1	1	1	1	0	0	-1	0	1
TIAC1420	Food	0	1	0	0	0	1	1	1	1	0	1	-1	1	1	1	1	1	0	0	-1	0	1
TIAC1426	Food	0	1	0	0	0	1	1	1	1	0	1	-1	1	1	1	1	1	0	0	-1	0	1
TIAC1428	Food	0	1	0	0	0	1	1	1	1	0	1	-1	1	1	1	0	1	0	0	-1	0	1
TIAC1433	Food	0	1	0	0	0	1	1	1	-1	0	1	-1	1	1	1	0	1	0	0	-1	0	1

TIAC1434	Food	1	1	0	0	0	1	1	1	-1	0	1	-1	1	1	1	1	1	0	0	-1	0	1
TIAC1435	Food	0	1	0	0	0	1	**1**	1	1	0	**1**	-1	**1**	**1**	**1**	1	**1**	0	0	-1	0	**1**
TIAC1440	Food	0	1	0	0	0	1	**1**	1	1	0	**1**	-1	**1**	**1**	**1**	1	**1**	0	0	-1	0	**1**
TIAC1442	Food	1	1	0	0	0	1	**1**	1	1	0	**1**	-1	**1**	**1**	**1**	1	**1**	0	0	-1	0	**1**
TIAC1448	Food	1	1	0	0	0	1	**1**	1	1	0	**1**	-1	**1**	**1**	**1**	1	**1**	0	0	-1	0	**1**
TIAC1449	Food	0	1	0	0	0	1	**1**	1	1	0	1	-1	**1**	**1**	**1**	1	**1**	0	0	-1	0	1
TIAC1454	Food	0	1	0	0	0	1	1	1	1	0	1	-1	1	1	1	1	1	0	0	-1	0	1
TIAC1460	Food	0	1	0	0	0	1	1	1	**1**	0	1	-1	1	1	1	1	1	0	0	-1	0	1
TIAC1463	Food	0	1	0	0	0	1	**1**	1	1	0	1	-1	1	1	1	0	1	0	0	-1	0	1
TIAC1464	Food	0	1	0	0	0	1	1	1	1	0	1	-1	**1**	**1**	**1**	1	**1**	0	0	-1	0	1
TIAC1472	Food	0	1	0	0	0	1	1	1	1	0	1	-1	1	1	1	1	1	0	0	-1	0	1
TIAC1475	Food	0	1	0	0	0	1	**1**	1	1	0	1	-1	1	1	1	0	1	0	0	-1	0	1
TIAC1477	Food	0	1	0	0	0	1	1	1	1	0	0	-1	1	1	1	1	1	0	0	-1	0	1
TIAC1478	Food	0	1	0	0	0	1	1	1	1	0	1	-1	1	1	1	0	1	0	0	-1	0	1
TIAC1479	Food	0	1	0	0	0	1	**1**	1	1	0	1	-1	**1**	**1**	**1**	0	**1**	0	0	-1	0	**1**
TIAC1484	Food	0	1	0	0	0	1	1	1	-1	0	1	-1	**1**	**1**	1	1	**1**	0	0	-1	0	1
TIAC1507	Food	0	1	0	0	0	1	**1**	1	1	**0**	1	-1	**1**	**1**	**1**	1	**1**	0	0	-1	0	**1**
TIAC1520	Food	0	1	0	0	0	1	**1**	1	1	**0**	1	-1	**1**	**1**	**1**	0	**1**	0	0	-1	0	**1**
TIAC1521	Food	0	1	0	0	0	1	**1**	0	1	**0**	1	-1	**1**	**1**	**1**	1	**1**	0	0	-1	0	**1**
TIAC1522	Food	0	1	0	0	0	1	**1**	0	1	**0**	1	-1	**1**	**1**	**1**	1	**1**	0	0	-1	0	**1**
TIAC1523	Food	0	1	0	0	0	1	**1**	0	1	**0**	1	-1	**1**	**1**	**1**	1	**1**	0	0	-1	0	**1**
TIAC1526	Food	0	1	0	0	0	1	**1**	1	1	**0**	1	-1	**1**	**1**	**1**	1	**1**	0	0	-1	0	0
TIAC1527	Food	0	1	0	0	0	1	1	1	1	0	1	-1	1	1	1	1	1	0	0	-1	0	0
TIAC1528	Food	0	1	0	0	0	1	**1**	1	-1	0	1	-1	1	1	1	0	1	0	0	-1	0	1
TIAC1544	Food	0	1	0	0	0	1	1	1	-1	0	1	-1	1	1	1	1	1	0	0	-1	0	1
TIAC1546	Food	0	1	0	0	0	1	**1**	1	**1**	**0**	1	-1	**1**	-1	**1**	0	**1**	0	0	-1	0	**1**
TIAC1550	Food	0	1	0	0	0	1	**1**	1	1	**0**	1	-1	**1**	-1	**1**	0	**1**	0	0	-1	0	**1**
TIAC1551	Food	0	1	0	0	0	1	**1**	1	1	**0**	1	-1	**1**	-1	**1**	0	**1**	0	0	-1	0	**1**
TIAC1552	Food	0	1	0	0	0	1	**1**	1	1	**0**	1	-1	**1**	-1	**1**	0	**1**	0	0	-1	0	**1**
TIAC1553	Food	0	1	0	0	0	1	**1**	1	1	**0**	1	-1	**1**	-1	**1**	0	**1**	0	0	-1	0	**1**
TIAC1558	Food	0	1	0	0	0	1	1	1	-1	0	1	-1	1	1	**1**	0	1	0	0	-1	0	1
TIAC1559	Food	0	1	0	0	**0**	1	1	1	-1	0	1	-1	1	1	1	0	1	0	0	-1	0	1
TIAC1562	Food	0	1	0	0	0	1	**1**	1	1	**0**	1	-1	**1**	-1	**1**	1	**1**	0	0	-1	0	**1**

TIAC1567	Food	0	1	0	0	0	1	**1**	1	1	**0**	1	-1	**1**	-1	**1**	1	**1**	0	0	-1	0	**1**
TIAC1568	Food	0	1	0	0	0	1	**1**	0	1	**0**	1	-1	**1**	-1	**1**	1	**1**	0	0	-1	0	**1**
TIAC1617	Food	1	1	0	0	0	1	**1**	1	1	**0**	1	-1	**1**	-1	**1**	1	**1**	0	0	-1	0	**1**
TIAC1631	Food	1	1	0	0	0	1	1	1	-1	0	1	-1	1	-1	1	1	1	0	0	-1	0	1
TIAC1641	Food	0	1	0	0	0	1	**1**	1	1	**0**	1	-1	**1**	-1	**1**	1	**1**	0	0	-1	0	**1**
TIAC1642	Food	0	1	0	0	0	1	**1**	1	1	0	1	-1	**1**	-1	**1**	0	**1**	0	0	-1	0	**1**
TIAC1653	Food	1	1	0	0	0	1	**1**	1	1	**0**	1	-1	**1**	-1	**1**	1	**1**	0	0	-1	0	**1**
TIAC1664	Food	0	1	0	0	0	1	**1**	1	1	**0**	1	-1	**1**	-1	**1**	0	**1**	0	0	-1	0	**1**
TIAC1878	Food	1	0	0	0	0	1	1	1	-1	0	1	-1	0	**1**	1	**1**	1	0	0	-1	0	0
TIAC1880	Food	1	0	0	0	0	1	1	1	-1	0	1	-1	1	**1**	1	1	1	0	0	-1	0	1
TIAC1881	Food	0	0	0	0	0	1	0	1	-1	0	0	-1	**1**	**1**	**1**	1	**1**	0	0	-1	0	1
TIAC1883	Food	1	1	0	0	0	1	1	0	-1	0	**0**	-1	**1**	**1**	1	1	1	0	0	-1	0	1
TIAC1884	Food	1	0	0	0	0	1	1	1	-1	0	1	-1	0	1	1	**1**	1	0	0	-1	0	0
TIAC1885	Food	1	0	0	0	0	1	1	1	-1	0	1	-1	0	**1**	1	**1**	1	0	0	-1	0	0
TIAC1886	Food	1	0	0	0	0	1	1	1	-1	0	1	-1	**1**	**1**	**1**	1	**1**	0	0	-1	0	1
TIAC1887	Food	1	1	0	0	0	1	1	1	-1	0	**0**	-1	**1**	**0**	1	1	1	0	0	-1	0	1
TIAC1888	Food	1	1	0	0	0	1	1	1	-1	0	1	-1	**1**	**1**	1	1	**1**	0	0	-1	0	1
TIAC1893	Food	0	1	0	0	0	**1**	1	0	-1	0	0	-1	**1**	0	0	0	0	0	0	-1	0	1
TIAC1946	Food	0	0	-1	-1	-1	-1	-1	-1	-1	-1	-1	-1	-1	-1	-1	-1	-1	-1	-1	-1	-1	-1
TIAC1947	Food	0	0	-1	-1	-1	-1	-1	-1	-1	-1	-1	1	-1	-1	-1	-1	-1	-1	-1	1	-1	-1
TIAC1951	Food	0	1	-1	1	-1	0	-1	-1	-1	0	-1	0	-1	-1	-1	0	-1	-1	-1	-1	-1	-1
TIAC1953	Food	0	0	-1	-1	-1	-1	-1	-1	-1	1	-1	**1**	-1	-1	-1	-1	-1	-1	-1	-1	-1	-1

Table S5

Table S5. PCR-based detection of serotyping genes. This table lists the primers used for the PCR-based detection of the O- and H-type determining genes. The first and second columns lists the primer name and target gene, respectively. The suffixes 'FP' and 'RP' in the primer name refer to the forward and reverse primer, respectively. The third fourth, fifth and sixth columns lists the primer sequence, amplicon size, annealing temperature and primer concentration, respectively. The last column contains the literature references for the primers.

Primer name	Target gene	Sequence 5'-3'	Amplicon size (bp)	Annealing temperature (°C)	Primer concentration (nM)	Reference
O25-wzy_FP	*wzy*-O25	AGAGATCCGTCTTTTATTGTTCGC	230	63.5	500	[11]
O25-wzy_RP		GTTCTGGATACCTAACGCAATACCC			500	
O26-wzx_FP	*wzx*-O26	TTTTATCTGGCGTGCTATCG	248	52.0	200	[12]
O26-wzx_RP		CGGGGTTGCTATAGACTGAA			1000	[12]
O45-wzy_FP	*wzy*-O45	TACGATTTCACAAGCTTCCA	255	52.0	200	[12]
O45-wzy_RP		TGCAATCGCATAAGGAAATA			1000	[12]
O55-wbgN_FP	*wgbN*-O55	TGTAATTCGATGCACCAATTCAG	69	52.0	80	[1]
O55-wbgN_RP		CGCTTCGACGTTCGATACATAA			400	[1]
O90/127-_FP	*wzy*-O90/127	TTCATCTCCGCTGGGAATACA	451	58.0	160	[13]
O90/127-_RP		AATTGGTGACGCTGGAATGA			160	
O91-wzx_FP	*wzx*-O91	CATGCTGCTCATTCTTCTCA	135	52.0	80	[12]
O91-wzx_RP		TGGAGTTTGCAACAAACAAA			400	[12]
O103-wzx_FP	*wzx*-O103	GGGCTTGTATTGTTGTACCG	170	52.0	80	[12]
O103-wzx-RP		AGTGGCAAACAGCCAACTAC			400	[12]
O104-wzx_FP	*wzx*-O104	TGCGGGATTAATATCCTTTG	284	52.0	80	[14]
O104-wzx_RP		ACGCCCTAGAAACCTGACTT			400	[14]

O111-wzx_FP	*wzx*-O111	CAATCCAATTTGCATCTTCA	240	52.0	200	[12]
O111-wzx_RP		ACCGCAAATGCGATAATAAC			1000	[12]
O113-wzx_FP	*wzx*-O113	TGACCTTACTTCCTGCGAAT	246	52.0	200	[12]
O113-wzx_RP		AGCACCACGATAGGATTGAA			1000	[12]
O118-wzy_FP	*wzy*-O118/151	ATATTTGCACGATTTACAGATGT	127	52.0	120	[10]
O118-wzy_RP		AAAATATGAAGCAAAATAACAGCC			600	[10]
O121-wzy_FP	*wzy*-O121	TGGATGGCATTCCTCAGTAT	255	52.0	200	[12]
O121-wzy_RP		AGCAAGCCAAAACACTCAAC			1000	[12]
O128-wzx_FP	*wzx*-O128	TCGATCGTCTTGTTCAGGTT	196	52.0	80	[12]
O128-wzx_RP		GAATGCAATGGGCAATTAAC			400	[12]
O145-wzy_FP	*wzy*-O145	TGTTCCTGTCTGTTGCTTCA	292	52.0	80	[12]
O145-wzy_RP		ATCGCTGAATAAGCACCACT			400	[12]
O157-wzx_FP	*wzx*-O157	ATAATCCAGCCAGCAAAGTG	136	52.0	80	[12]
O157-wzx_RP		GGTGCTGCTCTGACATTTTT			400	[12]
O174-wzy_FP	*wzy*-O174	CGGAAGTCGGACTGCTATTTT	541	58.0	80	[13]
O174-wzy_RP		TATGTGACCTAGCACACCCAA			80	
fliCH2_FP	*fliC*-H2	TTACAGCTACAGTTAAAGGCACTACGA	94	Touchdown 70.0-60.0°C (decrease 1.0°C/cycle for 10 cycles)	80	[15]
fliCH2_RP		CCGGCAACTTTAACGTACAACTT			400	[15]

fliCH4_FP	*fliC*-H4	GATTTCAGCGCGGCGAAACT	150	64.0	500	[16]
fliCH4_RP		GGTTGCAGAATCAACGACCG			500	
fliCH7_FP	*fliC*-H7	CCACGACAGGTCTTTATGATCTGA	96	Touchdown 70.0-60.0°C (decrease 1.0°C/cycle for 10 cycles)	80	[15]
fliCH7_RP		CAACTGTGACTTTATCGCCATTCC			400	[15]
fliCH8_FP	*fliC*-H8	GCTGACAAAGGCTCCATTGAA	61	Touchdown 70.0-60.0°C (decrease 1.0°C/cycle for 10 cycles)	80	[15]
fliCH8_RP		GCTCTGCGCCAGTGTTGTTA			400	[15]
fliCH11_FP	*fliC*-H11	TTTTTGTTAGTGCAGCAGATGGTT	69	Touchdown 70.0-60.0°C (decrease 1.0°C/cycle for 10 cycles)	80	[15]
fliCH11_RP		CAATCCCTGTACCAGCTATGTTTG			400	[15]
fliCH19_FP	*fliC*-H19	AATGACCATCACTTCTGCTGG	80	Touchdown 70.0-60.0°C (decrease 1.0°C/cycle for 10 cycles)	80	[10]
fliCH19_RP		TAATTGAGGTCGCATATGCGGC			400	[10]
fliCH21_FP	*fliC*-H21	TACTAGTGCAACCGTTGCC	102	Touchdown 70.0-60.0°C (decrease 1.0°C/cycle for 10 cycles)	80	[10]
fliCH21_RP		AGATCAGATAGTGTCGCTGC			400	[10]

fliCH25_FP	*fliC*-H25	CACAACATYCTTGATAAAGATGG	80	59.0	300	[17]
fliCH25_RP		AACAGAAGCAGCATAGAAGTC			300	
fliCH28_FP	*fliC*-H28	TGGTAACCTCACTCAAAACAATGCT	81	Touchdown 70.0-60.0°C (decrease 1.0°C/cycle for 10 cycles)	80	[15]
fliCH28_RP		CCTGTTGTATGCCAGTTTTTAGTCA			400	[15]
fliCH28 (O145)_FP	*fliC*-H28 (O145)	AGACTACACCTACAATAAAGCTACAAATGATT	83	64.0	300	[17]
fliCH28 (O145)_RP		ATGGTGCTGTTGTTAATGCTAGCA			300	

Table S6

Table S6. PCR-based verification of serotyping genes. This table lists the primers that were used for re-evaluation of the serotype determining genes in case of discrepant results. Per target gene, the first and second lines list the forward and reverse primer, respectively.

**Target gene**	**Sequence 5'-3'**	**Amplicon size (bp)**	**Annealing temperature (°C)**	**Primer concentration (nM)**	**Reference**
wzy-O17/44/73/77/106	GAGGGGCGGATACATTTGTA	849	62.0	500	[13]
	ATACCACAGCGGGATGAAGTT			500	[13]
wzx-O113	TGACCTTACTTCCTGCGAAT	246	60.5	200	[12]
	AGCACCACGATAGGATTGAA			1000	[12]
fliC-H2	TTACAGCTACAGTTAAAGGCACTACGA	94	63.5	80	[15]
	CCGGCAACTTTAACGTACAACTT			400	[15]
fliC-H18	ACGGAGGCAGATGTTGGTTT	305	64.0	500	[16]
	TGAGTATCAGTGCCTGCGTT			500	[16]
fliC-H28	CTGGCATACAACAGGCACAC	285	62.0	300	[10]
	TCAGCTTTGGTGTAAGCGTC			300	[10]
fliC-H40	GGTGCAGTCAAGGATAAAGA	202	59.0	500	[16]
	CATCAAATGCAGTACCACTC			500	[16]

Table S7

Table S7. Serotypes determined with PCR-based methods for validation samples. The first column lists the sample name. The second and third columns contain the results of the additional verification with conventional PCR that was performed in case of discrepant results with the WGS workflow, '-' indicates that no additional PCR was performed. Primers sequences, concentrations and PCR conditions used-for re-evaluation are listed in Supplementary Table S6. The last column contains the final serotype that was used as the reference information in the validation.

Sample	Result O-type conventional PCR verification	Result H-type conventional PCR verification	Final serotype
EH1227	-	H28	O145:H28
EH1236	-	-	O103:H2
EH1239	-	-	O111:H8
EH1260	-	-	O26:H11
EH1273	-	-	O157:H7
EH1348	-	-	O26:H-
EH1380	-	-	O157:H7
EH1389	-	-	O103:H-
EH1533	-	-	O26:H11
EH1624	-	H28	O145:H28
EH1641	-	-	O111:H8
EH1648	-	-	O103:H-
EH1667	-	-	O157:H7
EH1671	-	-	O157:H7
EH1717	-	-	O157:H7
EH1733	-	-	O157:H7
EH1757	-	-	O157:H7
EH1766	-	-	O157:H7
EH1771	-	-	O157:H7
EH1782	-	-	O157:H7
EH1783	-	-	O157:H7
EH1785	-	-	O157:H7
EH1811	-	-	O157:H7
EH1813	-	-	O157:H7
EH1815	-	-	O157:H7
EH1819	-	-	O157:H7
EH1823	-	-	O157:H7
EH1829	-	-	O157:H7
EH1831	-	-	O145:H28
EH1836	-	-	O26:H11
EH1839	-	-	O145:H28
EH1846	-	-	O26:H11
EH1847	-	-	O111:H8

EH1858	-	-	O26:H11
EH1861	-	-	O157:H7
EH1873	-	-	O157:H7
EH1882	-	-	O157:H7
EH1923	-	-	O157:H7
EH1965	-	-	O157:H7
EH1979	-	-	O157:H7
EH2015	-	-	O157:H7
EH2038	-	-	O157:H7
EH925	-	-	O157:H7
TIAC1181	O174	-	O174:H-
TIAC1182	-	-	O55:H-
TIAC1185	-	-	O-:H-
TIAC1186	-	-	O118:H-
TIAC1188	-	-	O118:H-
TIAC1192	-	-	O-:H-
TIAC1193	O25	H40	O25:H40
TIAC1218	-	-	O128:H-
TIAC1220	-	-	O91:H-
TIAC1221	-	H25	O-:H25
TIAC1223	-	-	O-:H-
TIAC1226	-	-	O-:H-
TIAC1227	-	-	O-:H-
TIAC1228	-	-	O-:H-
TIAC1240	-	-	O-:H-
TIAC1241	O17/43/44/77/106	H18	O17/43/44/77/106:H18
TIAC1242	-	-	O-:H-
TIAC1243	-	H4	O-:H4
TIAC1244	-	-	O121:H19
TIAC1245	-	-	O55:H-
TIAC1246	-	-	O-:H28
TIAC1247	-	-	O-:H-
TIAC1248	-	-	O103:H2
TIAC1354	-	-	O26:H11
TIAC1356	-	-	O26:H11
TIAC1369	-	-	O111:H8
TIAC1372	-	-	O103:H2
TIAC1382	-	-	O103:H2
TIAC1398	-	-	O26:H11
TIAC1399	-	-	O111:H8
TIAC1400	-	-	O157:H7
TIAC1402	-	-	O-:H-
TIAC1408	-	-	O-:H-

TIAC1411	-	-	O-:H-
TIAC1419	-	-	O-:H-
TIAC1420	-	-	O45:H-
TIAC1426	-	-	O-:H-
TIAC1428	-	-	O157:H7
TIAC1433	-	-	O157:H7
TIAC1434	-	-	O157:H7
TIAC1435	-	-	O157:H7
TIAC1440	-	-	O157:H7
TIAC1442	-	-	O157:H7
TIAC1448	-	-	O157:H7
TIAC1449	-	-	O157:H7
TIAC1454	-	-	O157:H7
TIAC1460	-	-	O157:H7
TIAC1463	-	-	O157:H7
TIAC1464	-	-	O157:H21/H7
TIAC1472	-	-	O157:H7
TIAC1475	-	-	O157:H7
TIAC1477	-	-	O157:H7
TIAC1478	-	-	O157:H7
TIAC1479	-	-	O157:H7
TIAC1484	-	-	O-:H-
TIAC1507	-	-	O157:H7
TIAC1520	-	-	O157:H7
TIAC1521	-	-	O157:H7
TIAC1522	O90/127	-	O90/127:H-
TIAC1523	-	-	O157:H7
TIAC1526	-	-	O157:H7
TIAC1527	-	-	O157:H7
TIAC1528	-	-	O157:H7
TIAC1544	-	-	O157:H7
TIAC1546	-	-	O157:H7
TIAC1550	-	-	O157:H7
TIAC1551	-	-	O157:H7
TIAC1552	-	-	O157:H7
TIAC1553	-	-	O157:H7
TIAC1558	-	-	O157:H7
TIAC1559	-	-	O157:H7
TIAC1562	-	-	O157:H7
TIAC1567	-	-	O157:H7
TIAC1568	-	-	O157:H7
TIAC1617	-	-	O157:H7
TIAC1631	-	-	O157:H7

TIAC1641	-	-	O157:H7
TIAC1642	-	-	O157:H7
TIAC1653	-	-	O157:H7
TIAC1664	-	-	O157:H7
TIAC1878	-	-	O157:H7
TIAC1880	-	-	O-:H-
TIAC1881	-	-	O157:H7
TIAC1883	-	-	O157:H7
TIAC1884	-	-	O-:H-
TIAC1885	-	-	O-:H-
TIAC1886	-	-	O-:H-
TIAC1887	-	-	O157:H7
TIAC1888	-	-	O-:H-
TIAC1893	-	-	O157:H7
TIAC1946	-	-	O157:H7
TIAC1947	-	-	O157:H7
TIAC1951	-	-	O157:H7
TIAC1953	-	-	O-:H-

Table S8

Table S8. Confusion matrix. Represents classification of results, where 'Actual' corresponds to the reference standard in the validation and 'Predicted' corresponds the output of the bioinformatics workflow.

		**Actual**	
		**+**	**-**
**Predicted**	**+**	True positive (TP)	False positive (FP)
	**-**	False negative (FN)	True negative (TN)

Table S9

Table S9. Primers and conditions used for PCRs detecting AMR genes. AMR genes that were detected with the WGS workflow were also evaluated with PCR, using the primer sets listed in the table. The suffixes 'FP' and 'RP' in the primer name refer to the forward and reverse primer, respectively. The third fourth, fifth and sixth columns lists the primer sequence, amplicon size, annealing temperature and primer concentration, respectively. The last column contains the literature references for the primers.

Primer name	Gene	Sequence (5'-3')	Product size (bp)	Annealing temperature (°C)	Primer concentration (nM)	Reference
aph(3'')-Ib_FP	*aph(3'')-Ib*	GGTTGCCTGTCAGAGGCGG	750	58	200	[18]
aph(3'')-Ib_RP		GTCAGAGGGTCCAATCGC			200	
aph(6)-Id_4FP	*aph(6)-Id*	GACTCCTGCAATCGTCAAGG	560	58	200	[19]
aph(6)-Id_4RP		GCAATGCGTCTAGGATCGAG			200	
sul2_2FP	*sul2*	TGTGCGGATGAAGTCAGCTCC	626	60	200	[20]
sul2_2RP		AGGGGGCAGATGTGATCGAC			200	
sul3_2FP	*sul3*	GAGCAAGATTTTTGGAATCG	789	54	200	[21]
sul3_2RP		CTAACCTAGGGCTTTGGA			200	
aadA1_3FP	*aadA1*	GTGGATGGCGGCCTGAAGCC	525	64	250	[22]
aadA1_3RP		ATTGCCCAGTCGGCAGCG			250	
sul1_2FP	*sul1*	GTGACGGTGTTCGGCATTCT	780	60	250	[23]
sul1_2RP		TCCGAGAAGGTGATTGCGCT			250	
bla_{TEM}_FP	*bla_{TEM}*	ATGAGTATTCAACATTTCCGTG	841	55	250	[24]
bla_{TEM}_RP		TTACCAATGCTTAATCAGTGAG			250	
tetB_FP	*tetB*	TTGGTTAGGGGCAAGTTTTG	659	58	250	[25]
tetB-RP		GTAATGGGCCAATAACACCG			250	
tetA_FP	*tetA*	GCTACATCCTGCTTGCCTTC	210	58	250	[25]
tetA_RP		CATAGATCGCCGTGAAGAGG			250	
aac(3)-IV_FP	*aac(3)-IV*	GTTACACCGGACCTTGGA	674	58	250	[26]
aac(3)-IV_RP		AACGGCATTGAGCGTCAG			250	
dfrA17_FP	*dfrA17*	GATTTCTGCAGTGTCAGA	384	50	250	[27]
dfrA17_RP		CTCAGGCATTATAGGGAA			250	
catA1_FP	*catA1*	CCACCGTTGATATATCCC	623	54	250	[26]
catA1_RP		CCTGCCACTCATCGCAGT			250	
floR_FP	*floR*	CACGTTGAGCCTCTATAT	868	50	250	[26]
floR_RP		ATGCAGAAGTAGAACGCG			250	
blaOXA-1_FP	*blaOXA-1*	TTTTCTGTTGTTTGGGTTTT	427	54	250	[28]
blaOXA-1_RP		TTTCTTGGCTTTTATGCTTG			250	
tet31_FP	*tet31*	CAATCACGCCCAAAAGAA	564	54	250	[29]

tet31_RP		TGTGCCATCCCAGTTTGT			250	
ant(2")-Ia_FP	*ant(2'')-Ia*	GCTCACGCAACTGGTCCA GA	719	60	250	[30]
ant(2")-Ia_RP		GGCACGCAAGACCTCAACCT			250	
aph(4)-Ia_FP	*aph(4)-Ia*	CTGAACTCACCGCGACGTCT	977	58	250	[30]
aph(4)-Ia_RP		TCCACTATCGGCGAGTACTT			250	
dfrA1_FP	*dfrA1*	GGAGTGCCAAAGGTGAACAGC	376	60	250	[31]
dfrA1_RP		GAGGCGAAGTCTTGGGTAAAAAC			250	
cmlA1_FP	*cmlA1*	AGGAAGCATCGGAACGTTGA	101	60	250	[32]
cmlA1_RP		ACAGACCGAGCACGACTGTTG			250	
aph(3')-Ia_FP	*aph(3')-Ia*	ATGGGCTCGCGATAATGTCG	734	54	250	[33]
aph(3')-Ia_RP		AGAAAAACTCATCGAGCATC			250	
T7-gyrA_FP	*gyrA*	TAATACGACTCACTATAGGGGGTACACCGTCGCGTACTTT	350	57	400	Adapted from [34]
M13-gyrA_RP		GGAAACAGCTATGACCATGCAACGAAATCGACCGTCTCT			400	
T7-parE_FP	*parE*	TAATACGACTCACTATAGGGGTCAACCTTATCCCAACGA	887	59	250	Adapted from [35]
M13-parE_RP		GGAAACAGCTATGACCATGTGCTCAAGTACGCCCTCT			250	

Table S10

Table S10. Overview of custom virulence gene database. The first column lists the gene name. The second and third columns lists the source of the sequence and the corresponding NCBI accession number(s), respectively.

Gene name	Source	Accession number(s)
aaiC	VirulenceFinder – *E. coli*	CP003301
aggR	VirulenceFinder – *E. coli*	55989
bfpA	VirulenceFinder – *E. coli*	AB024946
eae	VirulenceFinder – *E. coli*	AB647560, AB647609, FJ609827, AF116899, AJ303141, AB647400, AJ877229, AY223510, AJ877230, AY696839, AJ705052, AB647369, AB647607, FJ609811, FM872421, AJ876652, AB647460, AJ705051, DQ523610, AF022236, FM180568, AB647391, FJ609822, AB647569, AJ705050, AJ876651, AB647610, GU944692, AF530555, AJ271407, AJ744865, AF449417, FJ609810, FJ609831, AB647440, AJ876649, AB647555, AB647368, FJ609833, AB647573, AF530554, AF071034, FM872418, EF204930, ECU59503
ehxA	VirulenceFinder – *E. coli*	AB011549
ent_espl2	NCBI	AE005174.2
espP	VirulenceFinder – *E. coli*	AB011549
ipaH	NCBI	CP000034
katP	VirulenceFinder – *E. coli*	AB011549
lt (ltcA)	VirulenceFinder – *E. coli*	V00275
nleA	VirulenceFinder – *E. coli*	AP019761.1, NZ_AIFB01000023.1, CP043539.1
nleB	NCBI	AP019761.1
nleE	NCBI	AP019761.1
nleF	NCBI	AP018802.1
nleH1-2	NCBI	CP043539.1, CP028379.1, CP041623.1
saa	VirulenceFinder – *E. coli*	AF399919
sth	NCBI	CP025858.1
stp	VirulenceFinder – *E. coli*	AJ555214
subA	VirulenceFinder – *E. coli*	AF399919
terB	NCBI	AP019708.1

Table S11

Table S11. Overview of read trimming statistics for the validation dataset. The first, second and third columns list the sample name, species, and total number of read pairs, respectively. The fourth, fifth, sixth and seventh columns list the number of read pairs where both reads passed trimming, the number of read pairs where only the forward read passed trimming, the number of read pairs where only the reverse read passed trimming and the number of read pairs where both reads were dropped, respectively.

Sample	Species	Total read pairs	Both surviving	Forward read only surviving	Reverse read only surviving	Both dropped
EH1227	*E. coli*	510346	475918	30415	1390	2623
EH1236	*E. coli*	583703	544108	35257	1502	2836
EH1239	*E. coli*	738158	691068	38271	3914	4905
EH1260	*E. coli*	620731	576416	39429	1521	3365
EH1273	*E. coli*	748018	701370	40753	2344	3551
EH1348	*E. coli*	758723	721960	30690	3065	3008
EH1380	*E. coli*	761267	715724	40187	1998	3358
EH1389	*E. coli*	672251	628009	38666	2229	3347
EH1533	*E. coli*	656761	603402	48280	1416	3663
EH1624	*E. coli*	618394	574225	38523	1882	3764
EH1641	*E. coli*	680573	632249	43502	1574	3248
EH1648	*E. coli*	947999	891427	48629	2704	5239
EH1667	*E. coli*	781936	746753	28679	3107	3397
EH1671	*E. coli*	600353	565184	29997	2187	2985
EH1717	*E. coli*	592852	553077	34845	1564	3366
EH1733	*E. coli*	1156207	1095453	52365	3346	5043
EH1757	*E. coli*	888788	836772	45284	2602	4130
EH1766	*E. coli*	614650	569337	40775	1450	3088
EH1771	*E. coli*	795102	743264	45092	2272	4474
EH1782	*E. coli*	1157265	1098537	49306	4317	5105
EH1783	*E. coli*	758862	712513	40599	2183	3567
EH1785	*E. coli*	814224	755510	52279	2177	4258
EH1811	*E. coli*	696949	648944	43157	1570	3278
EH1813	*E. coli*	846621	795054	45459	2300	3808
EH1815	*E. coli*	621662	584874	31572	1885	3331
EH1819	*E. coli*	757745	712100	40351	1965	3329
EH1823	*E. coli*	600619	564287	29470	3869	2993
EH1829	*E. coli*	821932	771233	44336	2617	3746
EH1831	*E. coli*	699914	658133	36086	2359	3336
EH1836	*E. coli*	794966	747140	40865	2884	4077
EH1839	*E. coli*	712804	653673	53476	1578	4077
EH1846	*E. coli*	681047	632319	43292	1603	3833
EH1847	*E. coli*	669379	618004	46348	1379	3648

EH1858	*E. coli*	954835	882644	65052	2163	4976
EH1861	*E. coli*	817719	759970	51556	1993	4200
EH1873	*E. coli*	950555	881150	62058	2268	5079
EH1882	*E. coli*	765435	715597	43394	2480	3964
EH1923	*E. coli*	544475	503907	35009	2340	3219
EH1965	*E. coli*	898362	837238	49275	6711	5138
EH1979	*E. coli*	873968	816811	47927	3921	5309
EH2015	*E. coli*	900982	834815	59364	2117	4686
EH2038	*E. coli*	655424	598954	50248	1934	4288
EH925	*E. coli*	745992	681230	58822	1600	4340
TIAC1181	*E. coli*	763456	696969	58623	2643	5221
TIAC1182	*E. coli*	609346	551869	50783	2105	4589
TIAC1185	*E. coli*	497744	438393	53847	1155	4349
TIAC1186	*E. coli*	996487	903236	83066	2963	7222
TIAC1188	*E. coli*	856532	765919	82091	2275	6247
TIAC1192	*E. coli*	666052	610842	48383	2377	4450
TIAC1193	*E. coli*	613418	575723	30247	3770	3678
TIAC1218	*E. coli*	774292	703332	62719	2547	5694
TIAC1220	*E. coli*	691883	642479	40673	2752	5979
TIAC1221	*E. coli*	1086334	1009301	63831	5191	8011
TIAC1223	*E. coli*	823806	756082	57458	3260	7006
TIAC1226	*E. coli*	725157	680516	35303	3568	5770
TIAC1227	*E. coli*	847327	799812	35134	4974	7407
TIAC1228	*E. coli*	941444	860672	67171	6239	7362
TIAC1240	*E. coli*	650467	590713	52537	2310	4907
TIAC1241	*E. coli*	432093	360319	61677	5147	4950
TIAC1242	*E. coli*	883499	826361	46688	4903	5547
TIAC1243	*E. coli*	715677	660674	46820	2954	5229
TIAC1244	*E. coli*	701342	648752	44115	3377	5098
TIAC1245	*E. coli*	540111	495152	39175	1748	4036
TIAC1246	*E. coli*	704863	652135	44257	3303	5168
TIAC1247	*E. coli*	680663	638886	34151	3807	3819
TIAC1248	*E. coli*	777009	717897	50473	3225	5414
TIAC1354	*E. coli*	611014	546945	57660	1694	4715
TIAC1356	*E. coli*	527291	490588	30760	2597	3346
TIAC1369	*E. coli*	502000	463457	32652	2425	3466
TIAC1372	*E. coli*	564193	528289	29471	2459	3974
TIAC1382	*E. coli*	532671	494175	33100	1997	3399
TIAC1398	*E. coli*	578430	542164	29943	2932	3391
TIAC1399	*E. coli*	755998	694805	53791	2375	5027
TIAC1400	*E. coli*	1041211	955202	75751	3366	6892
TIAC1402	*E. coli*	652868	602180	42524	3330	4834
TIAC1408	*E. coli*	778097	696301	73803	2151	5842

TIAC1411	*E. coli*	680896	611639	60495	2983	5779
TIAC1419	*E. coli*	640336	597478	38162	1339	3357
TIAC1420	*E. coli*	516176	480602	31178	1626	2770
TIAC1426	*E. coli*	540260	497310	37443	2032	3475
TIAC1428	*E. coli*	667892	601408	57902	2822	5760
TIAC1433	*E. coli*	493961	460291	29308	1619	2743
TIAC1434	*E. coli*	382324	338147	40377	754	3046
TIAC1435	*E. coli*	850846	780566	58145	4784	7351
TIAC1440	*E. coli*	753511	708597	34531	5544	4839
TIAC1442	*E. coli*	827093	758077	62858	1700	4458
TIAC1448	*E. coli*	664040	605275	52047	2175	4543
TIAC1449	*E. coli*	447282	401958	39067	2103	4154
TIAC1454	*E. coli*	646369	588810	49861	2674	5024
TIAC1460	*E. coli*	497994	467542	24195	3101	3156
TIAC1463	*E. coli*	1060628	975413	69867	7305	8043
TIAC1464	*E. coli*	581159	520781	53482	2236	4660
TIAC1472	*E. coli*	611879	548941	55437	2398	5103
TIAC1475	*E. coli*	528886	470767	51660	1834	4625
TIAC1477	*E. coli*	783977	708062	65026	3891	6998
TIAC1478	*E. coli*	568134	524952	35278	3790	4114
TIAC1479	*E. coli*	382431	341009	35959	1726	3737
TIAC1484	*E. coli*	424587	398133	21921	2327	2206
TIAC1507	*E. coli*	657811	611285	42099	1205	3222
TIAC1520	*E. coli*	559708	516407	39264	1134	2903
TIAC1521	*E. coli*	578742	532688	41795	1106	3153
TIAC1522	*E. coli*	461176	425331	32305	987	2553
TIAC1523	*E. coli*	547461	505596	37907	1076	2882
TIAC1526	*E. coli*	574587	525490	44648	1133	3316
TIAC1527	*E. coli*	528396	488806	35955	1016	2619
TIAC1528	*E. coli*	492335	446106	39713	2395	4121
TIAC1544	*E. coli*	526355	482961	35672	3631	4091
TIAC1546	*E. coli*	535473	492376	39525	910	2662
TIAC1550	*E. coli*	613521	580890	28146	1692	2793
TIAC1551	*E. coli*	582923	544453	34979	1011	2480
TIAC1552	*E. coli*	694648	656561	33721	1441	2925
TIAC1553	*E. coli*	529209	485053	40511	858	2787
TIAC1558	*E. coli*	347525	311123	33184	743	2475
TIAC1559	*E. coli*	503525	468003	30705	1253	3564
TIAC1562	*E. coli*	659308	622358	32477	1582	2891
TIAC1567	*E. coli*	601363	565167	32478	1253	2465
TIAC1568	*E. coli*	557189	522816	30953	1066	2354
TIAC1617	*E. coli*	537441	493224	38844	1757	3616
TIAC1631	*E. coli*	528938	479754	41368	2843	4973

TIAC1641	*E. coli*	372611	340442	28166	1373	2630
TIAC1642	*E. coli*	387768	344932	38727	1071	3038
TIAC1653	*E. coli*	608671	565844	38605	1278	2944
TIAC1664	*E. coli*	566003	516864	44531	1192	3416
TIAC1878	*E. coli*	623383	576016	42166	1409	3792
TIAC1880	*E. coli*	595120	556155	34576	1350	3039
TIAC1881	*E. coli*	1587692	1479376	36647	7677	63992
TIAC1883	*E. coli*	598195	564007	24784	2386	7018
TIAC1884	*E. coli*	502521	470786	27961	1221	2553
TIAC1885	*E. coli*	580150	543278	32612	1453	2807
TIAC1886	*E. coli*	631376	593471	33510	1448	2947
TIAC1887	*E. coli*	703426	668289	30208	2093	2836
TIAC1888	*E. coli*	585083	552719	28081	1633	2650
TIAC1893	*E. coli*	755353	683970	63254	2465	5664
TIAC1946	*E. coli*	649341	586752	56826	1555	4208
TIAC1947	*E. coli*	594586	542347	45990	2014	4235
TIAC1951	*E. coli*	627701	575243	46594	1824	4040
TIAC1953	*E. coli*	741315	674262	60287	1874	4892
cj_SRR11799713	*C. jejuni*	236470	231497	3	0	4970
cj_SRR11799714	*C. jejuni*	271703	267666	5	0	4032
lm_SRR11790964	*L. monocytogenes*	1137562	1109001	1	0	28560
lm_SRR11798770	*L. monocytogenes*	1024819	1008167	1	0	16651
nm_Z1001	*N. meningitidis*	366005	362476	18	0	3511
nm_Z1035	*N. meningitidis*	412030	408096	22	0	3912
se_SRR11799638	*S. enterica*	786467	770633	14	0	15820
se_SRR11799644	*S. enterica*	540579	522396	12	0	18171
ye_SRR10949351	*Y. enterocolitica*	953355	923130	10	0	30215
ye_SRR11088743	*Y. enterocolitica*	842106	822366	11	0	19729

Table S12

Table S12. Overview of assembly statistics for the validation dataset. The first and second columns list the sample name and species, respectively. The third, fourth and fifth columns list the N50, number of contigs (after filtering on a kmer coverage of ≥10 and length ≥1000 bases) and total cumulative assembly length, respectively.

Sample	Species	N50	Number of contigs	Total length
EH1227	*E. coli*	115089	177	5657184
EH1236	*E. coli*	192591	126	5253348
EH1239	*E. coli*	344753	56	5106611
EH1260	*E. coli*	103260	194	5752781
EH1273	*E. coli*	116273	134	5411553
EH1348	*E. coli*	210106	113	5385533
EH1380	*E. coli*	115097	161	5618952
EH1389	*E. coli*	156633	72	5311241
EH1533	*E. coli*	148067	128	5510819
EH1624	*E. coli*	250771	66	5146893
EH1641	*E. coli*	105048	169	5579208
EH1648	*E. coli*	263613	110	5394558
EH1667	*E. coli*	91489	137	5327808
EH1671	*E. coli*	239937	65	5038526
EH1717	*E. coli*	136706	109	5282171
EH1733	*E. coli*	160353	106	5279968
EH1757	*E. coli*	140830	120	5639701
EH1766	*E. coli*	89301	191	5589007
EH1771	*E. coli*	100555	142	5354769
EH1782	*E. coli*	109629	107	5160306
EH1783	*E. coli*	184280	121	5380400
EH1785	*E. coli*	185637	74	5117137
EH1811	*E. coli*	108228	158	5491256
EH1813	*E. coli*	97480	153	5203452
EH1815	*E. coli*	118052	173	5478369
EH1819	*E. coli*	156978	125	5396860
EH1823	*E. coli*	124308	180	5409798
EH1829	*E. coli*	187855	105	5342525
EH1831	*E. coli*	229653	116	5292039
EH1836	*E. coli*	284599	69	4927160
EH1839	*E. coli*	114262	176	5387307
EH1846	*E. coli*	194744	123	5252914
EH1847	*E. coli*	105287	146	5341319
EH1858	*E. coli*	282935	106	5808747
EH1861	*E. coli*	12689	1204	8639924
EH1873	*E. coli*	299695	36	4941813

EH1882	*E. coli*	248941	64	4926214
EH1923	*E. coli*	144984	108	5329363
EH1965	*E. coli*	134860	138	5242722
EH1979	*E. coli*	144368	93	5154906
EH2015	*E. coli*	160417	126	5445194
EH2038	*E. coli*	115660	122	5202054
EH925	*E. coli*	320378	40	4908160
TIAC1181	*E. coli*	172177	127	5326515
TIAC1182	*E. coli*	151020	133	5353786
TIAC1185	*E. coli*	205777	120	5297789
TIAC1186	*E. coli*	168801	131	5353603
TIAC1188	*E. coli*	187956	134	5302247
TIAC1192	*E. coli*	269174	122	5321413
TIAC1193	*E. coli*	246116	109	5312824
TIAC1218	*E. coli*	189294	139	5424061
TIAC1220	*E. coli*	2703	32	77297
TIAC1221	*E. coli*	2865	33	73906
TIAC1223	*E. coli*	2435	30	69331
TIAC1226	*E. coli*	3016	33	77310
TIAC1227	*E. coli*	1976	36	69013
TIAC1228	*E. coli*	114681	188	5576167
TIAC1240	*E. coli*	159004	119	5306564
TIAC1241	*E. coli*	145618	153	5269762
TIAC1242	*E. coli*	205777	124	5305189
TIAC1243	*E. coli*	248645	118	5343937
TIAC1244	*E. coli*	184473	135	5340864
TIAC1245	*E. coli*	184501	110	5322510
TIAC1246	*E. coli*	184472	133	5362721
TIAC1247	*E. coli*	182925	147	5317888
TIAC1248	*E. coli*	241370	122	5326780
TIAC1354	*E. coli*	205763	119	5340414
TIAC1356	*E. coli*	200619	119	5384962
TIAC1369	*E. coli*	188380	106	5315917
TIAC1372	*E. coli*	157480	119	5342286
TIAC1382	*E. coli*	167260	110	5228826
TIAC1398	*E. coli*	148894	125	5351844
TIAC1399	*E. coli*	146807	115	5344817
TIAC1400	*E. coli*	160593	106	5346299
TIAC1402	*E. coli*	207716	121	5248079
TIAC1408	*E. coli*	188565	117	5296221
TIAC1411	*E. coli*	150086	116	5259417
TIAC1419	*E. coli*	205777	125	5307686
TIAC1420	*E. coli*	148372	119	5290133

TIAC1426	*E. coli*	205777	112	5343840
TIAC1428	*E. coli*	205777	112	5351970
TIAC1433	*E. coli*	184471	120	5425134
TIAC1434	*E. coli*	112596	147	5258823
TIAC1435	*E. coli*	170941	92	5291167
TIAC1440	*E. coli*	148488	131	5331865
TIAC1442	*E. coli*	172405	125	5368285
TIAC1448	*E. coli*	171787	106	5282681
TIAC1449	*E. coli*	148495	115	5342541
TIAC1454	*E. coli*	179675	122	5568684
TIAC1460	*E. coli*	205777	118	5340636
TIAC1463	*E. coli*	187802	113	5346078
TIAC1464	*E. coli*	169030	113	5342808
TIAC1472	*E. coli*	182286	129	5345204
TIAC1475	*E. coli*	215633	117	5311355
TIAC1477	*E. coli*	142690	120	5357549
TIAC1478	*E. coli*	148495	109	5321689
TIAC1479	*E. coli*	184162	113	5325127
TIAC1484	*E. coli*	183182	121	5285258
TIAC1507	*E. coli*	181106	112	5316499
TIAC1520	*E. coli*	168980	128	5352884
TIAC1521	*E. coli*	122284	157	5377600
TIAC1522	*E. coli*	111854	155	5385562
TIAC1523	*E. coli*	148366	125	5392994
TIAC1526	*E. coli*	116865	153	5249810
TIAC1527	*E. coli*	193431	97	5264033
TIAC1528	*E. coli*	188786	131	5292055
TIAC1544	*E. coli*	238327	130	5346330
TIAC1546	*E. coli*	160591	114	5336117
TIAC1550	*E. coli*	122334	141	5302985
TIAC1551	*E. coli*	111606	145	5305782
TIAC1552	*E. coli*	160591	115	5342119
TIAC1553	*E. coli*	184471	113	5341828
TIAC1558	*E. coli*	183744	119	5301580
TIAC1559	*E. coli*	184164	115	5311652
TIAC1562	*E. coli*	146782	129	5422179
TIAC1567	*E. coli*	182924	126	5325026
TIAC1568	*E. coli*	115053	153	5325170
TIAC1617	*E. coli*	172399	124	5350973
TIAC1631	*E. coli*	167879	126	5337312
TIAC1641	*E. coli*	205763	137	5375053
TIAC1642	*E. coli*	185943	113	5307524
TIAC1653	*E. coli*	190123	114	5348408

TIAC1664	*E. coli*	185920	98	5323803
TIAC1878	*E. coli*	119500	130	5232283
TIAC1880	*E. coli*	114262	184	5483026
TIAC1881	*E. coli*	74099	240	5903385
TIAC1883	*E. coli*	104319	181	5328305
TIAC1884	*E. coli*	117809	141	5236506
TIAC1885	*E. coli*	165614	131	5247335
TIAC1886	*E. coli*	103270	219	5475862
TIAC1887	*E. coli*	105392	162	5418395
TIAC1888	*E. coli*	188787	124	5340532
TIAC1893	*E. coli*	102520	140	5315371
TIAC1946	*E. coli*	136862	90	4968279
TIAC1947	*E. coli*	100178	112	5103691
TIAC1951	*E. coli*	120419	106	5323176
TIAC1953	*E. coli*	73317	162	5037614
cj_SRR11799713	*C. jejuni*	56310	98	1788597
cj_SRR11799714	*C. jejuni*	73670	53	1709213
lm_SRR11790964	*L. monocytogenes*	475478	17	2868808
lm_SRR11798770	*L. monocytogenes*	1432376	14	2838303
nm_Z1001	*N. meningitidis*	18473	179	2080124
nm_Z1035	*N. meningitidis*	17322	196	2105933
se_SRR11799638	*S. enterica*	73635	113	4656783
se_SRR11799644	*S. enterica*	58529	156	4655641
ye_SRR10949351	*Y. enterocolitica*	71690	130	4545798
ye_SRR11088743	*Y. enterocolitica*	43036	173	4536597

Table S13

Table S13. Overview of quality control statistics for the validation dataset. The first and second columns list the sample and species. The remaining columns lists the value and the status for the quality metrics, for which an overview is provided in Table 1 (main manuscript). Abbreviations used: Status ('Stat.').

Sample	Species	Contaminants		Median coverage against assembly		% cgMLST genes identified		Average read quality (forward)		Average read quality (reverse)		GC content deviation (forward)		GC content deviation (reverse)		N-fraction (%) (forward)		N-fraction (%) (reverse)		Per base sequence content (forward)		Per base sequence content (reverse)		Minimum read length (forward)		Minimum read length (reverse)	
		Value	Stat.	Value	Stat.	Value	Stat.	Value	Stat.	Value	Stat.	Value	Stat.	Value	Stat.	Value	Stat.	Value	Stat.	Value	Stat.	Value	Stat.	Value	Stat.	Value	Stat.
EH1227	*E. coli*	0.00%	OK	32.00x	OK	99.44%	OK	37	OK	36	OK	0.50%	OK	0.50%	OK	0.01	OK	0	OK	0.26%	OK	0.57%	OK	100.00%	OK	94.42%	OK
EH1236	*E. coli*	0.00%	OK	43.00x	OK	99.40%	OK	37	OK	36	OK	0.50%	OK	0.50%	OK	0.01	OK	0	OK	0.18%	OK	0.44%	OK	100.00%	OK	94.42%	OK
EH1239	*E. coli*	0.00%	OK	59.00x	OK	98.29%	OK	36	OK	36	OK	0.50%	OK	0.50%	OK	0.02	OK	0	OK	0.20%	OK	0.29%	OK	100.00%	OK	100.00%	OK
EH1260	*E. coli*	0.00%	OK	39.00x	OK	98.33%	OK	37	OK	36	OK	0.50%	OK	0.50%	OK	0.01	OK	0	OK	0.17%	OK	0.54%	OK	100.00%	OK	94.42%	OK
EH1273	*E. coli*	0.00%	OK	53.00x	OK	99.36%	OK	37	OK	36	OK	0.50%	OK	0.50%	OK	0.01	OK	0	OK	0.19%	OK	0.63%	OK	100.00%	OK	92.43%	OK
EH1348	*E. coli*	0.00%	OK	56.00x	OK	99.80%	OK	37	OK	36	OK	0.50%	OK	0.50%	OK	0.01	OK	0	OK	0.20%	OK	0.39%	OK	100.00%	OK	94.42%	OK
EH1380	*E. coli*	0.00%	OK	52.00x	OK	99.52%	OK	37	OK	36	OK	0.50%	OK	0.50%	OK	0.01	OK	0	OK	0.18%	OK	0.54%	OK	100.00%	OK	92.43%	OK
EH1389	*E. coli*	0.00%	OK	40.00x	OK	99.44%	OK	36	OK	36	OK	0.50%	OK	0.50%	OK	0.01	OK	0	OK	0.22%	OK	0.57%	OK	100.00%	OK	94.42%	OK
EH1533	*E. coli*	0.00%	OK	49.00x	OK	99.68%	OK	37	OK	36	OK	0.50%	OK	0.50%	OK	0.01	OK	0	OK	0.27%	OK	0.69%	OK	100.00%	OK	92.43%	OK
EH1624	*E. coli*	0.00%	OK	49.00x	OK	99.44%	OK	37	OK	36	OK	0.50%	OK	0.50%	OK	0.01	OK	0	OK	0.21%	OK	0.55%	OK	100.00%	OK	94.42%	OK
EH1641	*E. coli*	0.00%	OK	48.00x	OK	99.12%	OK	37	OK	36	OK	0.50%	OK	0.50%	OK	0.01	OK	0	OK	0.16%	OK	0.53%	OK	100.00%	OK	92.43%	OK
EH1648	*E. coli*	1.57%	Warn	64.00x	OK	97.73%	OK	37	OK	36	OK	0.50%	OK	0.50%	OK	0	OK	0	OK	0.18%	OK	0.36%	OK	100.00%	OK	98.41%	OK

EH1667	*E. coli*	0.00%	OK	61.00x	OK	99.72%	OK	36	OK	36	OK	0.50%	OK	0.50%	OK	0	OK	0	OK	0.22%	OK	0.35%	OK	100.00%	OK	100.00%	OK
EH1671	*E. coli*	0.00%	OK	49.00x	OK	95.82%	OK	37	OK	36	OK	0.50%	OK	0.50%	OK	0.01	OK	0	OK	0.24%	OK	0.50%	OK	100.00%	OK	96.41%	OK
EH1717	*E. coli*	0.00%	OK	44.00x	OK	98.93%	OK	37	OK	36	OK	0.50%	OK	0.50%	OK	0.01	OK	0	OK	0.19%	OK	0.53%	OK	100.00%	OK	92.43%	OK
EH1733	*E. coli*	0.00%	OK	86.00x	OK	99.20%	OK	37	OK	36	OK	0.50%	OK	0.50%	OK	0	OK	0	OK	0.16%	OK	0.42%	OK	98.41%	OK	92.43%	OK
EH1757	*E. coli*	0.00%	OK	62.00x	OK	95.98%	OK	37	OK	36	OK	0.50%	OK	0.50%	OK	0	OK	0	OK	0.21%	OK	0.35%	OK	100.00%	OK	96.41%	OK
EH1766	*E. coli*	0.00%	OK	44.00x	OK	99.72%	OK	37	OK	36	OK	0.50%	OK	0.50%	OK	0.01	OK	0	OK	0.16%	OK	0.52%	OK	100.00%	OK	94.42%	OK
EH1771	*E. coli*	1.72%	Warn	50.00x	OK	99.01%	OK	37	OK	36	OK	0.50%	OK	0.50%	OK	0	OK	0	OK	0.32%	OK	0.40%	OK	100.00%	OK	96.41%	OK
EH1782	*E. coli*	0.00%	OK	91.00x	OK	99.76%	OK	37	OK	36	OK	0.50%	OK	0.50%	OK	0	OK	0	OK	0.20%	OK	0.34%	OK	100.00%	OK	96.41%	OK
EH1783	*E. coli*	0.00%	OK	58.00x	OK	99.84%	OK	37	OK	36	OK	0.50%	OK	0.50%	OK	0.01	OK	0	OK	0.21%	OK	0.66%	OK	100.00%	OK	94.42%	OK
EH1785	*E. coli*	0.00%	OK	67.00x	OK	99.24%	OK	37	OK	36	OK	0.50%	OK	0.50%	OK	0	OK	0	OK	0.22%	OK	0.25%	OK	100.00%	OK	100.00%	OK
EH1811	*E. coli*	0.00%	OK	52.00x	OK	99.20%	OK	37	OK	36	OK	0.50%	OK	0.50%	OK	0.01	OK	0	OK	0.24%	OK	0.60%	OK	100.00%	OK	92.43%	OK
EH1813	*E. coli*	0.00%	OK	60.00x	OK	98.45%	OK	37	OK	36	OK	0.50%	OK	0.50%	OK	0	OK	0	OK	0.25%	OK	0.33%	OK	100.00%	OK	98.41%	OK
EH1815	*E. coli*	0.00%	OK	43.00x	OK	99.56%	OK	37	OK	36	OK	0.50%	OK	1.50%	OK	0.01	OK	0	OK	0.21%	OK	0.62%	OK	100.00%	OK	94.42%	OK
EH1819	*E. coli*	0.00%	OK	57.00x	OK	99.80%	OK	37	OK	36	OK	0.50%	OK	0.50%	OK	0.01	OK	0	OK	0.23%	OK	0.56%	OK	100.00%	OK	94.42%	OK
EH1823	*E. coli*	0.00%	OK	42.00x	OK	99.32%	OK	36	OK	35	OK	0.50%	OK	0.50%	OK	0.01	OK	0	OK	0.34%	OK	0.85%	OK	100.00%	OK	88.45%	OK
EH1829	*E. coli*	0.00%	OK	63.00x	OK	99.28%	OK	37	OK	36	OK	0.50%	OK	0.50%	OK	0.01	OK	0	OK	0.21%	OK	0.58%	OK	100.00%	OK	92.43%	OK
EH1831	*E. coli*	1.42%	Warn	53.00x	OK	99.12%	OK	37	OK	36	OK	0.50%	OK	0.50%	OK	0.01	OK	0	OK	0.23%	OK	0.50%	OK	100.00%	OK	94.42%	OK
EH1836	*E. coli*	1.23%	Warn	65.00x	OK	99.56%	OK	36	OK	36	OK	0.50%	OK	0.50%	OK	0	OK	0	OK	0.29%	OK	0.31%	OK	100.00%	OK	98.41%	OK
EH1839	*E. coli*	0.00%	OK	51.00x	OK	99.48%	OK	37	OK	36	OK	0.50%	OK	0.50%	OK	0.01	OK	0	OK	0.18%	OK	0.70%	OK	100.00%	OK	92.43%	OK

EH1846	*E. coli*	0.00%	OK	51.00x	OK	99.76%	OK	37	OK	36	OK	0.50%	OK	0.50%	OK	0.01	OK	0	OK	0.19%	OK	0.49%	OK	100.00%	OK	92.43%	OK
EH1847	*E. coli*	0.00%	OK	50.00x	OK	99.52%	OK	37	OK	36	OK	0.50%	OK	0.50%	OK	0.01	OK	0	OK	0.25%	OK	0.56%	OK	100.00%	OK	92.43%	OK
EH1858	*E. coli*	0.00%	OK	69.00x	OK	93.83%	Warn	37	OK	36	OK	0.50%	OK	0.50%	OK	0	OK	0	OK	0.21%	OK	0.40%	OK	100.00%	OK	98.41%	OK
EH1861	*E. coli*	0.00%	OK	34.00x	OK	34.22%	Fail	37	OK	36	OK	0.50%	OK	0.50%	OK	0	OK	0	OK	0.28%	OK	0.40%	OK	100.00%	OK	98.41%	OK
EH1873	*E. coli*	0.00%	OK	81.00x	OK	99.48%	OK	37	OK	36	OK	0.50%	OK	0.50%	OK	0	OK	0	OK	0.20%	OK	0.37%	OK	100.00%	OK	98.41%	OK
EH1882	*E. coli*	1.14%	Warn	64.00x	OK	99.56%	OK	37	OK	36	OK	0.50%	OK	0.50%	OK	0	OK	0	OK	0.17%	OK	0.32%	OK	100.00%	OK	98.41%	OK
EH1923	*E. coli*	0.00%	OK	39.00x	OK	94.43%	Warn	36	OK	36	OK	0.50%	OK	0.50%	OK	0	OK	0	OK	0.22%	OK	0.43%	OK	100.00%	OK	98.41%	OK
EH1965	*E. coli*	0.00%	OK	63.00x	OK	99.08%	OK	36	OK	35	OK	0.50%	OK	0.50%	OK	0	OK	0	OK	0.39%	OK	0.62%	OK	96.41%	OK	82.47%	OK
EH1979	*E. coli*	0.00%	OK	68.00x	OK	99.04%	OK	37	OK	36	OK	0.50%	OK	0.50%	OK	0.02	OK	0	OK	0.19%	OK	0.25%	OK	100.00%	OK	98.41%	OK
EH2015	*E. coli*	0.00%	OK	65.00x	OK	99.56%	OK	37	OK	36	OK	0.50%	OK	0.50%	OK	0	OK	0	OK	0.25%	OK	0.45%	OK	100.00%	OK	96.41%	OK
EH2038	*E. coli*	0.00%	OK	50.00x	OK	99.40%	OK	36	OK	36	OK	0.50%	OK	0.50%	OK	0	OK	0	OK	0.28%	OK	0.42%	OK	100.00%	OK	98.41%	OK
EH925	*E. coli*	0.00%	OK	63.00x	OK	97.37%	OK	37	OK	36	OK	0.50%	OK	0.50%	OK	0	OK	0	OK	0.21%	OK	0.44%	OK	100.00%	OK	98.41%	OK
TIAC1181	*E. coli*	4.32%	Warn	50.00x	OK	99.40%	OK	36	OK	36	OK	0.50%	OK	0.50%	OK	0	OK	0	OK	0.28%	OK	0.58%	OK	100.00%	OK	92.43%	OK
TIAC1182	*E. coli*	3.63%	Warn	40.00x	OK	99.40%	OK	36	OK	36	OK	0.50%	OK	0.50%	OK	0	OK	0	OK	0.31%	OK	0.44%	OK	100.00%	OK	92.43%	OK
TIAC1185	*E. coli*	3.77%	Warn	33.00x	OK	99.52%	OK	37	OK	35	OK	0.50%	OK	0.50%	OK	0	OK	0	OK	0.29%	OK	0.47%	OK	100.00%	OK	92.43%	OK
TIAC1186	*E. coli*	4.51%	Warn	63.00x	OK	99.16%	OK	37	OK	36	OK	0.50%	OK	0.50%	OK	0	OK	0	OK	0.38%	OK	0.46%	OK	100.00%	OK	90.44%	OK

TIAC1188	*E. coli*	4.46%	Warn	53.00x	OK	99.76%	OK	37	OK	36	OK	0.50%	OK	0.50%	OK	0	OK	0	OK	0.28%	OK	0.52%	OK	100.00%	OK	86.45%	OK
TIAC1192	*E. coli*	14.01%	Fail	31.00x	OK	99.40%	OK	37	OK	36	OK	0.50%	OK	0.50%	OK	0.01	OK	0	OK	0.57%	OK	0.70%	OK	100.00%	OK	92.43%	OK
TIAC1193	*E. coli*	4.41%	Warn	41.00x	OK	98.05%	OK	36	OK	36	OK	0.50%	OK	0.50%	OK	0.01	OK	0	OK	0.28%	OK	0.34%	OK	100.00%	OK	98.41%	OK
TIAC1218	*E. coli*	13.48%	Fail	31.00x	OK	98.89%	OK	36	OK	36	OK	1.50%	OK	1.50%	OK	0	OK	0	OK	0.60%	OK	0.65%	OK	100.00%	OK	90.44%	OK
TIAC1220	*E. coli*	31.16%	Fail	100.00x	OK	0.00%	Fail	37	OK	36	OK	0.50%	OK	0.50%	OK	0	OK	0	OK	1.39%	OK	1.42%	OK	86.45%	OK	76.49%	OK
TIAC1221	*E. coli*	33.78%	Fail	224.00x	OK	0.00%	Fail	36	OK	36	OK	0.50%	OK	0.50%	OK	0	OK	0	OK	1.58%	OK	1.52%	OK	86.45%	OK	76.49%	OK
TIAC1223	*E. coli*	35.71%	Fail	220.00x	OK	0.00%	Fail	36	OK	36	OK	0.50%	OK	0.50%	OK	0	OK	0	OK	1.53%	OK	1.61%	OK	98.41%	OK	82.47%	OK
TIAC1226	*E. coli*	34.95%	Fail	205.00x	OK	0.00%	Fail	36	OK	36	OK	0.50%	OK	0.50%	OK	0	OK	0	OK	1.57%	OK	1.41%	OK	92.43%	OK	82.47%	OK
TIAC1227	*E. coli*	33.72%	Fail	201.00x	OK	0.00%	Fail	36	OK	36	OK	0.50%	OK	0.50%	OK	0	OK	0	OK	1.28%	OK	1.29%	OK	86.45%	OK	80.48%	OK
TIAC1228	*E. coli*	9.45%	Fail	42.00x	OK	98.69%	OK	36	OK	35	OK	1.50%	OK	1.50%	OK	0	OK	0	OK	0.54%	OK	0.75%	OK	98.41%	OK	82.47%	OK
TIAC1240	*E. coli*	9.58%	Fail	35.00x	OK	99.36%	OK	36	OK	36	OK	0.50%	OK	1.50%	OK	0	OK	0	OK	0.43%	OK	0.42%	OK	100.00%	OK	92.43%	OK
TIAC1241	*E. coli*	9.36%	Fail	16.00x	Warn	99.36%	OK	36	OK	34	OK	1.50%	OK	1.50%	OK	0	OK	0	OK	1.03%	OK	1.96%	OK	88.45%	OK	64.54%	Warn
TIAC1242	*E. coli*	8.37%	Fail	50.00x	OK	99.48%	OK	36	OK	36	OK	0.50%	OK	0.50%	OK	0	OK	0	OK	0.38%	OK	0.48%	OK	100.00%	OK	92.43%	OK
TIAC1243	*E. coli*	12.87%	Fail	33.00x	OK	99.44%	OK	36	OK	36	OK	1.50%	OK	1.50%	OK	0	OK	0	OK	0.56%	OK	0.59%	OK	100.00%	OK	92.43%	OK
TIAC1244	*E. coli*	12.68%	Fail	31.00x	OK	98.81%	OK	36	OK	36	OK	1.50%	OK	1.50%	OK	0	OK	0	OK	0.52%	OK	0.60%	OK	98.41%	OK	90.44%	OK
TIAC1245	*E. coli*	7.76%	Fail	32.00x	OK	99.40%	OK	36	OK	36	OK	0.50%	OK	0.50%	OK	0	OK	0	OK	0.38%	OK	0.40%	OK	100.00%	OK	94.42%	OK
TIAC1246	*E. coli*	10.16%	Fail	37.00x	OK	99.64%	OK	36	OK	36	OK	0.50%	OK	1.50%	OK	0	OK	0	OK	0.50%	OK	0.44%	OK	100.00%	OK	94.42%	OK
TIAC1247	*E. coli*	14.23%	Fail	29.00x	OK	99.72%	OK	36	OK	36	OK	1.50%	OK	1.50%	OK	0.02	OK	0	OK	0.57%	OK	0.78%	OK	100.00%	OK	98.41%	OK
TIAC1248	*E. coli*	11.49%	Fail	35.00x	OK	99.68%	OK	36	OK	36	OK	1.50%	OK	1.50%	OK	0	OK	0	OK	0.55%	OK	0.58%	OK	100.00%	OK	88.45%	OK

TIAC1354	*E. coli*	0.00%	OK	44.00x	OK	98.97%	OK	36	OK	36	OK	0.50%	OK	0.50%	OK	0	OK	0	OK	0.28%	OK	0.44%	OK	100.00%	OK	94.42%	OK
TIAC1356	*E. coli*	0.00%	OK	37.00x	OK	99.08%	OK	36	OK	36	OK	1.50%	OK	1.50%	OK	0.01	OK	0	OK	0.25%	OK	0.39%	OK	100.00%	OK	98.41%	OK
TIAC1369	*E. coli*	0.00%	OK	36.00x	OK	99.68%	OK	36	OK	36	OK	1.50%	OK	1.50%	OK	0.01	OK	0	OK	0.27%	OK	0.39%	OK	100.00%	OK	96.41%	OK
TIAC1372	*E. coli*	1.68%	Warn	37.00x	OK	99.60%	OK	37	OK	36	OK	1.50%	OK	1.50%	OK	0.01	OK	0	OK	0.25%	OK	0.39%	OK	100.00%	OK	96.41%	OK
TIAC1382	*E. coli*	3.57%	Warn	34.00x	OK	99.40%	OK	37	OK	36	OK	2.50%	Warn	2.50%	Warn	0.01	OK	0	OK	0.36%	OK	0.42%	OK	100.00%	OK	100.00%	OK
TIAC1398	*E. coli*	0.00%	OK	44.00x	OK	99.44%	OK	36	OK	36	OK	1.50%	OK	1.50%	OK	0.01	OK	0	OK	0.24%	OK	0.32%	OK	100.00%	OK	98.41%	OK
TIAC1399	*E. coli*	0.00%	OK	56.00x	OK	99.40%	OK	37	OK	36	OK	0.50%	OK	0.50%	OK	0.01	OK	0	OK	0.22%	OK	0.33%	OK	100.00%	OK	98.41%	OK
TIAC1400	*E. coli*	0.00%	OK	78.00x	OK	99.44%	OK	37	OK	36	OK	0.50%	OK	1.50%	OK	0.02	OK	0	OK	0.17%	OK	0.32%	OK	100.00%	OK	98.41%	OK
TIAC1402	*E. coli*	0.00%	OK	48.00x	OK	99.60%	OK	36	OK	36	OK	0.50%	OK	0.50%	OK	0.01	OK	0	OK	0.24%	OK	0.43%	OK	98.41%	OK	92.43%	OK
TIAC1408	*E. coli*	0.00%	OK	56.00x	OK	99.68%	OK	37	OK	36	OK	0.50%	OK	0.50%	OK	0	OK	0	OK	0.27%	OK	0.63%	OK	100.00%	OK	92.43%	OK
TIAC1411	*E. coli*	0.00%	OK	49.00x	OK	99.72%	OK	36	OK	36	OK	0.50%	OK	0.50%	OK	0	OK	0	OK	0.27%	OK	0.40%	OK	100.00%	OK	94.42%	OK
TIAC1419	*E. coli*	0.00%	OK	49.00x	OK	99.76%	OK	37	OK	36	OK	0.50%	OK	0.50%	OK	0	OK	0	OK	0.19%	OK	0.53%	OK	100.00%	OK	94.42%	OK
TIAC1420	*E. coli*	0.00%	OK	39.00x	OK	99.72%	OK	36	OK	36	OK	0.50%	OK	0.50%	OK	0	OK	0	OK	0.20%	OK	0.47%	OK	100.00%	OK	94.42%	OK
TIAC1426	*E. coli*	0.00%	OK	40.00x	OK	99.44%	OK	37	OK	36	OK	0.50%	OK	1.50%	OK	0.01	OK	0	OK	0.28%	OK	0.36%	OK	100.00%	OK	98.41%	OK
TIAC1428	*E. coli*	0.00%	OK	48.00x	OK	99.76%	OK	36	OK	36	OK	0.50%	OK	0.50%	OK	0.01	OK	0	OK	0.28%	OK	0.41%	OK	100.00%	OK	94.42%	OK
TIAC1433	*E. coli*	5.88%	Fail	32.00x	OK	99.12%	OK	36	OK	36	OK	0.50%	OK	0.50%	OK	0	OK	0	OK	0.37%	OK	0.47%	OK	100.00%	OK	94.42%	OK
TIAC1434	*E. coli*	5.80%	Fail	23.00x	OK	99.84%	OK	37	OK	35	OK	0.50%	OK	0.50%	OK	0	OK	0	OK	0.35%	OK	0.59%	OK	100.00%	OK	88.45%	OK
TIAC1435	*E. coli*	0.00%	OK	64.00x	OK	99.32%	OK	36	OK	36	OK	0.50%	OK	0.50%	OK	0	OK	0	OK	0.21%	OK	0.41%	OK	100.00%	OK	94.42%	OK
TIAC1440	*E. coli*	0.00%	OK	56.00x	OK	99.60%	OK	36	OK	36	OK	0.50%	OK	0.50%	OK	0	OK	0	OK	0.23%	OK	0.41%	OK	98.41%	OK	94.42%	OK

TIAC1442	*E. coli*	0.00%	OK	60.00x	OK	98.89%	OK	37	OK	36	OK	0.50%	OK	0.50%	OK	0	OK	0	OK	0.25%	OK	0.61%	OK	100.00%	OK	92.43%	OK
TIAC1448	*E. coli*	0.00%	OK	49.00x	OK	99.68%	OK	37	OK	36	OK	0.50%	OK	0.50%	OK	0.01	OK	0	OK	0.23%	OK	0.42%	OK	100.00%	OK	98.41%	OK
TIAC1449	*E. coli*	0.00%	OK	32.00x	OK	99.36%	OK	36	OK	36	OK	0.50%	OK	0.50%	OK	0	OK	0	OK	0.23%	OK	0.33%	OK	100.00%	OK	94.42%	OK
TIAC1454	*E. coli*	0.00%	OK	43.00x	OK	99.16%	OK	36	OK	36	OK	0.50%	OK	0.50%	OK	0.01	OK	0	OK	0.20%	OK	0.35%	OK	100.00%	OK	94.42%	OK
TIAC1460	*E. coli*	0.00%	OK	36.00x	OK	99.44%	OK	36	OK	36	OK	0.50%	OK	0.50%	OK	0.01	OK	0	OK	0.24%	OK	0.29%	OK	100.00%	OK	98.41%	OK
TIAC1463	*E. coli*	0.00%	OK	76.00x	OK	99.32%	OK	36	OK	36	OK	0.50%	OK	0.50%	OK	0	OK	0	OK	0.18%	OK	0.45%	OK	98.41%	OK	94.42%	OK
TIAC1464	*E. coli*	0.00%	OK	41.00x	OK	99.32%	OK	36	OK	36	OK	0.50%	OK	0.50%	OK	0	OK	0	OK	0.21%	OK	0.43%	OK	100.00%	OK	92.43%	OK
TIAC1472	*E. coli*	0.00%	OK	44.00x	OK	98.93%	OK	36	OK	36	OK	0.50%	OK	0.50%	OK	0.01	OK	0	OK	0.24%	OK	0.42%	OK	100.00%	OK	94.42%	OK
TIAC1475	*E. coli*	0.00%	OK	38.00x	OK	99.72%	OK	37	OK	36	OK	0.50%	OK	0.50%	OK	0.01	OK	0	OK	0.20%	OK	0.35%	OK	100.00%	OK	94.42%	OK
TIAC1477	*E. coli*	0.00%	OK	56.00x	OK	99.44%	OK	36	OK	36	OK	0.50%	OK	0.50%	OK	0.01	OK	0	OK	0.17%	OK	0.32%	OK	100.00%	OK	96.41%	OK
TIAC1478	*E. coli*	0.00%	OK	40.00x	OK	99.60%	OK	36	OK	36	OK	0.50%	OK	0.50%	OK	0.01	OK	0	OK	0.23%	OK	0.43%	OK	100.00%	OK	96.41%	OK
TIAC1479	*E. coli*	0.00%	OK	27.00x	OK	99.56%	OK	36	OK	36	OK	0.50%	OK	0.50%	OK	0.01	OK	0	OK	0.24%	OK	0.56%	OK	100.00%	OK	96.41%	OK
TIAC1484	*E. coli*	3.26%	Warn	29.00x	OK	99.56%	OK	36	OK	36	OK	0.50%	OK	0.50%	OK	0	OK	0	OK	0.35%	OK	0.61%	OK	100.00%	OK	92.43%	OK
TIAC1507	*E. coli*	0.00%	OK	49.00x	OK	99.44%	OK	37	OK	36	OK	0.50%	OK	0.50%	OK	0	OK	0	OK	0.20%	OK	0.56%	OK	100.00%	OK	94.42%	OK
TIAC1520	*E. coli*	0.00%	OK	42.00x	OK	99.68%	OK	37	OK	36	OK	0.50%	OK	0.50%	OK	0	OK	0	OK	0.16%	OK	0.43%	OK	100.00%	OK	94.42%	OK
TIAC1521	*E. coli*	0.00%	OK	44.00x	OK	99.64%	OK	37	OK	36	OK	0.50%	OK	0.50%	OK	0	OK	0	OK	0.17%	OK	0.73%	OK	100.00%	OK	94.42%	OK
TIAC1522	*E. coli*	0.00%	OK	35.00x	OK	99.60%	OK	37	OK	36	OK	0.50%	OK	0.50%	OK	0	OK	0	OK	0.20%	OK	0.48%	OK	100.00%	OK	94.42%	OK
TIAC1523	*E. coli*	0.00%	OK	41.00x	OK	99.64%	OK	37	OK	36	OK	0.50%	OK	0.50%	OK	0	OK	0	OK	0.19%	OK	0.50%	OK	100.00%	OK	94.42%	OK
TIAC1526	*E. coli*	0.00%	OK	44.00x	OK	98.89%	OK	37	OK	36	OK	0.50%	OK	0.50%	OK	0	OK	0	OK	0.18%	OK	0.55%	OK	100.00%	OK	94.42%	OK
TIAC1527	*E. coli*	0.00%	OK	42.00x	OK	98.85%	OK	37	OK	36	OK	0.50%	OK	0.50%	OK	0	OK	0	OK	0.19%	OK	0.48%	OK	100.00%	OK	94.42%	OK

TIAC1528	*E. coli*	4.57%	Warn	32.00x	OK	99.52%	OK	36	OK	36	OK	1.50%	OK	1.50%	OK	0	OK	0	OK	0.28%	OK	0.35%	OK	100.00%	OK	96.41%	OK
TIAC1544	*E. coli*	5.22%	Fail	32.00x	OK	99.01%	OK	36	OK	36	OK	1.50%	OK	1.50%	OK	0.01	OK	0	OK	0.34%	OK	0.37%	OK	98.41%	OK	94.42%	OK
TIAC1546	*E. coli*	0.00%	OK	39.00x	OK	99.36%	OK	37	OK	36	OK	0.50%	OK	0.50%	OK	0	OK	0	OK	0.24%	OK	0.63%	OK	100.00%	OK	92.43%	OK
TIAC1550	*E. coli*	0.00%	OK	47.00x	OK	99.60%	OK	37	OK	36	OK	0.50%	OK	0.50%	OK	0	OK	0	OK	0.20%	OK	0.50%	OK	100.00%	OK	96.41%	OK
TIAC1551	*E. coli*	0.00%	OK	45.00x	OK	99.60%	OK	37	OK	36	OK	0.50%	OK	0.50%	OK	0	OK	0	OK	0.19%	OK	0.64%	OK	100.00%	OK	94.42%	OK
TIAC1552	*E. coli*	0.00%	OK	52.00x	OK	99.48%	OK	37	OK	36	OK	0.50%	OK	0.50%	OK	0	OK	0	OK	0.20%	OK	0.49%	OK	100.00%	OK	94.42%	OK
TIAC1553	*E. coli*	0.00%	OK	40.00x	OK	99.48%	OK	37	OK	36	OK	0.50%	OK	0.50%	OK	0	OK	0	OK	0.19%	OK	0.68%	OK	100.00%	OK	94.42%	OK
TIAC1558	*E. coli*	3.41%	Warn	23.00x	OK	99.52%	OK	37	OK	36	OK	0.50%	OK	1.50%	OK	0	OK	0	OK	0.29%	OK	0.51%	OK	100.00%	OK	92.43%	OK
TIAC1559	*E. coli*	4.53%	Warn	32.00x	OK	99.56%	OK	37	OK	36	OK	0.50%	OK	1.50%	OK	0	OK	0	OK	0.27%	OK	0.43%	OK	100.00%	OK	94.42%	OK
TIAC1562	*E. coli*	0.00%	OK	48.00x	OK	98.89%	OK	37	OK	36	OK	0.50%	OK	0.50%	OK	0	OK	0	OK	0.19%	OK	0.44%	OK	100.00%	OK	96.41%	OK
TIAC1567	*E. coli*	0.00%	OK	45.00x	OK	99.56%	OK	37	OK	36	OK	0.50%	OK	0.50%	OK	0	OK	0	OK	0.25%	OK	0.48%	OK	100.00%	OK	96.41%	OK
TIAC1568	*E. coli*	0.00%	OK	42.00x	OK	99.56%	OK	37	OK	36	OK	0.50%	OK	0.50%	OK	0	OK	0	OK	0.18%	OK	0.49%	OK	100.00%	OK	96.41%	OK
TIAC1617	*E. coli*	0.00%	OK	38.00x	OK	99.04%	OK	37	OK	36	OK	0.50%	OK	0.50%	OK	0.01	OK	0	OK	0.23%	OK	0.26%	OK	100.00%	OK	98.41%	OK
TIAC1631	*E. coli*	9.38%	Fail	25.00x	OK	99.80%	OK	36	OK	36	OK	1.50%	OK	1.50%	OK	0.01	OK	0	OK	0.53%	OK	0.50%	OK	100.00%	OK	92.43%	OK
TIAC1641	*E. coli*	0.00%	OK	27.00x	OK	99.04%	OK	37	OK	36	OK	1.50%	OK	1.50%	OK	0.02	OK	0	OK	0.24%	OK	0.32%	OK	100.00%	OK	98.41%	OK
TIAC1642	*E. coli*	0.00%	OK	26.00x	OK	99.08%	OK	37	OK	36	OK	1.50%	OK	2.50%	Warn	0.01	OK	0	OK	0.31%	OK	0.39%	OK	100.00%	OK	98.41%	OK
TIAC1653	*E. coli*	0.00%	OK	46.00x	OK	98.97%	OK	37	OK	36	OK	0.50%	OK	0.50%	OK	0	OK	0	OK	0.19%	OK	0.51%	OK	100.00%	OK	96.41%	OK
TIAC1664	*E. coli*	0.00%	OK	42.00x	OK	99.32%	OK	37	OK	36	OK	0.50%	OK	0.50%	OK	0	OK	0	OK	0.19%	OK	0.51%	OK	100.00%	OK	92.43%	OK

TIAC1878	*E. coli*	12.92%	Fail	31.00x	OK	98.01%	OK	37	OK	36	OK	0.50%	OK	0.50%	OK	0	OK	0	OK	0.53%	OK	0.76%	OK	100.00%	OK	92.43%	OK
TIAC1880	*E. coli*	9.46%	Fail	32.00x	OK	98.81%	OK	37	OK	36	OK	0.50%	OK	0.50%	OK	0	OK	0	OK	0.39%	OK	0.44%	OK	100.00%	OK	94.42%	OK
TIAC1881	*E. coli*	11.51%	Fail	30.00x	OK	99.04%	OK	37	OK	37	OK	1.50%	OK	1.50%	OK	0	OK	0	OK	0.91%	OK	0.78%	OK	48.61%	Warn	48.61%	Warn
TIAC1883	*E. coli*	9.84%	Fail	27.00x	OK	99.12%	OK	36	OK	36	OK	0.50%	OK	0.50%	OK	0	OK	0	OK	0.46%	OK	0.52%	OK	98.41%	OK	86.45%	OK
TIAC1884	*E. coli*	10.55%	Fail	27.00x	OK	98.05%	OK	37	OK	36	OK	0.50%	OK	0.50%	OK	0	OK	0	OK	0.46%	OK	0.61%	OK	100.00%	OK	92.43%	OK
TIAC1885	*E. coli*	13.36%	Fail	29.00x	OK	99.72%	OK	37	OK	36	OK	0.50%	OK	0.50%	OK	0	OK	0	OK	0.52%	OK	0.52%	OK	100.00%	OK	92.43%	OK
TIAC1886	*E. coli*	17.52%	Fail	24.00x	OK	98.73%	OK	37	OK	36	OK	0.50%	OK	0.50%	OK	0	OK	0	OK	0.67%	OK	0.77%	OK	100.00%	OK	92.43%	OK
TIAC1887	*E. coli*	10.10%	Fail	38.00x	OK	99.24%	OK	37	OK	36	OK	0.50%	OK	0.50%	OK	0	OK	0	OK	0.41%	OK	0.49%	OK	100.00%	OK	94.42%	OK
TIAC1888	*E. coli*	9.45%	Fail	32.00x	OK	99.44%	OK	37	OK	36	OK	0.50%	OK	1.50%	OK	0	OK	0	OK	0.43%	OK	0.49%	OK	100.00%	OK	94.42%	OK
TIAC1893	*E. coli*	10.44%	Fail	37.00x	OK	92.00%	Warn	36	OK	36	OK	0.50%	OK	0.50%	OK	0	OK	0	OK	0.56%	OK	0.54%	OK	100.00%	OK	90.44%	OK
TIAC1946	*E. coli*	7.47%	Fail	39.00x	OK	92.92%	Warn	37	OK	36	OK	0.50%	OK	0.50%	OK	0	OK	0	OK	0.39%	OK	0.36%	OK	100.00%	OK	98.41%	OK
TIAC1947	*E. coli*	5.12%	Fail	40.00x	OK	99.68%	OK	36	OK	36	OK	0.50%	OK	0.50%	OK	0	OK	0	OK	0.29%	OK	0.31%	OK	100.00%	OK	98.41%	OK
TIAC1951	*E. coli*	0.00%	OK	48.00x	OK	99.52%	OK	36	OK	36	OK	0.50%	OK	0.50%	OK	0	OK	0	OK	0.19%	OK	0.35%	OK	100.00%	OK	98.41%	OK
TIAC1953	*E. coli*	1.57%	Warn	59.00x	OK	99.56%	OK	37	OK	36	OK	0.50%	OK	0.50%	OK	0	OK	0	OK	0.18%	OK	0.32%	OK	100.00%	OK	98.41%	OK
cj_SRR11799713	*C. jejuni*	87.65%	NA	28.00x	OK	0.00%	NA	37	OK	37	OK	18.50%	NA	18.50%	NA	0	OK	0	OK	0.38%	OK	0.38%	OK	100.00%	OK	100.00%	OK
cj_SRR11799714	*C. jejuni*	85.60%	NA	34.00x	OK	0.00%	NA	37	OK	37	OK	18.50%	NA	18.50%	NA	0	OK	0	OK	0.39%	OK	0.39%	OK	100.00%	OK	100.00%	OK
lm_SRR11790964	*L. monocytogenes*	96.87%	NA	79.00x	OK	0.00%	NA	36	OK	36	OK	12.50%	NA	12.50%	NA	0	OK	0	OK	0.52%	OK	0.52%	OK	94.42%	OK	94.42%	OK

lm_SRR11798770	*L. monocytogenes*	96.59%	NA	82.00x	OK	0.00%	NA	37	OK	37	OK	12.50%	NA	12.50%	NA	0	OK	0	OK	0.42%	OK	0.42%	OK	100.00%	OK	100.00%	OK
nm_Z1001	*N. meningitidis*	90.92%	NA	42.00x	OK	0.00%	NA	36	OK	36	OK	1.50%	NA	1.50%	NA	0.01	OK	0.01	OK	0.27%	OK	0.27%	OK	91.03%	OK	91.03%	OK
nm_Z1035	*N. meningitidis*	89.10%	NA	45.00x	OK	0.00%	NA	36	OK	36	OK	1.50%	NA	1.50%	NA	0.01	OK	0.01	OK	0.45%	OK	0.45%	OK	91.03%	OK	91.03%	OK
se_SRR11799638	*S. enterica*	63.66%	NA	38.00x	OK	10.39%	NA	36	OK	36	OK	2.50%	NA	2.50%	NA	0	OK	0	OK	0.20%	OK	0.20%	OK	100.00%	OK	100.00%	OK
se_SRR11799644	*S. enterica*	60.07%	NA	24.00x	OK	10.90%	NA	35	OK	35	OK	2.50%	NA	2.50%	NA	0	OK	0	OK	0.34%	OK	0.34%	OK	100.00%	OK	100.00%	OK
ye_SRR10949351	*Y. enterocolitica*	95.20%	NA	43.00x	OK	0.00%	NA	36	OK	36	OK	2.50%	NA	2.50%	NA	0	OK	0	OK	0.26%	OK	0.26%	OK	98.41%	OK	98.41%	OK
ye_SRR11088743	*Y. enterocolitica*	94.66%	NA	40.00x	OK	0.00%	NA	37	OK	37	OK	2.50%	NA	2.50%	NA	0	OK	0	OK	0.22%	OK	0.22%	OK	100.00%	OK	100.00%	OK

Table S14

Table S14. Overview of validation samples with reads classified as *Bos taurus*. The first and second columns lists the sample name and the percentage of reads classified as *Bos taurus* in case Kraken2 indicated a failure for the QC check. The third column lists the percentage of reads classified as *E. coli* using the Kraken database described in the main manuscript. The last columns lists whether the sample was retained or removed from the validation. Classification was performed using Kraken2 as explained in the Materials and Methods, on a database containing the reference sequences of: *Bos taurus* (GCF_000003055), *Capra hircus* (GCF_001704415), *Chlorocebus sabaeus* (GCF_000409795), *Mesocricetus auratus* (GCF_000349665), *Cavia porcellus* (GCF_000151735), *Equus caballus* (GCF_000002305), *Mus musculus* (GCF_000001635), *Rattus norvegicus* (GCF_000001895), *Ovis aries* (GCF_000298735), and *Sus scrofa* (GCF_000003025).

Sample	Reads classified as *Bos taurus* (%)	Reads classified as *E. coli* (%)	Kept / removed
EH1648	5.40	48.16	Kept
EH1771	5.59	49.71	Kept
TIAC1181	11.68	58.66	Kept
TIAC1182	8.85	60.49	Kept
TIAC1185	9.16	62.28	Kept
TIAC1186	11.40	57.64	Kept
TIAC1188	11.24	56.95	Kept
TIAC1192	33.17	41.57	Kept
TIAC1193	12.08	58.72	Kept
TIAC1218	36.57	39.46	Kept
TIAC1220	84.91	1.82	Removed
TIAC1221	86.01	0	Removed
TIAC1223	87.34	0	Removed
TIAC1226	86.95	0	Removed
TIAC1227	84.75	0	Removed
TIAC1228	28.29	33.15	Kept
TIAC1240	23.63	49.71	Kept
TIAC1241	30.53	41.92	Kept
TIAC1242	21.48	50.09	Kept
TIAC1243	33.53	42.34	Kept
TIAC1244	33.69	39.94	Kept
TIAC1245	18.94	54.72	Kept
TIAC1246	27.31	47.65	Kept
TIAC1247	34.54	41.3	Kept
TIAC1248	31.55	41.89	Kept
TIAC1433	14.94	60.08	Kept
TIAC1434	14.56	60.35	Kept
TIAC1484	8.67	65.24	Kept
TIAC1528	12.04	58.9	Kept

TIAC1544	15.05	56.13	Kept
TIAC1558	9.35	65.48	Kept
TIAC1559	13.10	59.69	Kept
TIAC1631	26.59	46.53	Kept
TIAC1878	32.91	33.33	Kept
TIAC1880	23.52	40.26	Kept
TIAC1881	40.35	14.85	Kept
TIAC1883	28.65	32.43	Kept
TIAC1884	27.88	35.15	Kept
TIAC1885	32.24	33.05	Kept
TIAC1886	41.87	28.22	Kept
TIAC1887	25.22	37.52	Kept
TIAC1888	26.13	49.33	Kept
TIAC1893	28.81	31.58	Kept
TIAC1946	20.65	40.54	Kept
TIAC1947	13.30	43.06	Kept

Table S15

Table S15. PCR detection of AMR genes. Results of the PCRs as described in Supplementary Table S9. All AMR genes in the positive set of the validation were tested. The first and second columns list the sample and AMR gene, respectively. The third, fourth and fifth column contain the results for the different bioinformatics approaches. The last column contains the results for the conventional PCR tests. Presence of the gene is indicated with ‘1’. Absence of the gene is indicated with ‘0’.

Sample	Gene	BLAST+	SRST2	KMA	PCR
EH1227	*aac(3)-IV*	1	1	1	1
	aph(3'')-Ib	1	1	1	1
	aph(4)-Ia	1	1	1	1
	aph(6)-Id	1	1	1	1
	blaTEM	1	1	1	1
	sul2	1	1	1	1
EH1236	*aph(3'')-Ib*	1	1	1	1
	aph(6)-Id	1	1	1	1
	floR	1	1	1	1
EH1260	*aadA1*	1	1	1	1
	ant(2'')-Ia	1	1	1	1
	aph(3')-Ia	1	1	1	1
	aph(3'')-Ib	1	1	1	1
	aph(6)-Id	1	1	1	1
	catA1	1	1	1	1
	sul1	1	1	1	1
	tetA	1	1	1	1
EH1273	*aph(3'')-Ib*	1	1	1	1
	aph(6)-Id	1	1	1	1
EH1348	*aph(3'')-Ib*	1	1	1	1
	aph(6)-Id	1	1	1	1
EH1380	*aph(3')-Ia*	1	1	1	1
	tetA	1	1	1	1
EH1389	tetA	1	1	1	1
EH1533	*aph(3')-Ia*	1	1	1	1
	tetA	1	1	1	1
EH1641	*aac(3)-IV*	1	1	1	1
	aadA1	1	1	1	1
	aph(3')-Ia	1	1	1	1
	aph(3'')-Ib	1	1	1	1
	aph(4)-Ia	1	1	1	1
	aph(6)-Id	1	1	1	1
	blaTEM	1	1	1	1
	dfrA1	1	1	1	1

	sul1	1	1	1	1
	sul2	1	1	1	1
	tetA	1	1	1	1
	tet31	1	1	1	1
EH1671	*aph(3'')-Ib*	1	1	1	1
	aph(6)-Id	1	1	1	1
	blaTEM	1	1	1	1
	sul2	1	1	1	1
EH1733	*aadA1*	1	1	1	1
	aph(3')-Ia	1	1	1	1
	blaTEM	1	1	1	1
	catA1	1	1	1	1
	dfrA1	1	1	1	1
	sul1	1	1	1	1
	tetA	1	1	1	1
EH1757	*aph(3'')-Ib*	1	1	1	1
	aph(6)-Id	1	1	1	1
	sul2	1	1	1	1
EH1766	*aadA1*	1	1	1	1
	aph(3')-Ia	1	1	1	1
	aph(3'')-Ib	1	1	1	1
	aph(6)-Id	1	1	1	1
	blaTEM	1	1	1	1
	catA1	1	1	1	1
	dfrA1	1	1	1	1
	sul1	1	1	1	1
	sul2	1	1	1	1
	tetA	1	1	1	1
EH1783	*aadA1*	1	1	1	1
	aph(3'')-Ib	1	1	1	1
	dfrA1	1	1	1	1
	sul1	1	1	1	1
	sul2	1	1	1	1
	tetA	1	1	1	1
EH1785	*aph(3'')-Ib*	1	1	1	1
	aph(6)-Id	1	1	1	1
	blaTEM	1	1	1	1
	catA1	1	1	1	1
	dfrA17	1	1	1	1
	tetB	1	1	1	1
EH1811	*aadA1*	1	1	1	1
	aph(3'')-Ib	1	1	1	1

	aph(6)-Id	1	1	1	1
	blaTEM	1	1	1	1
	dfrA1	1	1	0	1
	sul1	1	1	1	1
	sul2	1	1	1	1
	tetA	1	1	1	1
EH1813	*aph(3'')-Ib*	1	1	1	1
	aph(6)-Id	1	1	1	1
	blaTEM	1	1	1	1
	sul2	1	1	1	1
EH1815	*aph(3'')-Ib*	1	1	1	1
	aph(6)-Id	1	1	1	1
	sul2	1	1	1	1
EH1829	*aph(3'')-Ib*	1	1	1	1
	aph(6)-Id	1	1	1	1
	blaTEM	1	1	1	1
	catA1	1	1	1	1
	sul2	1	1	1	1
	tetA	1	1	1	1
EH1831	*aph(3'')-Ib*	1	1	1	1
	aph(6)-Id	1	1	1	1
	sul2	1	1	1	1
EH1839	*aph(3'')-Ib*	1	1	1	1
	aph(6)-Id	1	1	1	1
	sul2	1	1	1	1
EH1847	*tetA*	1	1	1	1
EH1923	*aadA1*	1	1	1	1
	cmlA1	1	1	1	1
	sul3	1	1	1	1
EH1979	*aadA1*	1	1	1	1
	aph(3')-Ia	1	1	1	1
	blaOXA-1	1	1	1	1
	sul1	1	1	1	1
	tetA	1	1	1	1
EH925	*aadA1*	1	1	1	1
	aph(3'')-Ib	1	1	1	1
	aph(6)-Id	1	1	1	1
	blaOXA-1	1	1	1	1
	sul1	1	1	1	1
	tetA	1	1	1	1

Table S16

Table S16. Detected AMR point mutations and Sanger sequencing confirmation. This table lists the point mutations that were included in the validation of the AMR prediction assay. The first column lists the sample name. The second and third columns list the gene and mutations, respectively. The last column indicates if the mutation was confirmed through Sanger sequencing.

Sample	Gene	Mutation	Confirmed by Sanger sequencing
EH1733	*gyrA*	S83L	Yes
EH1766	*gyrA*	S83L	Yes
EH1811	*gyrA*	S83L	Yes
EH2015	*parE*	I355T	Yes
EH1260	*gyrA*	S83L	Yes

Table S17

Table S17. Overview of plasmid replicons detected by the online PlasmidFinder tool in the validation samples. The first and second columns list the sample and whether or not the sample is included in the validation, respectively. The next columns list all detected plasmid replicons according to the name in the column header. A ‘1’ denotes that the corresponding plasmid replicon is detected by PlasmidFinder, a ‘0’ denotes that the plasmid replicon is not detected.

Sample	Passes QC	Col156	Col(MG828)	Col(MP18)	ColpVC	IncFIA	IncFIB	IncFII	IncHI2	IncHI2A	IncI1 / IncB/O/K/Z	IncI2	IncQ1	IncX1	IncY	p0111	pEC4115	pKPC-CAV1193
EH1227	TRUE	1	0	0	0	0	1	1	0	0	1	0	0	0	0	1	0	0
EH1236	TRUE	0	0	0	0	0	1	1	0	0	1	0	0	0	0	0	0	0
EH1239	TRUE	1	0	0	0	0	1	0	0	0	1	0	0	0	0	0	0	0
EH1260	TRUE	0	1	0	0	0	1	1	0	0	1	0	0	0	0	0	0	0
EH1273	TRUE	0	0	0	0	0	0	1	0	0	0	0	0	0	0	0	0	0
EH1348	TRUE	0	0	0	0	0	1	1	0	0	0	0	0	0	0	0	0	0
EH1380	TRUE	0	0	0	0	0	1	0	0	0	1	0	0	0	0	0	0	0
EH1389	TRUE	1	0	0	0	0	1	1	0	0	1	0	0	0	1	0	0	1
EH1533	TRUE	0	0	0	0	0	1	0	0	0	1	1	0	0	0	1	0	0
EH1624	TRUE	0	0	0	0	0	1	1	1	1	0	0	0	0	0	0	0	0
EH1641	TRUE	0	0	0	0	0	0	1	0	0	1	0	0	0	0	0	0	0
EH1648	TRUE	0	0	0	0	0	1	0	0	0	0	0	0	0	0	0	0	0
EH1667	TRUE	0	0	0	0	0	1	1	0	0	0	0	0	0	0	0	0	0
EH1671	TRUE	0	0	0	0	0	1	0	0	0	1	0	0	0	0	0	0	0
EH1717	TRUE	0	0	0	0	0	1	0	0	0	0	0	0	0	0	0	0	0
EH1733	TRUE	0	0	0	0	0	1	1	0	0	0	0	0	0	0	1	0	0
EH1757	TRUE	1	0	0	0	0	1	1	0	0	1	0	0	0	0	0	0	0
EH1766	TRUE	0	0	0	0	0	1	0	0	0	1	0	1	0	0	1	0	0
EH1771	TRUE	1	0	0	0	1	0	1	0	0	1	0	0	0	0	0	0	0
EH1782	TRUE	0	0	0	0	0	1	0	0	0	0	0	0	0	0	0	0	0
EH1783	TRUE	0	0	0	0	0	1	0	0	0	1	0	0	0	0	0	0	0
EH1785	TRUE	0	0	0	0	0	1	1	0	0	1	0	1	0	1	0	0	0
EH1811	TRUE	0	0	0	0	0	0	1	0	0	1	0	1	1	1	0	0	0
EH1813	TRUE	1	0	0	1	0	1	1	0	0	0	0	0	0	0	0	0	0
EH1815	TRUE	1	0	0	0	0	1	0	0	0	1	0	0	0	0	1	0	0
EH1819	TRUE	0	0	0	0	1	0	1	0	0	0	0	0	0	0	0	0	0
EH1823	TRUE	0	0	0	0	0	1	0	0	0	1	0	0	0	0	0	0	0
EH1829	TRUE	0	0	0	0	0	1	1	0	0	0	0	1	0	0	0	0	0
EH1831	TRUE	0	0	0	0	0	1	0	0	0	1	0	0	0	0	0	0	0
EH1836	TRUE	0	0	0	0	0	1	1	0	0	0	0	0	0	0	0	0	0

Company Limited by Guarantee l Registered in England No. 1039582 l Registered Office Charles Darwin House, 12 Roger Street, WC1N 2JU, UK
Registered as a Charity: 264017 (England & Wales); SC039250 (Scotland)

EH1839	TRUE	1	0	0	0	0	1	0	0	0	1	0	0	0	0	0	0	0
EH1846	TRUE	0	0	0	0	0	1	0	0	0	1	0	0	0	0	0	0	0
EH1847	TRUE	1	0	0	0	0	0	1	0	0	1	0	0	0	1	0	0	0
EH1858	TRUE	0	0	0	0	0	1	1	0	0	0	0	0	0	0	0	0	0
EH1861	FALSE	1	0	0	0	1	1	1	0	0	0	0	0	0	0	0	0	0
EH1873	TRUE	0	0	0	0	0	0	0	0	0	0	0	0	0	0	0	0	0
EH1882	TRUE	0	0	0	0	0	1	1	0	0	0	0	0	0	0	0	0	0
EH1923	TRUE	1	0	0	0	1	1	1	0	0	0	0	0	0	0	0	0	0
EH1965	TRUE	0	0	0	0	0	1	1	0	0	1	0	0	0	0	0	0	0
EH1979	TRUE	0	0	0	0	0	1	1	0	0	0	0	0	0	0	0	0	0
EH2015	TRUE	0	0	1	0	0	0	1	0	0	1	0	0	0	0	0	0	0
EH2038	FALSE	0	0	0	0	0	1	0	0	0	0	0	0	0	0	0	0	0
EH925	TRUE	0	0	0	0	0	1	0	0	0	1	0	0	0	0	0	0	0
TIAC1181	TRUE	0	0	0	0	1	1	1	0	0	0	0	0	0	0	0	0	0
TIAC1182	TRUE	0	0	0	0	0	1	1	0	0	0	0	0	0	0	0	0	0
TIAC1185	TRUE	0	0	0	0	0	1	1	0	0	0	0	0	0	0	0	0	0
TIAC1186	TRUE	0	0	0	0	0	1	1	0	0	0	0	0	0	0	0	0	0
TIAC1188	TRUE	0	0	0	0	0	1	1	0	0	0	0	0	0	0	0	0	0
TIAC1192	TRUE	0	0	0	0	0	1	1	0	0	0	0	0	0	0	0	0	0
TIAC1193	TRUE	0	0	0	0	0	1	1	0	0	0	0	0	0	0	0	0	0
TIAC1218	TRUE	0	0	0	0	0	1	1	0	0	0	0	0	0	0	0	0	0
TIAC1220	FALSE	0	0	0	0	0	0	0	0	0	0	0	0	0	0	0	0	0
TIAC1221	FALSE	0	0	0	0	0	0	0	0	0	0	0	0	0	0	0	0	0
TIAC1223	FALSE	0	0	0	0	0	0	0	0	0	0	0	0	0	0	0	0	0
TIAC1226	FALSE	0	0	0	0	0	0	0	0	0	0	0	0	0	0	0	0	0
TIAC1227	FALSE	0	1	0	0	0	0	0	0	0	0	0	0	0	0	0	0	0
TIAC1228	TRUE	0	0	0	0	0	0	0	0	0	1	0	1	0	0	0	0	0
TIAC1240	TRUE	0	0	0	0	0	1	1	0	0	0	0	0	0	0	0	0	0
TIAC1241	TRUE	0	0	0	0	1	1	1	0	0	0	0	0	0	0	0	0	0
TIAC1242	TRUE	0	0	0	0	1	1	1	0	0	0	0	0	0	0	0	0	0
TIAC1243	TRUE	0	0	0	0	1	1	1	0	0	0	0	0	0	0	0	0	0
TIAC1244	TRUE	0	0	0	0	1	1	1	0	0	0	0	0	0	0	0	0	0
TIAC1245	TRUE	0	0	0	0	1	1	1	0	0	0	0	0	0	0	0	0	0
TIAC1246	TRUE	0	0	0	0	1	1	1	0	0	0	0	0	0	0	0	0	0
TIAC1247	TRUE	0	0	0	0	0	1	1	0	0	0	0	0	0	0	0	0	0
TIAC1248	TRUE	0	0	0	0	1	1	1	0	0	0	0	0	0	0	0	0	0
TIAC1354	TRUE	0	0	0	0	0	1	1	0	0	0	0	0	0	0	0	0	0
TIAC1356	TRUE	0	0	0	0	0	1	1	0	0	0	0	0	0	0	0	0	0
TIAC1369	TRUE	0	0	0	0	1	1	1	0	0	0	0	0	0	0	0	0	0
TIAC1372	TRUE	0	0	0	0	1	1	1	0	0	0	0	0	0	0	0	0	0
TIAC1382	TRUE	0	0	0	0	0	1	1	0	0	0	0	0	0	0	0	0	0

TIAC1398	TRUE	0	0	0	0	0	1	1	0	0	0	0	0	0	0	0	0	0
TIAC1399	TRUE	0	0	0	0	0	1	1	0	0	0	0	0	0	0	0	0	0
TIAC1400	TRUE	0	0	0	0	0	1	1	0	0	0	0	0	0	0	0	0	0
TIAC1402	TRUE	0	0	0	0	0	1	1	0	0	0	0	0	0	0	0	0	0
TIAC1408	TRUE	0	0	0	0	0	1	1	0	0	0	0	0	0	0	0	0	0
TIAC1411	TRUE	0	0	0	0	0	1	1	0	0	0	0	0	0	0	0	0	0
TIAC1419	TRUE	0	0	0	0	0	1	1	0	0	0	0	0	0	0	0	0	0
TIAC1420	TRUE	0	0	0	0	0	1	1	0	0	0	0	0	0	0	0	0	0
TIAC1426	TRUE	0	0	0	0	0	1	1	0	0	0	0	0	0	0	0	0	0
TIAC1428	TRUE	0	0	0	0	1	1	1	0	0	0	0	0	0	0	0	0	0
TIAC1433	TRUE	0	0	0	0	1	1	1	0	0	0	1	0	0	0	0	0	0
TIAC1434	TRUE	0	0	0	0	0	1	1	0	0	0	0	0	0	0	0	0	0
TIAC1435	TRUE	0	0	0	0	1	1	1	0	0	0	0	0	0	0	0	0	0
TIAC1440	TRUE	0	0	0	0	0	1	1	0	0	0	0	0	0	0	0	0	0
TIAC1442	TRUE	0	0	0	0	0	1	1	0	0	0	0	0	0	0	0	0	0
TIAC1448	TRUE	0	0	0	0	0	1	1	0	0	0	0	0	0	0	0	0	0
TIAC1449	TRUE	0	0	0	0	0	1	1	0	0	0	0	0	0	0	0	0	0
TIAC1454	TRUE	0	0	0	0	0	1	1	0	0	0	1	0	0	1	0	0	0
TIAC1460	TRUE	0	0	0	0	0	1	1	0	0	0	0	0	0	0	0	0	0
TIAC1463	TRUE	0	0	0	0	1	1	1	0	0	0	0	0	0	0	0	0	0
TIAC1464	TRUE	0	0	0	0	0	1	1	0	0	0	0	0	0	0	0	0	0
TIAC1472	TRUE	0	0	0	0	0	1	1	0	0	0	0	0	0	0	0	0	0
TIAC1475	TRUE	0	0	0	0	1	1	1	0	0	0	0	0	0	0	0	0	0
TIAC1477	TRUE	0	0	0	0	0	1	1	0	0	0	0	0	0	0	0	0	0
TIAC1478	TRUE	1	0	0	0	1	1	1	0	0	0	0	0	0	0	0	0	0
TIAC1479	TRUE	1	0	0	0	1	1	1	0	0	0	0	0	0	0	0	0	0
TIAC1484	TRUE	0	0	0	0	0	1	1	0	0	0	0	0	0	0	0	0	0
TIAC1507	TRUE	0	0	0	0	0	1	1	0	0	0	0	0	0	0	0	0	0
TIAC1520	TRUE	0	0	0	0	1	1	1	0	0	0	1	0	0	0	0	0	0
TIAC1521	TRUE	0	0	0	0	0	1	1	0	0	0	0	0	0	0	0	1	0
TIAC1522	TRUE	0	0	0	0	0	1	1	0	0	0	0	0	0	0	0	1	0
TIAC1523	TRUE	0	0	0	0	0	1	1	0	0	0	0	0	0	0	0	1	0
TIAC1526	TRUE	0	0	0	0	0	1	1	0	0	0	0	0	0	0	0	0	0
TIAC1527	TRUE	0	0	0	0	0	1	1	0	0	0	0	0	0	0	0	0	0
TIAC1528	TRUE	0	0	0	0	1	1	1	0	0	0	0	0	0	0	0	0	0
TIAC1544	TRUE	0	0	0	0	0	1	1	0	0	0	0	0	0	0	0	0	0
TIAC1546	TRUE	0	0	0	0	1	1	1	0	0	0	0	0	0	0	0	0	0
TIAC1550	TRUE	0	0	0	0	1	1	1	0	0	0	0	0	0	0	0	0	0
TIAC1551	TRUE	0	0	0	0	1	1	1	0	0	0	0	0	0	0	0	0	0
TIAC1552	TRUE	0	0	0	0	1	1	1	0	0	0	0	0	0	0	0	0	0
TIAC1553	TRUE	0	0	0	0	1	1	1	0	0	0	0	0	0	0	0	0	0

TIAC1558	TRUE	0	0	0	0	1	1	1	0	0	0	0	0	0	0	0	0	0
TIAC1559	TRUE	0	0	0	0	1	1	1	0	0	0	0	0	0	0	0	0	0
TIAC1562	TRUE	0	0	0	0	0	1	1	0	0	0	0	0	0	0	0	0	0
TIAC1567	TRUE	0	0	0	0	0	1	1	0	0	0	0	0	0	0	0	0	0
TIAC1568	TRUE	0	0	0	0	0	1	1	0	0	0	0	0	0	0	0	0	0
TIAC1617	TRUE	0	0	0	0	0	1	1	0	0	0	0	0	0	0	0	0	0
TIAC1631	TRUE	0	0	0	0	0	1	1	0	0	0	0	0	0	0	0	0	0
TIAC1641	TRUE	0	0	0	0	0	1	1	0	0	0	0	0	0	0	0	0	0
TIAC1642	TRUE	0	0	0	0	1	1	1	0	0	0	0	0	0	0	0	0	0
TIAC1653	TRUE	0	0	0	0	0	1	1	0	0	0	0	0	0	0	0	0	0
TIAC1664	TRUE	0	0	0	0	1	1	1	0	0	0	0	0	0	0	0	0	0
TIAC1878	TRUE	0	0	0	0	0	1	0	0	0	0	0	0	0	0	0	0	0
TIAC1880	TRUE	0	0	0	0	0	1	0	0	0	1	0	0	0	0	0	0	0
TIAC1881	TRUE	0	0	0	0	0	0	1	0	0	1	0	0	0	0	0	0	0
TIAC1883	TRUE	1	1	0	0	0	0	1	0	0	0	0	0	0	0	0	0	0
TIAC1884	TRUE	0	0	0	0	0	1	0	0	0	0	0	0	0	0	0	0	0
TIAC1885	TRUE	0	0	0	0	0	1	0	0	0	0	0	0	0	0	0	0	0
TIAC1886	TRUE	0	0	0	0	0	1	0	0	0	1	0	0	0	0	0	0	0
TIAC1887	TRUE	1	1	0	0	0	0	1	0	0	1	0	1	0	0	0	0	0
TIAC1888	TRUE	0	0	0	0	0	1	1	0	0	0	0	0	0	0	0	0	0
TIAC1893	TRUE	1	0	0	0	0	0	1	0	0	0	0	0	0	1	0	0	0
TIAC1946	TRUE	0	0	0	0	0	0	1	0	0	1	0	0	0	0	0	0	0
TIAC1947	TRUE	0	0	0	0	0	0	1	0	0	0	0	0	0	0	0	0	0
TIAC1951	TRUE	0	0	0	0	0	1	1	0	0	1	0	0	0	0	0	0	0
TIAC1953	TRUE	0	0	0	0	0	0	1	0	0	0	0	0	0	0	0	0	0

Table S18

Table S18. Example of FN result for plasmid replicon detection with SRST2. This table lists the plasmid replicons detected by SRST2 in sample EH1831. The first and second columns list the replicon variant and cluster name. The remaining columns lists the statistics extracted from SRST2. In this example, the green hit was selected as the best hit for cluster 90 by the BLAST-based workflow, and the orange hit by SRST2. Due to the higher number of mismatches in the orange hit, the divergence is above the 5% threshold and the hit is subsequently removed, resulting in a FN result for this cluster of replicons. If the green hit would be selected instead, the divergence would be below the threshold and the cluster of replicons would be correctly identified.

Allele	Cluster	Score	Avg depth	Edge1 depth	Edge2 depth	Coverage (%)	Size	Mismatches	Indels
IncFIB(AP001918)_1	30	8.60	21.98	26	4	100.00	682	21	0
IncB/O/K/Z_1	90	13.20	21.98	20	19	100.00	151	6	0
IncB/O/K/Z_3	90	7.36	7.94	7	5	98.68	152	10	3
IncB/O/K/Z_2	90	15.02	21.86	19	0	96.25	160	6	1
IncB/O/K/Z_4	90	6.93	8.06	7	5	100.00	149	11	0
ColRNAI_1	96	448.98	209.39	220	4	100.00	130	11	1
Col(pHAD28)_1	96	489.14	292.37	217	297	100.00	131	10	0
Col440I_1	97	417.08	221.38	226	33	99.12	114	8	2

Company Limited by Guarantee l Registered in England No. 1039582 l Registered Office Charles Darwin House, 12 Roger Street, WC1N 2JU, UK
Registered as a Charity: 264017 (England & Wales); SC039250 (Scotland)

Supplementary Figures

Figure S1

Figure S1. Decision trees for the serotype determination assays. Grey boxes indicate the criteria that are checked, blue boxes denote the resulting serogroup.

O-type

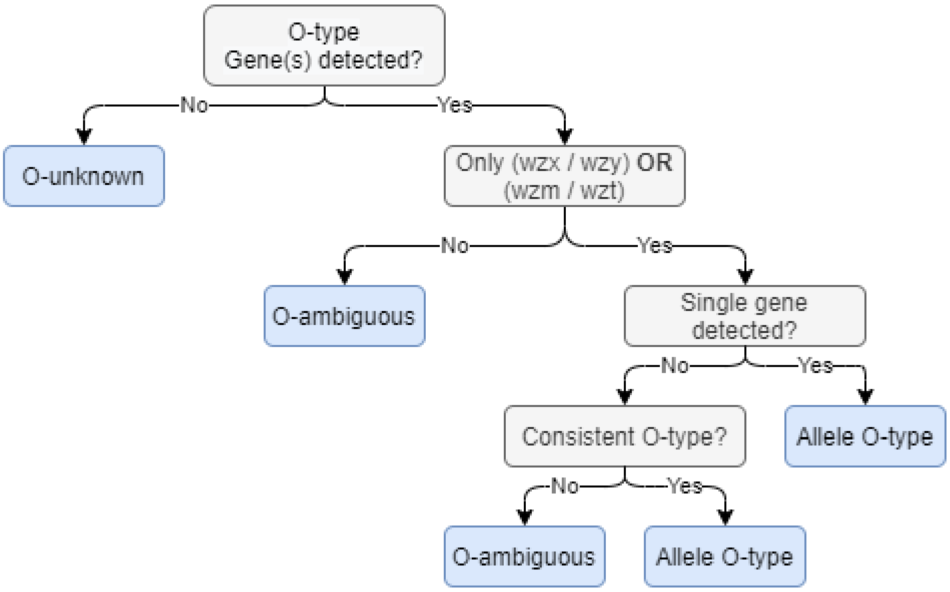

H-type

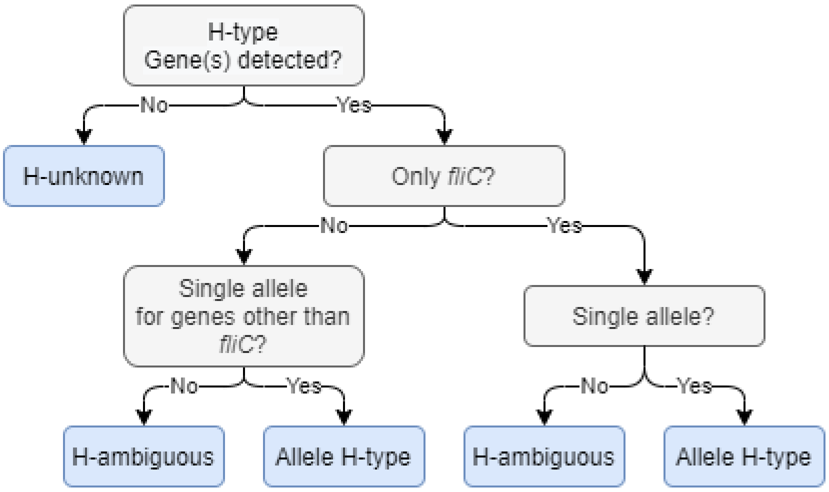

Figure S2

Figure S2. Pipeline interface in Galaxy. The interface is divided in different sections. In the first section, the sample name, read-type (sequencing technology), forward and reverse reads, detection method (BLAST+, SRST2, or KMA), and library kit used for data generation need to be selected. The following sections, ‘quality control’, ‘resistance characterization’, ‘virulence characterization’, ‘serogroup determination’, ‘plasmid replicon detection’ and ‘sequence typing’ each contain different analyses that can be either turned on or off (see Material and methods). By default, all analyses are put on, but users can turn off certain analyses. The last section (‘report’) contains options to include or exclude certain files in the output report. Note that for the sequencing technology, both Illumina and IonTorrent are supported but only Illumina has been validated (see main manuscript).

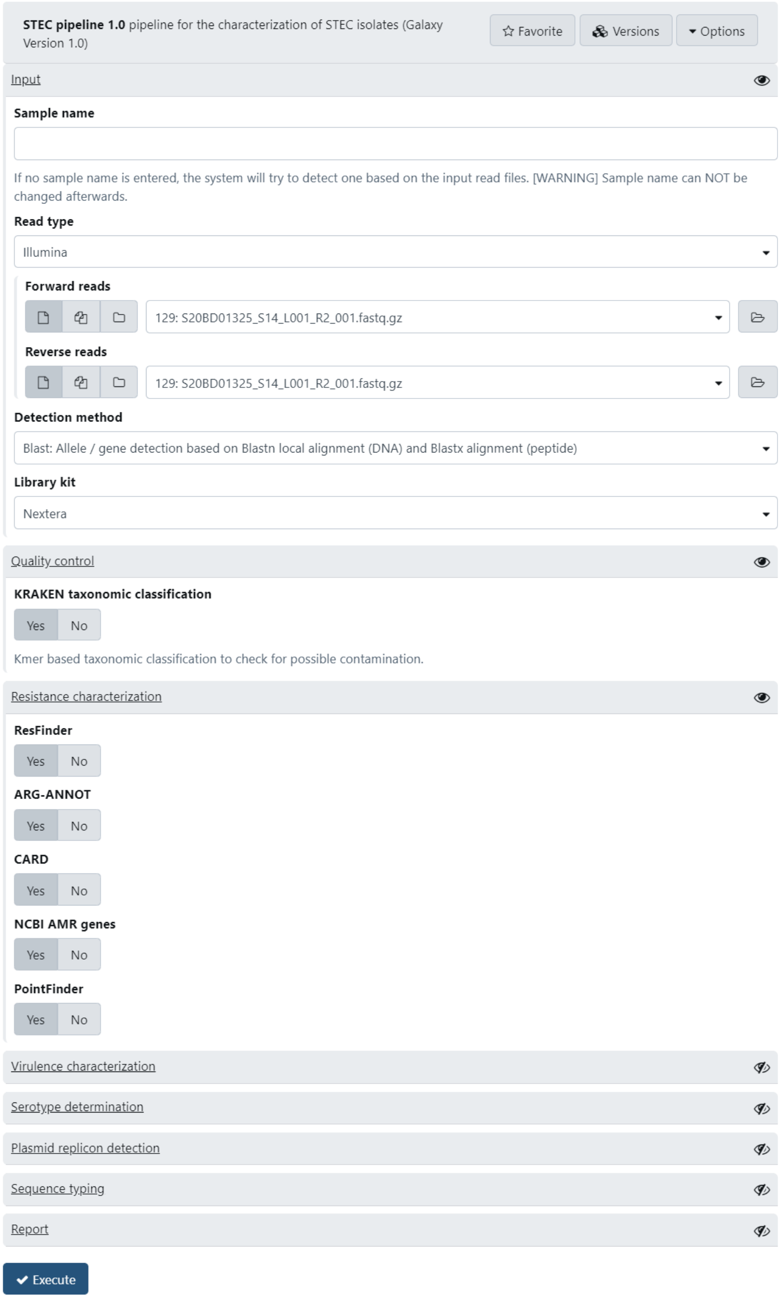

Figure S3

Figure S3. Within-species contamination of sample EH1861. In subplot A, the sample is a clear outlier in terms of total assembly length, with an assembly length far greater than all other samples (the y-axis refers to the sequencing run in which the samples were sequenced). Subplot B shows a part of the mapping of the processed reads against the Sakai O157:H7 reference genome. The X-axis represents the genomic position, the colored bars in the histogram represent positions where the reads differ from the reference sequence, and the bottom of the plot visualizes individual reads mapped to this region. Nucleotides that do not match the reference base are colored (T: red, C: blue, A: green, G: orange). This plot illustrates that the indicated variants are present in ~50% of reads. Subplot C shows the results of the serogroup determination assay, where two different O- and H-types were detected. Subplot D shows the results of the taxonomic classification by Kraken2, with the large majority of reads assigned as *E. coli*. All of these observations support the hypothesis that this sample contains a mix of two different *E. coli* strains.

A) Total assembly length

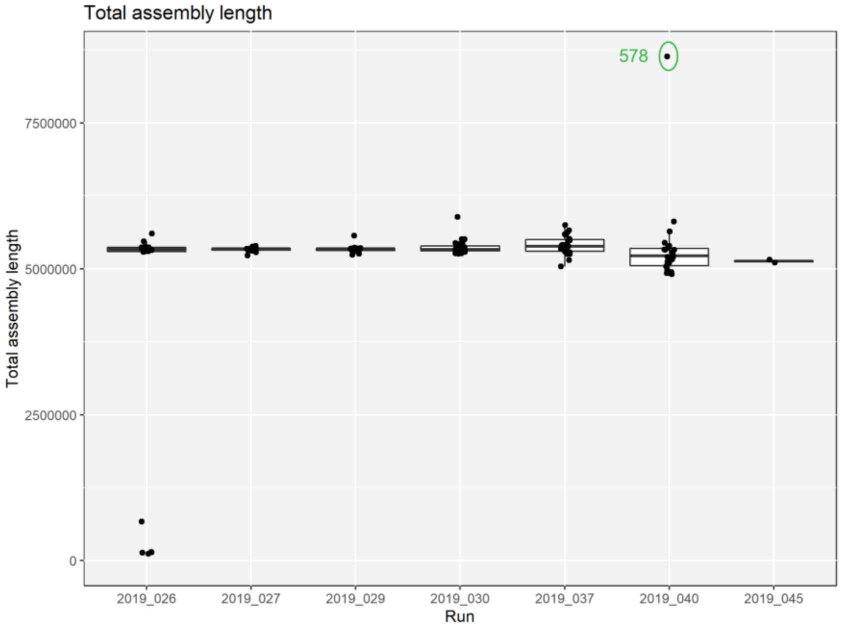

B) Variant calling

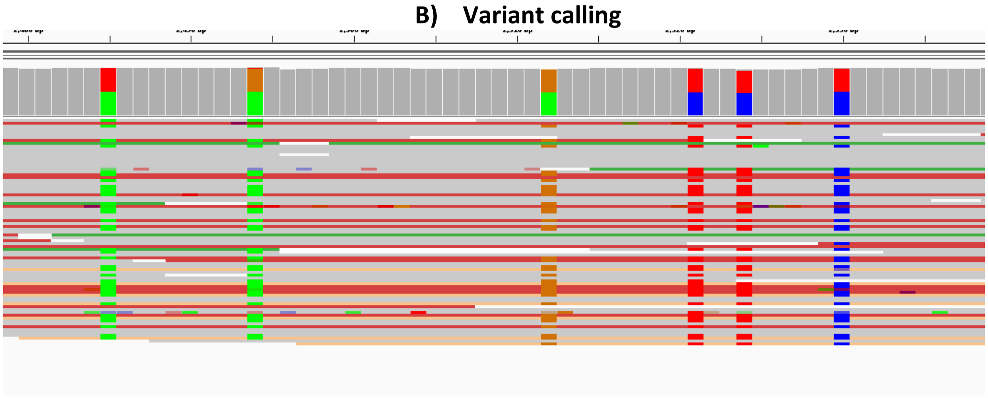

C) Serotype determination

SerotypeFinder - O-type

Locus	% Identity	HSP/Locus length	Contig	Position in contig	Predicted serotype	Accession	Alignment
wzx_69	99.92	1263/1263	NODE_25_length_32411_cov_15.104076	30651..31913	O63	FJ539195	view
wzx_88	99.71	1395/1395	NODE_480_length_5635_cov_5.861656	2880..4274	O84	AB812036	view
wzy_177	99.29	989/1311	NODE_1115_length_1174_cov_4.557784	1..989	O63	EU549862	view

Download (TSV)

Last updated: 03-04-2019

SerotypeFinder - H-type

Locus	% Identity	HSP/Locus length	Contig	Position in contig	Predicted serotype	Accession	Alignment
fliC_2	99.82	1647/1647	NODE_434_length_6233_cov_18.820177	381..2027	H6	AIEY01000041	view
fliC_85	99.13	1494/1494	NODE_131_length_15534_cov_17.108782	3692..5185	H2	AIHA01000023	view

Download (TSV)

Last updated: 03-04-2019

Detected serotype: **O-ambiguous:H-ambiguous**

D) Kraken2 taxonomic classification

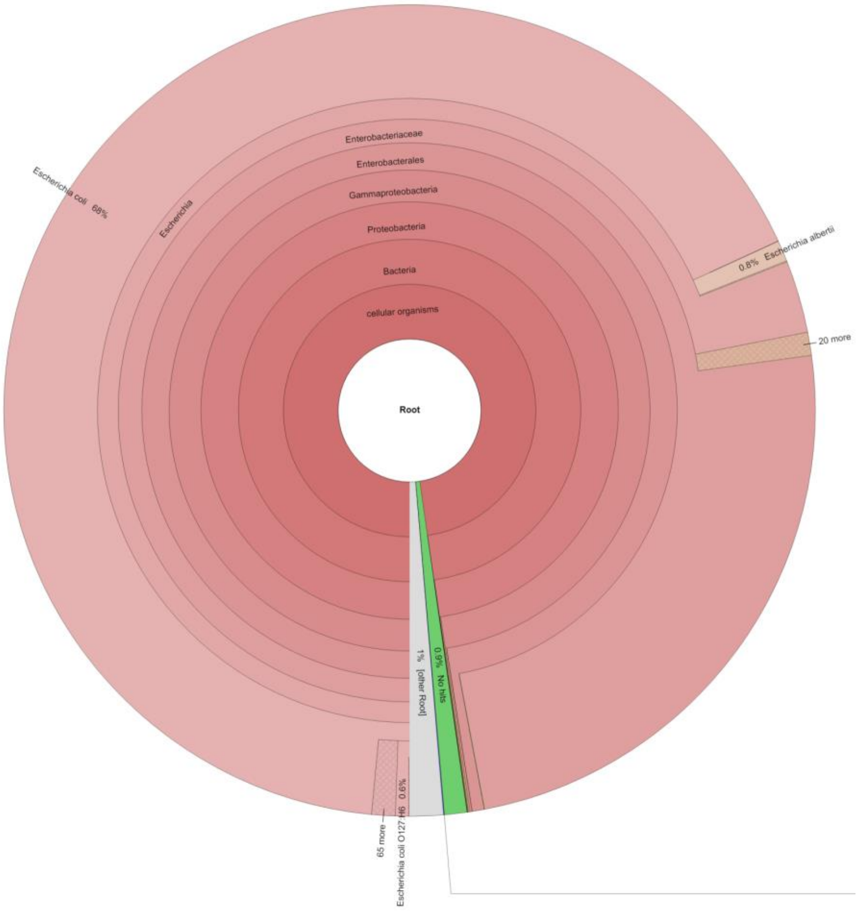

Figure S4

Figure S4. Within-species contamination of sample EH2038. The table at the top shows two *fliC* genes detected in the sample, of which only the perfect match with high depth corresponded to the results from conventional methods (H25). The plot in the bottom shows the reads mapped against the Sakai reference genome and shows variants that are present in circa 10% of the reads. Both observations support the hypothesis that this sample contains a low-level contamination of an *E. coli* sample with a different serotype.

Locus	Length	% Covered	Mismatches	Uncertainty	Divergence (%)	Depth	Predicted serotype
fliC_2	1647	95.20	21snp79holes	depth4.182	1.34	4.18	H6
fliC_311	1332	100.00	-	-	0.00	25.42	H25

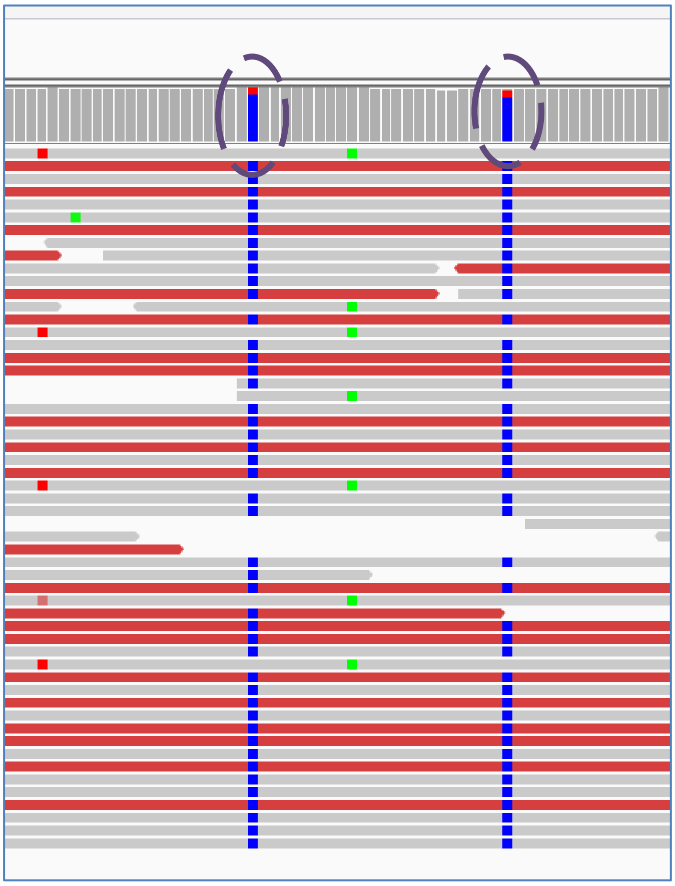

Figure S5

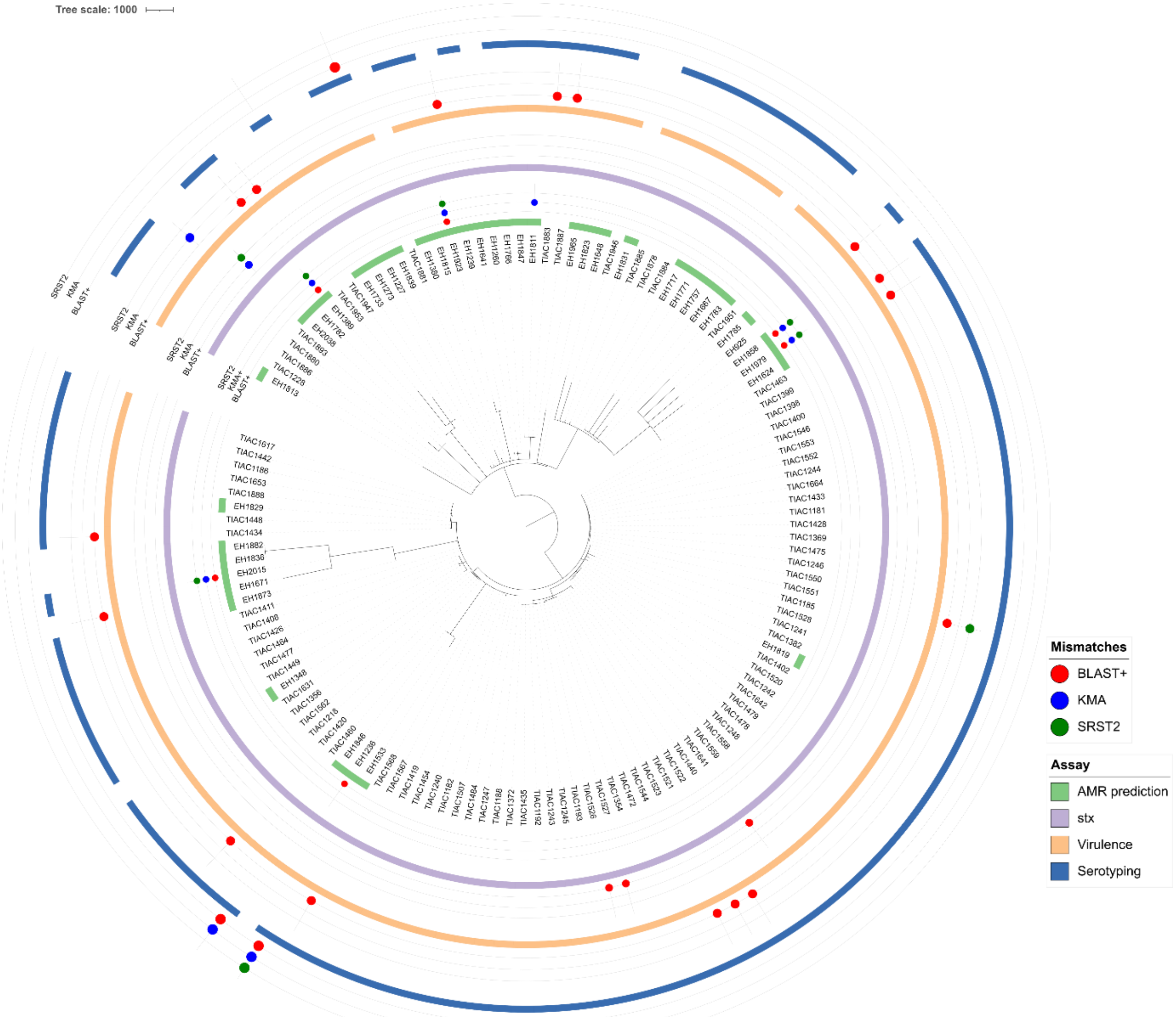

Figure S5. Mismatches for the BLAST+-based detection imposed on the core genome MLST phylogeny containing all isolates included in the validation. Branch lengths correspond to the number of core genome MLST allele differences between samples. The colored strips represent the isolates that were included in the performance evaluation for the corresponding assay. The colored dots represent samples that contained at least one mismatch with the corresponding detection method. A single dot can correspond to multiple mismatches in case of assays where more than one observation is evaluated per sample. Sequence typing mismatches were omitted for clarity, but they occurred in all samples for both BLAST+- and KMA-based detection.

Supplementary Information

Evaluation of dataset quality

Raw and trimmed read counts are listed in Supplementary Table S11, with a median number of 652,868 reads for the in-house sequenced STEC samples. An overview of assembly statistics is provided in Supplementary Table S12. Assembly was problematic for five samples (TIAC1220, TIAC1221, TIAC1223, TIAC1226, and TIAC1227) where the total assembly length was only a small fraction of the expected genome size. One additional sample (EH1861) failed the 'percent cgMLST genes identified' check. Further investigation indicated that this sample contained an approximately equal mix of two *E. coli* strains with a different serotype, resulting in a large fraction of multi-hits for cgMLST loci and a substantially larger total assembly length (see Supplementary Figure S3). These six samples were consequently removed from the validation dataset. Assembly statistics for the remaining samples indicated high quality, with a median total assembly length of 5,328,834 bp and N50 of 167,570 bp. Coverage of the remaining 131 STEC samples varied between 16x and 91x, with a median of 44x. These samples passed all quality control checks (a full overview of all quality control (QC) checks is provided in Supplementary Table S13), except for 34 samples for which Kraken2 classified more than 5% of reads as *Actinoalloteichus* sp. AHMU CJ021. Further investigation indicated that this was a false positive identification caused by a bovine genomic region wrongly incorporated in the corresponding reference genome of this strain (see Supplementary Methods) because for 45 samples a latent presence of *Bos taurus* was present that was originally not detected as this genome is not present in the Kraken database (see Supplementary Table S14), resulting in reads falsely being classified as *Actinoalloteichus* sp. AHMU CJ021 (with 34 of them above the 5% threshold). This most likely originated from the original sample preparation or DNA extraction for this dataset, but, except for the aforementioned five samples, enough *E. coli* reads remained for incorporation in the validation dataset. The dataset for this validation was created based on stored DNA that was extracted for other research projects, some years ago. *B. taurus* was not targeted and thus not detected when the samples were previously characterized by conventional methods [36], highlighting the added value of WGS because of its higher resolution and universal approach. Presence of DNA originating from unexpected species is a re-occurring issue with NGS data, possibly affecting many publicly available datasets and stored and re-used DNA collections [37–39]. However, we did not observe any impact on the performance of the evaluated bioinformatics assays, except for the five aforementioned samples that did not pass the QC checks due to a heavily reduced *E. coli* read yield (see Supplementary Table S14). One sample (EH2038) passed all quality checks, but manual investigation revealed a low-level contamination of an *E. coli* with a different serotype (see Supplementary Figure S4). Since this sample was not flagged by our QC checks, it was nevertheless included in the performance evaluation. In total, 131 out of 137 sequenced STEC samples were retained for validation. Failed quality checks for contaminants, percentage cgMLST genes identified and GC-content deviation were discarded for negative control samples because these checks are tailored specifically to *E. coli*.

Contamination check *Actinoalloteichus* sp. AHMU CJ021

For 34 samples, the Kraken2 contamination check threw a failure indicating that more than 5% of reads were identified for another species than *E. coli* (see Supplementary Table S12), namely *Actinoalloteichus* sp. AHMU CJ021. Further investigation with Kraken2 using the mammalian database however also indicated that for those samples a *Bos taurus* contamination might be present (see Supplementary Table S14). This issue was therefore investigated by mapping trimmed reads against three reference genomes: *Bos taurus* (NCBI accession: NC_037328.1), *E. coli* (NC_002695.2) and *Actinoalloteichus* sp. AHMU CJ021 (accession: CP025990.1) as described in the main manuscript (section 2.1.1). A custom script was then used to determine the overlap between the generated BAM files. This analysis revealed that over 99% of reads mapped to *Actinoalloteichus* also mapped to *Bos taurus*. All reads mapped to the *Actinoalloteichus* reference genome were limited to two genomic regions (CP025990.1:4650000-4651200, CP025990.1:3943000-3943350). Alignment of the sequence of these regions against the NCBI nucleotide (*nt)* database returned several hits for cattle (unpublished results). Visual inspection of the alignment against the *Bos taurus* genome showed that the large majority of reads were approximately randomly distributed, as expected with a regular contamination. The identified contamination with Kraken2 for *Actinoalloteichus* sp. AHMU CJ021 can therefore most likely be explained by a bovine genomic region that was falsely incorporated in the *Actinoalloteichus* sp. AHMU CJ021 reference genome.

Sample	Total read pairs	Pairs mapped to *Actinoalloteichus*	Pairs mapped to *Bos taurus*	Pairs mapped to *E. coli*	Pairs overlap *Bos taurus - Actinoalloteichus*	Pairs overlap *E. coli - Actinoalloteichus*	Pairs overlap *E. coli – Bos taurus*	% Of *Actinoalloteichus* pairs common to *Bos taurus*
EH1227	475918	11	11	325639	0	11	4	0.00
EH1236	544108	30	21	461732	7	23	2	23.33
EH1239	691068	23	10	560032	0	23	4	0.00
EH1260	576416	11	14	408040	0	11	4	0.00
EH1273	701370	24	19	542022	1	23	7	4.17
EH1348	721960	28	21	683077	0	28	20	0.00
EH1380	715724	36	10	543554	0	36	6	0.00
EH1389	628009	15	12	365415	0	15	4	0.00
EH1533	603402	156	408	523978	124	32	5	79.49
EH1624	574225	298	2100	456255	264	33	2	88.59
EH1641	632249	47	68	507550	19	28	4	40.43
EH1648	891427	10478	55940	626847	10439	29	4	99.63
EH1667	746753	359	904	587594	327	32	1	91.09
EH1671	565184	182	450	439888	153	29	3	84.07
EH1717	553077	3398	11950	467977	3369	29	6	99.15
EH1733	1095453	739	5560	879267	664	75	3	89.85
EH1757	836772	343	844	615535	309	34	5	90.09
EH1766	569337	30	75	465646	10	20	4	33.33
EH1771	743264	9466	49661	493753	9434	24	3	99.66
EH1782	1098537	299	617	926123	245	54	3	81.94
EH1783	712513	38	7	600156	0	38	5	0.00
EH1785	755510	106	204	608569	77	29	3	72.64
EH1811	648944	35	27	549429	7	28	4	20.00
EH1813	795054	154	468	626411	131	22	0	85.06
EH1815	584874	4459	14813	459403	4434	23	4	99.44
EH1819	712100	39	13	681371	0	39	12	0.00

EH1823	564287	29	19	462938	0	29	4	0.00
EH1829	771233	43	10	745131	0	41	9	0.00
EH1831	658133	38	14	552190	0	38	5	0.00
EH1836	747140	151	336	554408	113	38	1	74.83
EH1839	653673	27	10	527233	0	27	7	0.00
EH1846	632319	2648	8333	555430	2623	23	6	99.06
EH1847	618004	28	6	515214	0	28	3	0.00
EH1858	882644	178	371	694925	142	36	2	79.78
EH1861	759970	158	335	609063	134	24	1	84.81
EH1873	881150	300	627	744210	263	36	1	87.67
EH1882	715597	112	211	548137	73	39	1	65.18
EH1923	503907	70	143	367662	46	24	0	65.71
EH1965	837238	76	12	721632	0	76	4	0.00
EH1979	816811	30	5	645170	0	30	3	0.00
EH2015	834815	2077	7487	591214	2049	28	2	98.65
EH2038	598954	544	2141	499509	523	21	2	96.14
EH925	681230	146	304	568242	129	17	0	88.36
TIAC1181	696969	18461	94235	581842	18424	24	10	99.80
TIAC1182	551869	12824	56225	479850	12798	19	7	99.80
TIAC1185	438393	10553	46174	380526	10538	11	4	99.86
TIAC1186	903236	26327	120874	755190	26281	36	9	99.83
TIAC1188	765919	22163	101105	643914	22130	29	8	99.85
TIAC1192	610842	54606	232808	365765	54566	17	2	99.93
TIAC1193	575723	15483	81358	478009	15454	22	5	99.81
TIAC1218	703332	56807	295975	365710	56765	14	10	99.93
TIAC1220	642479	132727	611289	16141	132672	3	4	99.96
TIAC1221	1009301	223441	988396	256	223336	2	9	99.95
TIAC1223	756082	173718	742664	671	173670	1	6	99.97
TIAC1226	680516	151746	665912	820	151679	2	8	99.96
TIAC1227	799812	178672	778831	2925	178596	0	3	99.96
TIAC1228	860672	50432	283836	472824	50377	33	8	99.89
TIAC1240	590713	35395	160272	407230	35366	16	7	99.92
TIAC1241	360319	22676	123854	226638	22649	24	10	99.88
TIAC1242	826361	43883	204978	596085	43835	22	17	99.89
TIAC1243	660674	51837	254124	389641	51803	10	7	99.93
TIAC1244	648752	51126	255303	370636	51073	22	6	99.90
TIAC1245	495152	24570	107569	374342	24548	16	5	99.91
TIAC1246	652135	40003	204947	427765	39971	18	9	99.92
TIAC1247	638886	56368	251293	347511	56332	12	5	99.94
TIAC1248	717897	51892	263190	419094	51850	18	10	99.92
TIAC1354	546945	36	58	509763	15	21	4	41.67
TIAC1356	490588	21	32	432949	9	11	3	42.86
TIAC1369	463457	15	6	429976	0	14	6	0.00
TIAC1372	528289	20	10	459815	0	18	10	0.00
TIAC1382	494175	13	5	391122	0	12	3	0.00
TIAC1398	542164	16	9	516993	0	14	4	0.00
TIAC1399	694805	27	2	654876	0	27	1	0.00
TIAC1400	955202	47	9	898572	1	44	7	2.13
TIAC1402	602180	45	6	569691	0	41	4	0.00
TIAC1408	696301	40	89	649280	9	32	7	22.50
TIAC1411	611639	38	5	571730	0	38	4	0.00
TIAC1419	597478	34	19	567014	3	31	5	8.82
TIAC1420	480602	23	57	451281	6	17	10	26.09
TIAC1426	497310	20	5	467983	0	18	3	0.00
TIAC1428	601408	27	9	570903	0	26	8	0.00
TIAC1433	460291	16929	76671	370288	16900	21	10	99.83
TIAC1434	338147	12334	57722	270995	12326	7	7	99.94
TIAC1435	780566	28	9	743677	0	28	7	0.00
TIAC1440	708597	28	5	667403	0	28	2	0.00
TIAC1442	758077	29	29	693928	4	25	10	13.79
TIAC1448	605275	32	60	566380	15	16	8	46.88

TIAC1449	401958	18	19	377470	0	18	3	0.00
TIAC1454	588810	15	17	515371	0	14	10	0.00
TIAC1460	467542	17	6	435716	0	17	5	0.00
TIAC1463	975413	52	22	913846	2	48	8	3.85
TIAC1464	520781	37	6	491961	0	33	5	0.00
TIAC1472	548941	33	8	512746	0	33	6	0.00
TIAC1475	470767	24	4	445433	1	24	4	4.17
TIAC1477	708062	24	7	665224	0	24	7	0.00
TIAC1478	524952	25	5	475393	0	25	5	0.00
TIAC1479	341009	13	4	308856	0	13	1	0.00
TIAC1484	398133	8300	38039	349944	8279	19	9	99.75
TIAC1507	611285	20	23	565540	4	16	13	20.00
TIAC1520	516407	8	14	468436	1	7	5	12.50
TIAC1521	532688	34	62	494530	14	21	8	41.18
TIAC1522	425331	22	27	398011	5	18	5	22.73
TIAC1523	505596	19	44	463061	8	11	3	42.11
TIAC1526	525490	33	122	501685	17	16	5	51.52
TIAC1527	488806	12	10	469893	0	12	4	0.00
TIAC1528	446106	12340	61852	367774	12319	15	6	99.83
TIAC1544	482961	15142	84491	382917	15120	13	1	99.85
TIAC1546	492376	23	24	466596	5	18	3	21.74
TIAC1550	580890	28	39	551775	7	21	8	25.00
TIAC1551	544453	21	27	517956	5	16	5	23.81
TIAC1552	656561	22	17	613562	1	21	6	4.55
TIAC1553	485053	26	52	460524	10	17	11	38.46
TIAC1558	311123	6327	31469	266720	6316	8	8	99.83
TIAC1559	468003	13398	71585	371289	13369	21	6	99.78
TIAC1562	622358	23	10	563319	1	22	3	4.35
TIAC1567	565167	19	17	522776	2	18	4	10.53
TIAC1568	522816	20	27	485966	4	16	5	20.00
TIAC1617	493224	20	7	443612	0	16	6	0.00
TIAC1631	479754	27869	147297	304126	27830	23	6	99.86
TIAC1641	340442	4	4	315644	0	4	3	0.00
TIAC1642	344932	8	12	300374	1	8	9	12.50
TIAC1653	565844	13	23	528568	3	10	6	23.08
TIAC1664	516864	64	224	489297	34	31	14	53.13
TIAC1878	576016	45762	214721	302870	45736	7	9	99.94
TIAC1880	556155	32957	146403	337416	32929	12	4	99.92
TIAC1881	1479376	123402	927422	380433	123249	63	11	99.88
TIAC1883	564007	36185	201793	297285	36136	27	8	99.86
TIAC1884	470786	31079	148208	265681	31046	16	5	99.89
TIAC1885	543278	45696	195066	296892	45663	21	8	99.93
TIAC1886	593471	65728	277245	257463	65694	12	7	99.95
TIAC1887	668289	42067	187797	396513	42025	21	6	99.90
TIAC1888	552719	31869	164018	374846	31846	12	7	99.93
TIAC1893	683970	43163	231800	373517	43121	21	5	99.90
TIAC1946	586752	26003	140660	360355	25975	15	2	99.89
TIAC1947	542347	17164	84007	361228	17148	8	2	99.91
TIAC1951	575243	464	2712	455724	448	16	1	96.55
TIAC1953	674262	142	294	531865	109	33	2	76.76

Detection issues of the IncFIA plasmid replicon with mapping based approaches.

The SRST2 scoring method initially selected the shorter FIA(pBK30683) (295 bp) over the InfFIA_1 replicon variant (388 bp) detected by BLAST, which was in a second stage filtered out due to not making the divergence cutoff. This phenomenon was not observed in the other assays that perform gene detection because genes in the same cluster typically have (approximately) the same length. For KMA, the IncFIA mismatches could be traced back to the percent identity calculation that, in contrast to BLAST, considers the whole gene length causing hits to be filtered when falling <95% percent identity.

Verification detected AMR genes

Predicted resistances (i.e. all FP and TP results) were verified with conventional methods through PCR amplification of the detected AMR genes and regions containing the point mutations. Applied PCR primer sequences, primer concentrations, and PCR conditions are provided in Supplementary Table S9. PCR products were visualized with the TapeStation 4200 instrument, using the D1000 ScreenTape and Reagent kits (Agilent Technologies, Santa Clara, CA) as indicated by the manufacturer's instructions. For verification of AMR point mutations, the respective PCR product was purified using ExoSAP-it PCR Product Cleanup (Applied Biosystems, Foster City, CA), and sequenced using the T7 universal and M13 reverse primers and the BigDye Terminator sequencing kit on a ABI3500 Genetic Analyzer (Applied Biosystems, Foster City, CA). The sequencing results were interpreted using Sequence Scanner Software v1.0 (Applied Biosystems, Foster City, CA).

References

1. **Perelle S, Dilasser F, Grout J, Fach P**. Detection by 5′-nuclease PCR of Shiga-toxin producing Escherichia coli O26, O55, O91, O103, O111, O113, O145 and O157:H7, associated with the world's most frequent clinical cases. *Mol Cell Probes* 2004;18:185–192.
2. **Jothikumar N, Griffiths MW**. Rapid detection of Escherichia coli O157:H7 with multiplex real-time PCR assays. *Appl Environ Microbiol* 2002;68:3169–3171.
3. **Taniuchi M, Walters CC, Gratz J, Maro A, Kumburu H, *et al.*** Development of a multiplex polymerase chain reaction assay for diarrheagenic Escherichia coli and Shigella spp. and its evaluation on colonies, culture broths, and stool. *Diagn Microbiol Infect Dis* 2012;73:121–128.
4. **Aranda KRS, Fagundes-Neto U, Scaletsky ICA**. Evaluation of multiplex PCRs for diagnosis of infection with diarrheagenic Escherichia coli and Shigella spp. *J Clin Microbiol* 2004;42:5849–5853.
5. **Boisen N, Scheutz F, Rasko DA, Redman JC, Persson S, *et al.*** Genomic characterization of enteroaggregative Escherichia coli from children in Mali. *J Infect Dis* 2012;205:431–444.
6. **Tzschoppe M, Martin A, Beutin L**. A rapid procedure for the detection and isolation of enterohaemorrhagic Escherichia coli (EHEC) serogroup O26, O103, O111, O118, O121, O145 and O157 strains and the aggregative EHEC O104:H4 strain from ready-to-eat vegetables. *Int J Food Microbiol* 2012;152:19–30.
7. **Bugarel M, Beutin L, Fach P**. Low-density macroarray targeting non-locus of enterocyte effacement effectors (nle genes) and major virulence factors of Shiga toxin-producing Escherichia coli (STEC): A new approach for molecular risk assessment of STEC isolates. *Appl Environ Microbiol* 2010;76:203–211.
8. **Nielsen EM, Andersen MT**. Detection and characterization of verocytotoxin-producing Escherichia coli by automated 5′ nuclease PCR assay. *J Clin Microbiol* 2003;41:2884–2893.
9. **Leyton DL, Leyton DL, Sloan J, Sloan J, Hill RE, *et al.*** Transfer Region of pO113 from Enterohemorrhagic. *Society* 2003;71:6307–6319.
10. **Bugarel M, Beutin L, Martin A, Gill A, Fach P**. Micro-array for the identification of Shiga toxin-producing Escherichia coli (STEC) seropathotypes associated with Hemorrhagic Colitis and Hemolytic Uremic Syndrome in humans. *Int J Food Microbiol* 2010;142:318–329.
11. **Li D, Liu B, Chen M, Guo D, Guo X, *et al.*** A multiplex PCR method to detect 14 Escherichia coli serogroups associated with urinary tract infections. *J Microbiol Methods* 2010;82:71–77.
12. **Lin A, Sultan O, Lau HK, Wong E, Hartman G, *et al.*** O serogroup specific real time PCR assays for the detection and identification of nine clinically relevant non-O157 STECs. *Food Microbiol* 2011;28:478–483.
13. **Iguchi A, Iyoda S, Seto K, Morita-Ishihara T, Scheutz F, *et al.*** Escherichia coli O-genotyping PCR: A comprehensive and practical platform for molecular O serogrouping. *J Clin Microbiol* 2015;53:2427–2432.
14. **Lin A, Kase JA, Moore MM, Son I, Tran N, *et al.*** Multilaboratory validation of a luminex microbead-based suspension array for the identification of the 11 most clinically relevant Shiga toxin-producing Escherichia coli O serogroups. *J Food Prot* 2013;76:867–870.
15. **Madic J, Peytavin De Garam C, Vingadassalon N, Oswald E, Fach P, *et al.*** Simplex and multiplex real-time PCR assays for the detection of flagellar (H-antigen) fliC alleles and intimin (eae) variants associated with enterohaemorrhagic Escherichia coli (EHEC) serotypes O26:H11, O103:H2, O111:H8, O145:H28 and O157:H7. *J Appl Microbiol* 2010;109:1696–1705.
16. **Banjo M, Iguchi A, Seto K, Kikuchi T, Harada T, *et al.*** Escherichia coli H-Genotyping PCR: A complete and practical platform for molecular h typing. *J Clin Microbiol* 2018;56:1–13.

17. **Beutin L, Delannoy S, Fach P**. Sequence variations in the flagellar antigen genes fliCH25 and fliCH28 of Escherichia coli and their use in identification and characterization of enterohemorrhagic E. Coli (EHEC) O145:H25 and O145:H28. *PLoS One* 2015;10:1–15.
18. **Sundin GW**. Distinct recent lineages of the strA-strB streptomycin-resistance genes in clinical and environmental bacteria. *Curr Microbiol* 2002;45:63–69.
19. **Overbeek LS van, Wellington EMH, Egan S, Smalla K, Heuer H, *et al.*** Prevalence of streptomycin-resistance genes in bacterial populations in European habitats. (Special issue: The contribution of mobile genetic elements in bacterial adaptability and diversity.). *FEMS Microbiol Ecol* 2002;42:277-288. 53 ref.
20. **Hochhut B, Lotfi Y, Mazel D, Faruque SM, Woodgate R, *et al.*** Molecular analysis of antibiotic resistance gene clusters in vibrio cholerae O139 and O1 SXT constins. *Antimicrob Agents Chemother* 2001;45:2991–3000.
21. **Perreten V, Boerlin P**. A New Sulfonamide Resistance Gene (sul3)in Escherichia coli Is Widespread. *Antimicrob Agents Chemother* 2003;47:1169–1172.
22. **Schwarz S, Kehrenberg C, Salmon SA, Watts JL**. In vitro activities of spectinomycin and comparator agents against Pasteurella multocida and Mannheimia haemolytica from respiratory tract infections of cattle. *J Antimicrob Chemother* 2004;53:379–382.
23. **Lanz R, Kuhnert P, Boerlin P**. Antimicrobial resistance and resistance gene determinants in clinical Escherichia coli from different animal species in Switzerland. *Vet Microbiol* 2003;91:73–84.
24. **Zou LK, Wang HN, Zeng B, Zhang AY, Li JN, *et al.*** Phenotypic and genotypic characterization of β-lactam resistance in Klebsiella pneumoniae isolated from swine. *Vet Microbiol* 2011;149:139–146.
25. **Ng LK, Martin I, Alfa M, Mulvey M**. Multiplex PCR for the detection of tetracycline resistant genes. *Mol Cell Probes* 2001;15:209–215.
26. **Guerra B, Soto SM, Argüelles JM, Mendoza MC**. Multidrug resistance is mediated by large plasmids carrying a class 1 integron in the emergent Salmonella enterica serotype [4,5,12:i:-]. *Antimicrob Agents Chemother* 2001;45:1305–1308.
27. **Guerra B, Junker E, Schroeter A, Malorny B, Lehmann S, *et al.*** Phenotypic and genotypic characterization of antimicrobial resistance in German Escherichia coli isolates from cattle, swine and poultry. *J Antimicrob Chemother* 2003;52:489–492.
28. **Sugumar M, Kumar KM, Manoharan A, Anbarasu A, Ramaiah S**. Detection of OXA-1 β-lactamase gene of Klebsiella pneumoniae from blood stream infections (BSI) by conventional PCR and in-silico analysis to understand the mechanism of OXA mediated resistance. *PLoS One* 2014;9:1–8.
29. **Bojesen AM, Bager RJ, Ifrah D, Aarestrup FM**. The rarely reported tet(31) tetracycline resistance determinant is common in Gallibacterium anatis. *Vet Microbiol* 2011;149:497–499.
30. **Lei Y, Qiong C, Changqin H, Shaohong J**. Analysis of antibiotic resistance profile and gene patterns for aminoglycoside modifying enzymes in 64 strains of {\sl Escherichia coli}. *Zhonghua wei sheng wu xue he mian yi xue za zhi = Zhonghua weishengwuxue he mianyixue zazhi* 2005;25:727–732.
31. **Toro CS, Farfán M, Contreras I, Flores O, Navarro N, *et al.*** Genetic analysis of antibiotic-resistance determinants in multidrug-resistant Shigella strains isolated from Chilean children. *Epidemiol Infect* 2005;133:81–86.
32. **Li Y, Liao H, Yao H**. Prevalence of antibiotic resistance genes in air-conditioning systems in hospitals, farms, and residences. *Int J Environ Res Public Health*;16. Epub ahead of print 2019. DOI: 10.3390/ijerph16050683.

33. **Kim HC, Jang J, Kim H, Kim Y, Lee K, *et al.*** Multiplex PCR for Simultaneous Detection of Aminoglycoside Resistance Genes in Escherichia coli and Klebsiella pneumoniae. *Korean J Clin Lab Sci* 2012;44:155–165.
34. **Johnning A, Kristiansson E, Fick J, Weijdegård B, Larsson DGJ**. Resistance mutations in gyrA and parC are common in Escherichia communities of both fluoroquinolone-polluted and uncontaminated aquatic environments. *Front Microbiol* 2015;6:1–9.
35. **Yang H, Duan G, Zhu J, Zhang W, Xi Y, *et al.*** Prevalence and characterisation of plasmid-mediated quinolone resistance and mutations in the gyrase and topoisomerase IV genes among Shigella isolates from Henan, China, between 2001 and 2008. *Int J Antimicrob Agents* 2013;42:173–177.
36. **Barbau-piednoir E, Denayer S, Botteldoorn N, Dierick K, Keersmaecker SCJ De, *et al.*** Detection and discrimination of five E . coli pathotypes using a combinatory SYBR ® Green qPCR screening system. 2018;3267–3285.
37. **Salter SJ, Cox MJ, Turek EM, Calus ST, Cookson WO, *et al.*** Reagent and laboratory contamination can critically impact sequence-based microbiome analyses. *BMC Biol* 2014;12:1–12.
38. **Mukherjee S, Huntemann M, Ivanova N, Kyrpides NC, Pati A**. Large-scale contamination of microbial isolate genomes by illumina Phix control. *Stand Genomic Sci* 2015;10:1–4.
39. **Lusk RW**. Diverse and widespread contamination evident in the unmapped depths of high throughput sequencing data. *PLoS One*;9. Epub ahead of print 2014. DOI: 10.1371/journal.pone.0110808.
